# High mortality rates in a juvenile free‐ranging marine predator and links to dive and forage ability

**DOI:** 10.1002/ece3.5905

**Published:** 2019-12-11

**Authors:** Sam L. Cox, Matthieu Authier, Florian Orgeret, Henri Weimerskirch, Christophe Guinet

**Affiliations:** ^1^ Centre d'Etudes Biologique de Chizé UMR 7372 ‐ CNRS & Universitié de La Rochelle Villiers‐en‐Bois France; ^2^ UMR MARBEC Station Ifremer Séte Séte France; ^3^ Centre National d'Études Spatiales (CNES) Toulouse France; ^4^ Observatoire PELAGIS UMS 3462 ‐ Universitié de La Rochelle & CNRS La Rochelle France; ^5^ ADERA Pessac Cedex France

**Keywords:** bio‐logging, early life, foraging ecology, juvenile mortality, *Mirounga leonina*, southern elephant seal, survival analyses

## Abstract

1. High juvenile mortality rates are typical of many long‐lived marine vertebrate predators. Insufficient development in dive and forage ability is considered a key driver of this. However, direct links to survival outcome are sparse, particularly in free‐ranging marine animals that may not return to land.
2. In this study, we conduct exploratory investigations toward early mortality in juvenile southern elephant seals *Mirounga leonina*. Twenty postweaning pups were equipped with (a) a new‐generation satellite relay data tag, capable of remotely transmitting fine‐scale behavioral movements from accelerometers, and (b) a location transmitting only tag (so that mortality events could be distinguished from device failures). Individuals were followed during their first trip at sea (until mortality or return to land). Two analyses were conducted. First, the behavioral movements and encountered environmental conditions of nonsurviving pups were individually compared to temporally concurrent observations from grouped survivors. Second, common causes of mortality were investigated using Cox's proportional hazard regression and penalized shrinkage techniques.
3. Nine individuals died (two females and seven males) and 11 survived (eight females and three males). All but one individual died before the return phase of their first trip at sea, and all but one were negatively buoyant. Causes of death were variable, although common factors included increased horizontal travel speeds and distances, decreased development in dive and forage ability, and habitat type visited (lower sea surface temperatures and decreased total [eddy] kinetic energy).
4. For long‐lived marine vertebrate predators, such as the southern elephant seal, the first few months of life following independence represent a critical period, when small deviations in behavior from the norm appear sufficient to increase mortality risk. Survival rates may subsequently be particularly vulnerable to changes in climate and environment, which will have concomitant consequences on the demography and dynamics of populations.

## Graphical abstract

High juvenile mortality rates are typical of many long‐lived marine predators and are likely linked to insufficient development of dive and forage ability. Here, we investigate such processes in juvenile southern elephant seals using a new generation of bio‐logging tags, capable of remotely transmitting behavioral information through the Argos satellite system. We found that causes of early mortality during the first year of life were linked to decreased development in dive and forage ability alongside a failure to locate suitable foraging habitat.

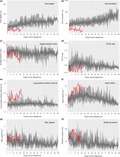

## 1 INTRODUCTION

For long‐lived marine vertebrate predators, insufficient dive and forage ability are considered key drivers of mortality during juvenile and immature stages (Daunt, Afanasyev, Adam, Croxall, & Wanless, 2007; Orgeret, Weimerskirch, & Bost, 2016). In many species, these need to develop rapidly, as individuals quickly transition from full parental care and provisioning to complete independence (Breed, Bowen, & Leonard, 2013; Costa, 1991; Reiter, Stinson, & Boeuf, 1978). The first trip made at sea alone may thus prove a critical time for survival, as individuals must either learn, learned, or have inherited advanced dive and forage abilities so as to be able to exploit the large heterogeneous environments typically inhabited (Carter et al., 2017; de Grissac, Bartumeus, Cox, & Weimerskirch, 2017; Orgeret et al., 2016; Riotte‐Lambert & Weimerskirch, 2013). This may include physiological capability and foraging efficiency, knowledge of habitats and prey distribution, alongside predator avoidance strategies. Moreover, during this time, perturbations in environmental conditions can have disproportionate impacts on fitness, as individuals are already pushing their capabilities as far as they will go, leaving little room to adapt (Burns, 1999; Lea et al., 2009). Indeed, in the early years of life following transition to independence, survival rates often increase with age as dive and forage abilities advance (McMahon, New, Fairley, Hindell, & Burton, 2015; Oro, Torres, Rodriguez, & Drummond, 2010; Pistorius & Bester, 2002).

Much of what is known about the factors impacting first‐year survival stems from studies linking resightings data to an individual's condition at departure from its natal site. For example, in pinnipeds, increased girth, length, weight, and level/quality of parental provision are known to impact first‐year survival (Baker, 2008; Hall, McConnell, & Barker, 2001; McMahon et al., 2015), which likely relates to initial dive capabilities, development, and subsequent restrictions on foraging ability (Hindell et al., 1999; Irvine, Hindell, Hoff, & Burton, 2000). Juvenile survival rates have also been linked to climatic indices (e.g., El Nino Southern Oscillation), possibly due to changes in the abundance and distribution of resources, which can impact parental provisioning during lactation (and thus initial condition), alongside prey availability post‐weaning (Beauplet, Barbraud, Chambellant, & Guinet, 2005; McMahon & Burton, 2005). However, beyond such broadscale correlations there is a sparsity of studies describing and quantifying the fine‐scale dive and forage capabilities of juvenile marine predators in relation to survival (Hazen et al., 2012). This reflects difficulties in obtaining detailed behavioral measurements of far‐ranging individuals that may not return to land.

Recent advances in bio‐logging technologies have substantially increased our ability to observe the fine‐scale behaviors, movements, and physiology of marine species (Hart & Hyrenbach, 2009; Hazen et al., 2012; Volpov et al., 2015). Moreover, new methods in data abstraction and transmission mean device retrieval is no longer obligatory to obtain the information these instruments record (Cox et al., 2018). For pinniped species, it is now possible to remotely track a suite of dive and foraging metrics such as prey catch attempts (PrCA), swimming effort, dive shape, and surface interval (Cox et al., 2018; Heerah, Cox, Blevin, Guinet, & Charrassin, 2019; Photopoulou, Lovell, Fedak, Thomas, & Matthiopoulos, 2015). As such, the early foraging strategies of free‐ranging juveniles that may not return to land can now be observed and, when coupled with double tagging techniques (Drymon & Wells, 2017; Hays, Bradshaw, James, Lovell, & Sims, 2007), examined in relation to survival outcome to gain a more mechanistic understanding of how the two may be related.

Here, we present results from a unique dataset obtained via a new generation of satellite relay data tags, capable of recording and remotely transmitting fine‐scale behavioral movements of 20 juvenile southern elephant seals *Mirounga leonina* during their first trip at sea following weaning. We use these data to conduct exploratory investigations of the potential factors influencing early mortality events. Specifically, we (a) individually compare time‐series data of behavioral movements and encountered environmental conditions of non‐surviving individuals to those of grouped survivors (which successfully completed their first trip at sea following weaning) and (b) statistically assess potential common causes of mortality across the sample population.

Southern elephant seals are wide‐ranging (circumpolar Southern Ocean distribution) meso‐predators that exploit vast heterogeneous environments subject to climatic variation (Hindell et al., 2016). Females give birth to a single pup at the beginning of the austral summer (September–November) which, following a short period (~22 days) of full maternal provisioning (lactation and suckling), is weaned and abandoned at the natal colony (Guinet, Jouventin, & Weimerskirch, 1992). Pups then undergo a period of fasting (~3–9 weeks; Arnbom, Fedak, Boyd, & McConnell, 1993; Guinet et al., 1992), after which they depart for their first trip at sea (duration ~6 months) and learn to forage independently without external input. First‐year survival rates are low (~40%–60%, sometimes below 30%; McMahon, Burton, & Bester, 1999; McMahon et al., 2015; Pistorius & Bester, 2002) and considered a strong determinant of overall population fitness (McMahon, Hindell, Burton, & Bester, 2005). At the time of departure for their first trip at sea, weaned pups are sufficiently large (~140 cm and ~80–100 kg; Arnbom et al., 1993; Guinet, 1994) to be safely equipped with biologging devices for several months (McMahon, Field, Bradshaw, White, & Hindell, 2008).

## 2 METHODS

### 2.1 Tag deployment

Fieldwork was conducted at the Kerguelen Islands, sub‐Antarctic (49°20′S, 70°20′E), during November/December 2014. A total of 20 (10 female and 10 male) weaned (<3 months old) juvenile southern elephant seals (mean mass = 79.9 ± 17.7 kg, length = 139.1 ± 10.6 cm; ±standard deviation; Supporting Information S.1, Table S1) were equipped with (a) a custom‐designed Argos relay satellite tag (SPLASH10‐F‐2961‐DSA tag, Wildlife Computers, hereafter “DSA” tag) and (b) a smart position transmitting tag (SPOT 293A, Wildlife Computers, hereafter “SPOT” tag). Animals were captured and anesthetized (80% of tagged individuals) using a 1:1 combination of tiletamine and zolazepam (Zoletil 100), injected intravenously. Using quick‐setting epoxy (Araldite AW 2101; Ciba), the DSA tag was attached to the fur of the top of the head of a pup and the SPOT tag to its center back. All fieldwork activities were approved by the Comité Environnement et le Préfet des Terre Australes et Antarctiques Françaises.

### 2.2 Tag specifications

The DSA tag measured 86 × 85 × 29 mm and weighed 192 g, which is <1% of the mean mass of the juvenile southern elephant seals in our study. The design of the device was similar to that from investigative studies demonstrating that these tags do not adversely impact mass gain or survival of equipped individuals (McMahon et al., 2008). It comprised an Argos transmitter, pressure sensor (1 Hz sampling rate, resolution 0.5 m ± 1 m +1% of a reading), tri‐axial accelerometer (16 Hz sampling rate), thermistor (1 Hz sampling rate, resolution 0.1°C ± 0.1°C), and wet–dry sensor. These functioned intermittently such that one complete dive (max depth >15 m and duration >60 s) was sampled every ~2.25 hr. Measurements were processed onboard the tag to provide a “per‐dive” summary, which was later transmitted (alongside an Argos‐derived location) via the Argos satellite system. This included the depths and durations of five broken‐stick segments of a dive, alongside the total swimming effort (summed filtered accelerations associated with flipper movements across the lateral axis of the accelerometer; Richard et al., 2014) and time spent in PrCA behaviors (total time during which accelerations reflected “jerk”‐like movements; Viviant, Trites, Rosen, Monestiez, & Guinet, 2010) across each segment. Surface intervals between each sampled dive and the next were also transmitted, alongside sea surface temperature (SST; at ~2‐3 m). An in‐depth description of onboard processing algorithms and DSA tag functionality can be found in Cox et al. (2018). The Argos only transmitting SPOT tag measured 72 × 54 × 24 mm and weighed 119 g. From this, an Argos‐derived location was obtained several times per day, such that at least one location was obtained each day.

### 2.3 Survival estimates

The survival outcome of each individual was estimated using the absolute difference in time between the dates of the last transmissions of the DSA and SPOT tags. Two discrete groups were identified. The first represents individuals whose tags ceased to transmit within a short time of one another (<30 hr), likely reflecting a mortality event. The second represents individuals with a large gap between the times of last tag transmissions (>528 hr/22 days; see Table S1 in Supporting Information S.1), reflecting either retrieval of the DSA tag on return to Kerguelen Islands or its possible failure (SPOT tags always out‐transmitted DSA tags, and were left attached to animals after return to Kerguelen Islands). While it is noted that there is a small chance both tags could have failed simultaneously (thus presenting a false mortality event), we consider the likelihood of this negligible, since within the surviving group, for all but one individual, tags continued to function until return to Kerguelen Islands (reflecting the completion of the first trip at sea following weaning and a successful survival outcome). For the surviving individual that did not return to Kerguelen (ID 140066; see Table S1 in Supporting Information S.1), the first of the two tags failed after 176 days at sea and the second 22 days later after a total of 198 days at sea.

### 2.4 Pre‐analytical data preparation

To investigate causes of mortality, for all individuals, we generated indices representative of (a) an individual's morphology and departure information, (b) horizontal movements, (c) fine‐scale acceleration‐ and dive‐based foraging behaviors, (d) changes in drift rate/body condition, and (e) encountered environmental conditions (Table 1).

**Table 1:** Indices used in analytical investigations of survival rates

Variable name	Description
(a) Morphology and departure information (one value per individual)	
Sex	Sex of individual (male/female)
Departure date	Date each individual commenced the outward phase of first trip at sea following weaning
Departure weight	Departure weight (kg) estimated following Guinet (1994) as weight_(_ *_t_*_)_ = weight_(_ *_t_*_−1)_ − (0.0048(weight_(_ *_t_*_−1)_) + 0.3031), where *t* is time in days from tag deployment to pup departure
Departure drift rate	Departure drift rate (m/s) taken as an indicator of departure condition
(b) Horizontal movement metrics (time series at day scale)	
Max speed	The daily maximum current corrected speed (m/s) between two hourly filtered locations
Distance swam	The total current corrected distance swam (km) across a 24‐hr period
Current deviation	The daily mean difference between corrected swimming direction and the direction of ocean currents (^o^). Current deviations were rescaled so as bearings 180–360 ran from 180 (against ocean currents) to 0 (with ocean currents), which is consistent with those of original bearing 0–180
(c) Acceleration and dive summaries (time series at dive scale)	
Dive depth	The maximum depth attained during a dive (m)
Dive duration	The total duration of a dive (s)
Scaled surface interval	The surface interval divided by the total duration of a dive (s)
PrCA rate	Total time spent in prey catch attempt (PrCA) behaviors (total time during which accelerations reflected “jerk”‐like movements) divided by total duration of a dive (s)
Scaled bottom duration	The total bottom duration divided by the total duration of a dive (s)
Swim effort	The total swimming effort (summed filtered accelerations associated with flipper movements across the lateral axis of the accelerometer) of the descent and ascent phases of a dive divided by the total corresponding durations of these phases (m/s^3^)
(d) Change in drift rate/body condition (time series at day scale)	
Drift rate change	Daily change in drift rate (m/s), between adjacent days. Positive values suggest an increase in seal buoyancy between days (likely through the acquisition of fat reserves) and negative values a decrease (either due to muscle development and/or loss of fat reserves)
(e) Environmental conditions (times series at day scale)	
Max wind	Daily maximum wind speed (m/s) encountered by an individual
Max wave height	Daily maximum wave height (m) encountered by an individual
Sea surface temperature	Daily mean surface temperature (°C) recorded by each individual's tag (at ~ 2‐3 m)
Max EKE	Daily maximum encountered eddy kinetic energy (EKE) taken as 0.5 ($u_{\text{current}}^{2}+v_{\text{current}}^{2}$)

For each individual, these are representative of (a) morphology and departure information (one value), (b) horizontal movements (estimated daily), (c) fine‐scale acceleration‐ and dive‐based foraging behaviors (estimated for each dive transmitted), (d) drift rates/body condition (estimated daily), and (e) encountered environmental conditions (estimated daily)

#### 2.4.1 Morphology and departure information

Differences in survival rates between females and males were investigated alongside the influence of departure date, weight, and condition. Departure dates were the day an individual commenced a continual trajectory away from Kerguelen Islands (~0–25 days after tag deployment; Supporting Information S.1). Departure weights were estimated from an individual's weight at tag deployment, following Guinet (1994) as weight_(_ *_t_*_)_ = weight_(_ *_t_*_−1)_ − (0.0048(weight_(_ *_t_*_−1)_) + 0.3031), where *t* is time in days from tag deployment to pup departure. Individuals that left Kerguelen on the same day as tag deployment had the same deployment and departure weights. Departure conditions were taken as extrapolated drift rates (departure drift rate; see details below under “Drift rates/body condition”).

#### 2.4.2 Horizontal movement metrics

Argos location data were not equally positioned in time and varied in quality (specified location class errors; http://www.argos-system.com). To obtain filtered location estimates at a temporal resolution of 1 hr, all data were processed using a hierarchical first difference correlated random walk state‐space model (SSM; Jonsen, Flemming, & Myers, 2005; Supporting Information S.2). At‐sea horizontal swimming behaviors were then corrected to remove trajectory distortion caused by ocean currents (Gaspar et al., 2006; Supporting Information S.2). Following this, for each individual, the daily maximum corrected speeds observed across a single hourly period were extracted alongside the total corrected distances traveled in a day. The first and last days of each track were excluded to ensure all days encompassed a full 24 hr of tracking data and were thus comparable. To assess differences between how individuals orientate themselves to ocean currents (i.e., with or against), the daily circular mean deviation of a pup's real heading from the concurrent ocean current direction was calculated. These values were rescaled so as bearings 180–360 ran from 180 (against ocean currents) to 0 (with ocean currents), which is consistent with those of original bearing 0–180.

#### 2.4.3 Transmitted acceleration and dive summaries

For each dive performed by each individual, the maximum dive depth was taken alongside the total dive duration. Surface intervals were scaled by dive duration, as were times spent in PrCA behaviors. Descent, bottom, and ascent dive phases were identified following Cox et al. (2018). For each dive performed by each individual, the bottom duration scaled by the total dive duration was taken. In addition, the average swimming effort required to transit to and from the bottom, hunting phase, of a dive was estimated as the sum of the swimming efforts of descent and ascent phases divided by the total duration of these phases.

#### 2.4.4 Drift rates/body condition

Southern elephant seals regularly perform resting dives, where individuals cease active movement and “drift” in the water column (Biuw, McConnell, Bradshaw, & Fedak, 2003; Gordine, Fedak, & Boehme, 2015; Mitani et al., 2010). Vertical movement during these periods can be used to make inferences on an individual's buoyancy, and thus body composition and condition (i.e., ratio of high‐density lean to low‐density lipid tissue; Biuw et al., 2003). Animals that are positively buoyant should be in “better” condition than those that are negatively buoyant (due to increased fat reserves). During their first trip at sea, juvenile southern elephant seals are still developing and growing, and as such, changes in buoyancy could also reflect the acquisition of lean muscle in addition to changes in fat reserves (Biuw et al., 2003; Orgeret, Cox, Weimerskirch, & Guinet, 2019). To identify drift dives, we used a modified stepwise filtering process similar to that described in Biuw et al., (2003) and Gordine et al., (2015), but that also took into account information from accelerometer transmissions (Supporting Information S.3). Vertical movement rates in each drift segment were then calculated. Following this, to obtain daily drift rate estimates (as drift dives are not always performed daily), we used functional data analysis (Yang, Zhu, Choi, & Cox, 2016; Supporting Information S.3). Daily changes in drift rate were then estimated.

#### 2.4.5 Environmental conditions

To investigate the impact of storms on juvenile survival, wind and wave data were extracted from the ERA‐Interim global atmospheric reanalysis (Dee et al., 2011), via the European Centre for Medium‐Range Weather Forecasting (ECMWF; http://www.ecmwf.int/datasets), at a spatiotemporal resolution of 0.75° and 3 hr. For each individual, the daily maximum of the closest spatio‐temporally matched wind and wave conditions was extracted.

Information on fine‐scale biophysical habitats encountered by each individual, which may impact prey availability/quality (Abrahms et al., 2018; Richard, Cox, Picard, Vacquie‐Garcia, & Guinet, 2016), was also obtained. For each individual for each day, SST was taken from the DSA tag as the mean temperature of all measurements of that day. Current data were taken from the delayed time all‐sat‐merged Global Ocean Gridded Absolute Geostrophic Velocities Anomalies L4 product of AVISO (http://www.aviso.altimetry.fr), on a daily basis at a spatial resolution of 0.25°. An index of total (eddy) kinetic energy (m^2^/s^2^; TKE) was then calculated as 0.5 ($u_{\text{current}}^{2}+v_{\text{current}}^{2}$). The maximum spatio‐temporally matched values encountered by each individual per day were extracted.

### 2.5 Data analyses

Due to a limited sample size and high level of noise/variability in the data (i.e., individuals may die for a range of reasons), investigations toward causes of early mortality were predominantly exploratory in nature. Prior to all analyses, we verified that sampling bias (i.e., variation in the number of dives transmitted by the DSA devices through time and between individuals) was not an issue (Supporting Information S.4). To ensure comparability, overlap in the broad‐scale spatial distributions of surviving and non‐surviving groups was also assessed using kernel density analysis and Bhattacharyya's affinity (Fieberg and Kochanny 2005; Supporting Information S.5).

#### 2.5.1 Temporal patterns in survival

Kaplan–Meier survival functions were generated using the survival package in R (Therneau & Lumley, 2017), and used to assess the distribution and probability of mortality events with time since an individual left Kerguelen Islands. For non‐survivors, the last date at which both tags were transmitting was used as the date of death. For surviving individuals, time‐series data were right‐censored to the date at which an individual returned to Kerguelen Islands. For one surviving individual that did not return to Kerguelen Islands and instead spent an extended time period within shelf waters around Heard Island, a censoring date corresponding to the average return date to Kerguelen Islands of the other survivors was used.

#### 2.5.2 Individual comparisons between non‐surviving pups and grouped survivors

For each day, for each predictor for which time‐series data were available (Table 1), daily estimates for each non‐surviving individual were plotted against the daily medians of the entire surviving group, alongside the 2.5%, 25%, 75%, and 97.5% quantiles. This allowed us to visually assess behavioral patterns and environmental conditions encountered in relation to time since departure (and ontogenetic processes), and the time of death (i.e., were behaviors consistently different between surviving and non‐surviving individuals, or did a change occur in the days and/or weeks immediately prior to death). For predictors not already generated at a day scale (i.e., that were generated at the dive scale; Table 1), daily summaries were calculated for use only in these visual assessments (i.e., maximum daily dive depth, maximum daily dive duration, daily mean scaled surface interval, daily mean PrCA rate, daily mean scaled bottom duration, and daily mean swim effort).

#### 2.5.3 Statistical assessment of potential common causes of mortality across the sampled population

To assess common causes of mortality across the entire sampled population, a two‐step modeling approach was applied, similar to that of joint distribution modeling frameworks (e.g., Henderson, Diggle, & Dobson, 2000), albeit modified so a range of parameter estimation techniques could be applied. First, for each potential predictor for which time‐series data were available (at either a daily or dive scale; Table 1), univariate linear mixed‐effects models (LMMs) were used to extract the intercept and slope of each predictor modeled against time since departure from Kerguelen Islands for each individual. These were fitted as random intercept–slope models via the nlme package in R (Pinheiro & Bates, 2014). The time variable (days since departure) was standardized such that the intercept would represent the average of a potential predictor across the series, and the slope, its change through time. Parameter estimates for each individual were then extracted as the corresponding intercept and slope from the random component of the model with the population‐level coefficient (intercept or slope) subtracted (to standardize the data). Because all individuals that died did so either during their outward trip phase or very soon after, to ensure temporal comparability, all data from surviving individuals were filtered so that only samples taken during outward trip phases (i.e., until a trip's distal point) were included in analyses.

Due to the noisy and high‐dimensional nature of our study (i.e., small sample size and large number of potential predictors), a conservative approach using three different methods was used to assess how each of the summarized time series influenced survival (alongside sex and departure date, weight, and drift rate; Table 1). Predictors were selected when at least two methods agreed on importance. First, Cox proportional hazards (ph) regression models (Cox, 1972) were fitted via the survival package in R (Therneau & Lumley, 2017). Cox ph models are non‐parametric and assess how candidate predictors influence the hazard rate of a particular event happening (here death). A positive estimate for a predictor reflects an increased hazard rate, and thus negative influence on survival. Models were fitted for each candidate predictor one at a time (Table 1). The response variable (survival) was specified as described in the generation of Kaplan–Meier survival functions above. Significance against a null model was tested for using one‐way analysis of variance at *p* < .05. The proportionality assumption was checked using the Schoenfeld residuals test (Grambsch & Therneau, 1994).

The second and third methods considered all candidate variables in tandem and selected key predictors using “shrinkage” techniques, which are both well suited to high‐dimensional studies and can be tuned to deal with collinearity (Pavlou, Ambler, Seaman, Iorio, & Omar, 2016; Tibshirani, 1997; Zou & Hastie, 2005). Such methods work by shrinking all coefficients toward zero and adding a penalty on their size, such that only the stronger signals retain non‐zero coefficients. The first of these was implemented using the glmnet package in R (Friedman, Hastie, Simon, Qian, & Tibshirani, 2017), which fits a Cox ph model regularized by an elastic net penalty (Friedman, Hastie, & Tibshirani, 2010; Simon, Friedman, Hastie, & Tibshirani, 2011). An optimal shrinkage penalty of 0.317 was selected by eight‐folds cross‐validation (Simon et al., 2011).

The second shrinkage‐based analysis (and third applied method) used a regularized horseshoe prior (Piironen & Vehtari, 2017) and was implemented in Stan (Carpenter et al., 2017; Supporting Information S.6). The advantage of the regularized horseshoe is to have explicit control on the prior for the number of non‐null predictor variables, which was set to 10 (based on the outputs of the two previous investigations). Survival time was modeled as a normal distribution (on a log scale), which corresponds to a non‐monotonic hazard. Such a structure is appropriate since after leaving Kerguelen Islands, mortality hazard can be expected to increase after weaning (as energy capital from maternal provisioning depletes), and then decrease as pups learn to forage (Orgeret et al., 2019). To assess true and false predictor detection rates of the model of this third method (and determine its suitability to our dataset), a retrospective power analysis was also performed (Supporting Information S.7).

## 3 RESULTS

**Figure 1:**
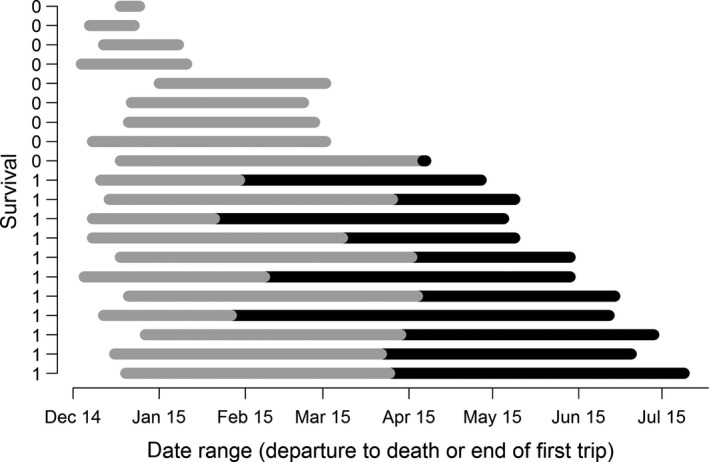
Individual tracking ranges from departure from Kerguelen Islands to either time of mortality or return to Kerguelen Islands (gray bar components are outward trip phases and black return). Note only one non‐survivor commenced a distinct return trip phase, which lasted 49 days (1,084.4 km) before the individual died

**Figure 2:**
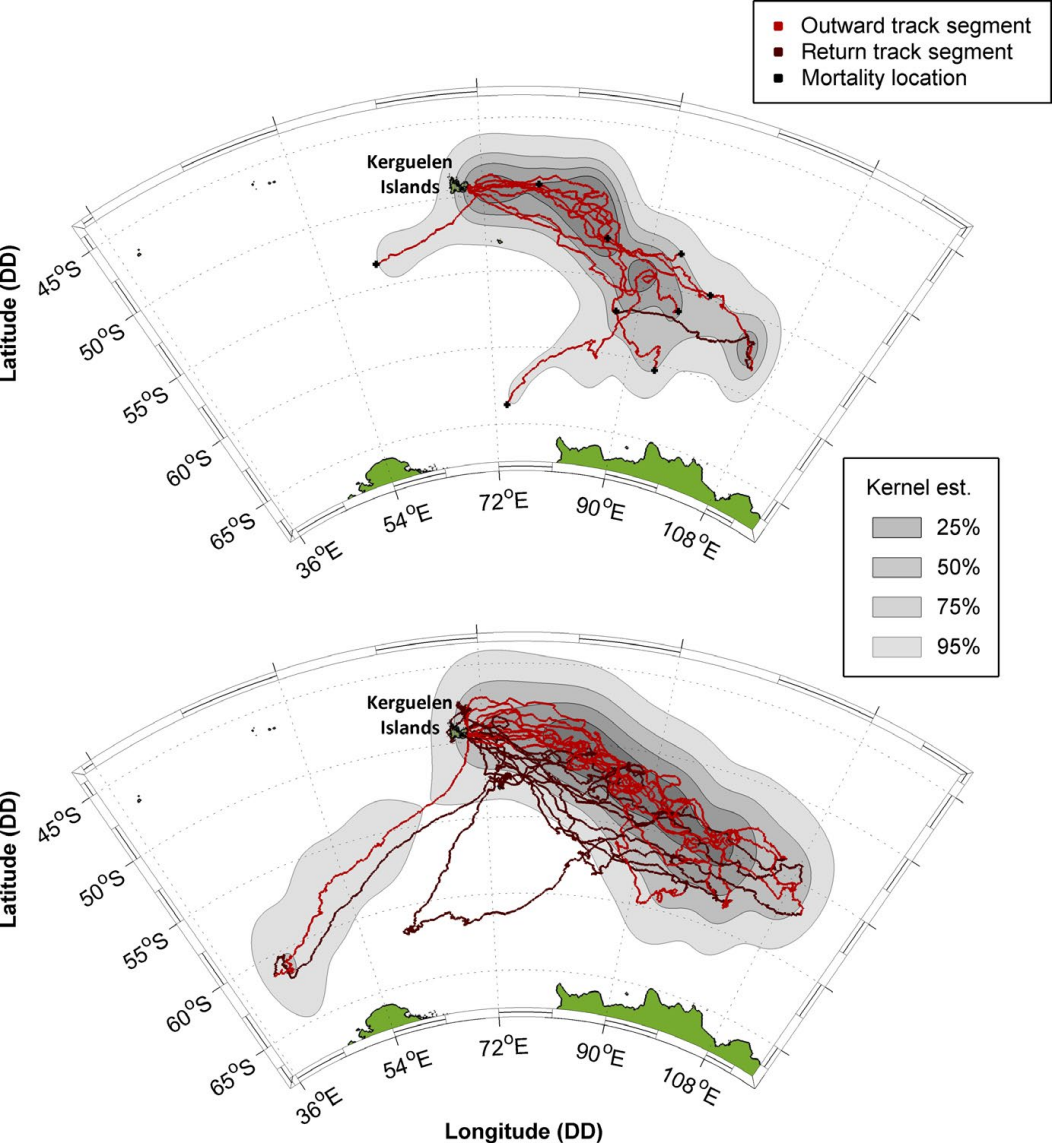
Spatial distributions of (a) non‐survivors and (b) survivors. Lighter red track portions represent outward trip phases (until death or distal location) and dark red return phases. Graduated gray polygons show kernel densities at the 25%, 50%, 75%, and 95% levels, using, for non‐survivors (a), all filtered state‐space model (SSM) locations and, for survivors (b), filtered SSM locations from outward trip phases only (Supporting Information S.5). In plot (a), black markers indicate mortality events

Of the 20 tracked pups, nine died (two females and seven males) and 11 survived (eight females and three males) until at least the end of their first trip at sea following weaning (Figure 1, Supporting Information S.1, Table S1). All individuals bar two (one survivor and one non‐survivor) headed in a southeastward direction upon departing Kerguelen Islands (Figure 2). No broad‐scale spatial segregation was apparent between surviving and non‐surviving pups (Figure 2 and Supporting Information S.5).

### 3.1 Temporal patterns in survival

**Figure 3:**
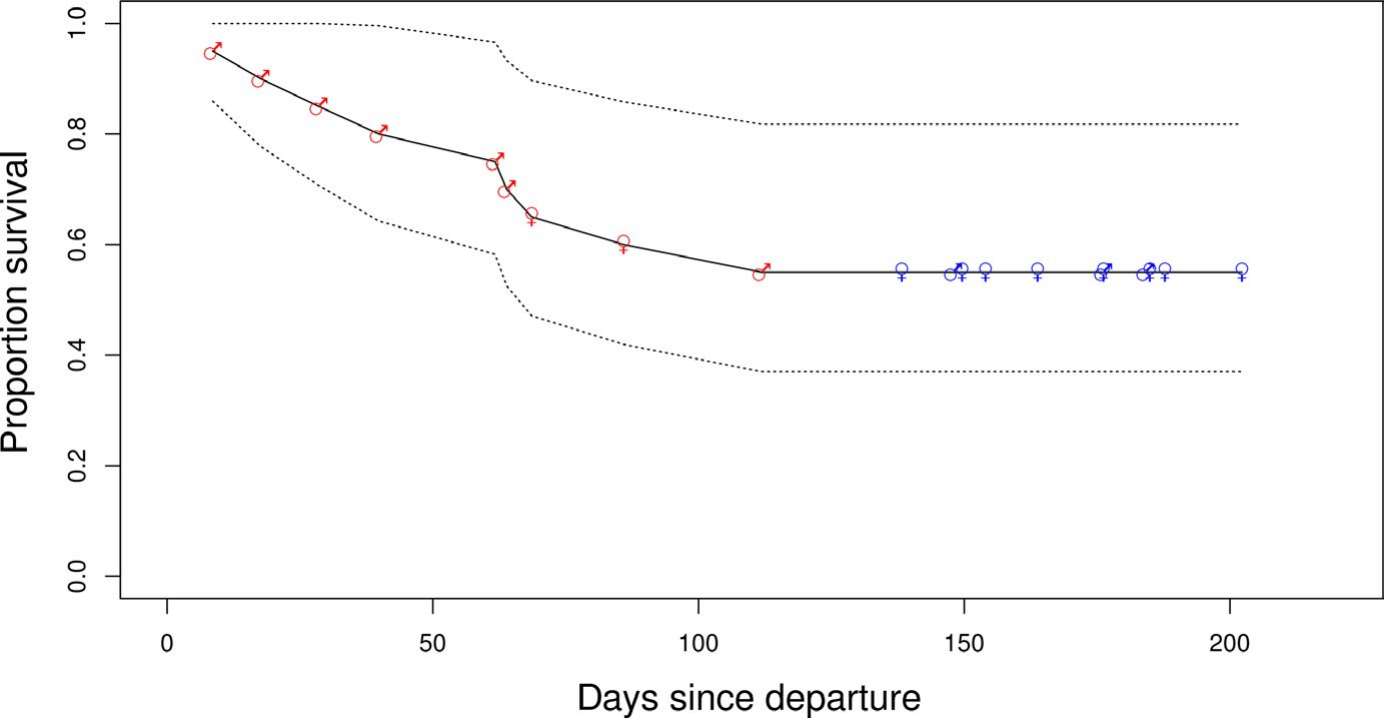
Kaplan–Meier survival functions. Red markers indicate mortality events (deaths) and blue markers censoring (i.e., return to Kerguelen Islands; ♂ = males and ♀ = females)

All mortality events occurred between 8.6 and 112 days after departure from Kerguelen Islands (mean = 54 ± 33.5 days, ±standard deviation), at a straight‐line distance of between 521 and 2,468 km from Kerguelen Islands (mean = 1,534.3 ± 604.4 km; Figures 1 and 3). For all but two individuals, this happened before (i.e., at) the distal point of a trip (Figures 1 and 2). For the two individuals that commenced return trip phases, death occurred 0.2 (1.7 km) and 49 days (1,084.4 km) after reaching their recorded distal points (which, for the first of these two individuals, may not represent the true distal location of the trip had it continued). Of the surviving individuals, return arrival at Kerguelen Islands occurred between 138.2 and 202.2 days after departure (mean = 169.5 ± 20.14 days), between April and July (Figure 1). The distal points of these trips occurred between 44 and 105 days after departure (mean = 81.2 ± 24.4 days) at straight‐line distances of between 1,131.0 and 2,771.6 km from Kerguelen Islands (mean = 2,036.9 ± 534.4 km). Overall, weanling survival rates during their first trip at sea were 55% (males 30% and females 70%).

### 3.2 Individual comparisons between non‐surviving pups and grouped survivors

Visual comparisons of behaviors and encountered environmental conditions of non‐surviving pups to those of grouped survivors suggested both marked differences (at times) and high heterogeneity (Table 2; see Supporting Information S.8 for all plots used in these assessments).

**Table 2:** Individual by individual visual comparisons for each non‐survivor against grouped survivors (see plots in Supporting Information S.8), alongside life span length and departure weights and drift rates

	140061	140064	140065	140067	140070	140071	140074	140076	140078
Days since departure at death	64	40	18	86	9	112	29	62	69
Sex	M	M	M	F	M	M	M	M	F
Departure weight (kg)	83.7	73.4	82.0	59.4	89.3	83.0	44.6	96.1	67.5
Departure drift rate (m/s)	0.17	−0.19	0.18	0.18	0.17	0.12	0.14	0.10	0.19
Max speed	—	—	—	—	—	—	Upper range	Rise at end	—
Distance swam	—	—	Upper range	—	—	Peak and drop at end	Upper range	Rise at end	—
Current deviation	—	—	—	—	—	—	—	—	—
Dive depth	—	—	—	—	Upper range	Distinct peak at end with severe drop‐off	Distinctly lower	Increased at beginning	Increased at end
Dive duration	—	—	Upper range	—	Upper range	Distinct peak at end with severe drop‐off	Distinctly lower	Increased at beginning	Lower range except during one day a few days prior to death
Scaled surface interval	—	Slightly higher at end	—	—	—	Extreme positive value prior to death	Distinctly higher	—	Several increased periods
PrCA rate	Long period in lower range	—	—	Upper range	—	Drop off on the last day	Distinctly decreased	Decreased at end	Decreased at start
Scaled bottom duration	—	—	—	—	—	—	Decreased	—	Slightly decreased at end
Swim effort	Lower range	Lower range	Upper range	Upper range ‐more so at end	Lower range	Upper range at start. Small peak and sharp drop off at end	Distinctly increased	Slight increase at end	Upper range
Drift rate	—	—	—	—	—	—	—	—	—
Drift rate change	—	—	—	—	—	—	—	—	—
Max wind	—	—	—	—	—	—	—	—	—
Max wave height	—	—	—	—	—	—	—	—	—
Sea surface temperature	Lower range	Lower range at start	Lower range	Higher range	—	—	Lower range	Lower range at end	Lower range
Max EKE	Lower range at end	—	Lower range	—	—	Lower range at end	—	Lower range at end	—

Note

Blank entries (“—”) indicate no discernible difference in behavior.

**Figure 4:**
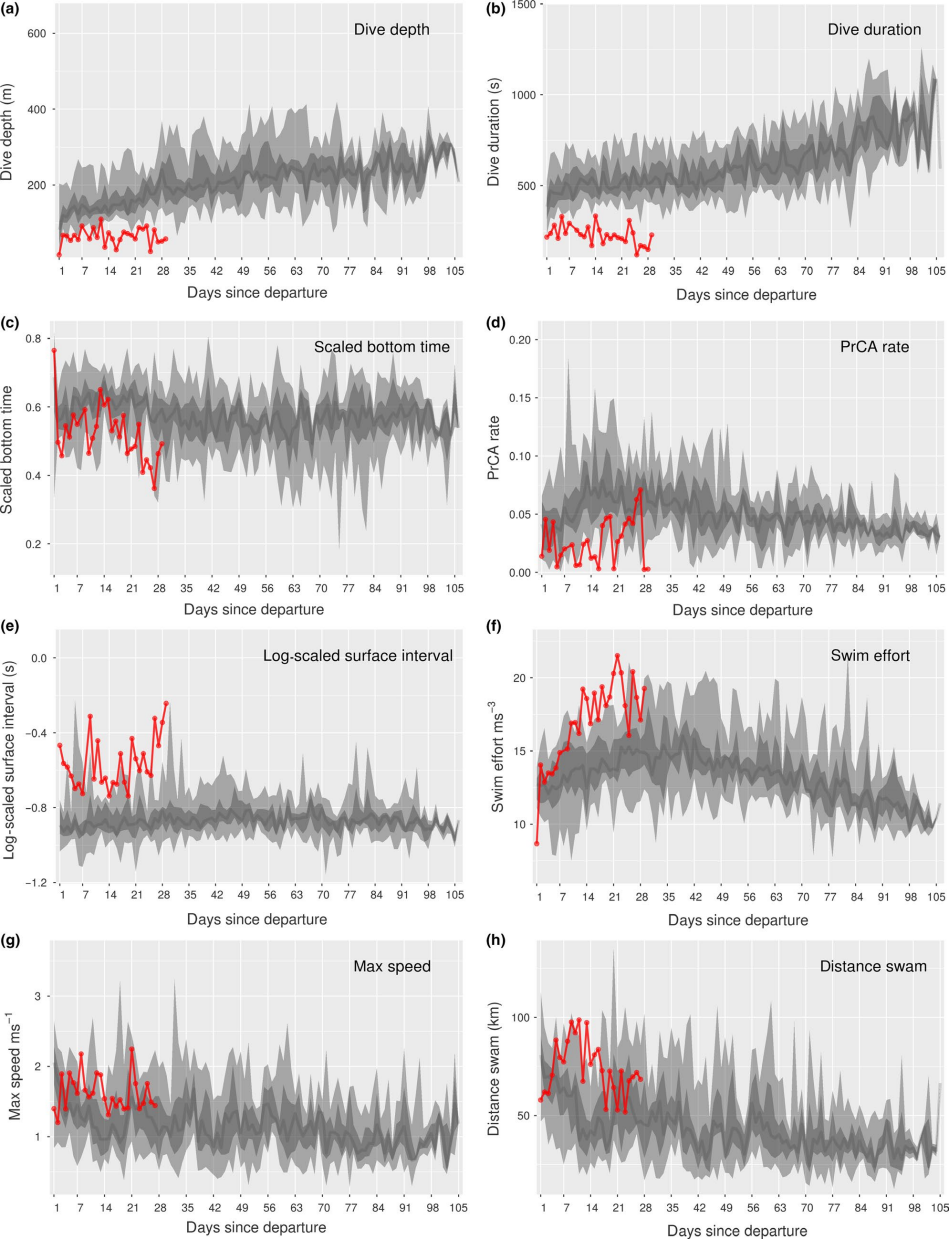
Time series behaviors for individual 140074. Daily values (red) for (a) maximum dive depths, (b) maximum dive durations, (c) mean scaled bottom times, (d) mean prey catch attempt (PrCA) rates, (e) mean log‐scaled surface intervals, (f) mean swim efforts, (g) maximum speeds, and (h) total distances swam. Large light gray bands represent concurrent 2.5%–97.5% quantiles of survival datasets, nested dark gray bands the 25%–75% quantiles, and dark gray lines the median

**Figure 5:**
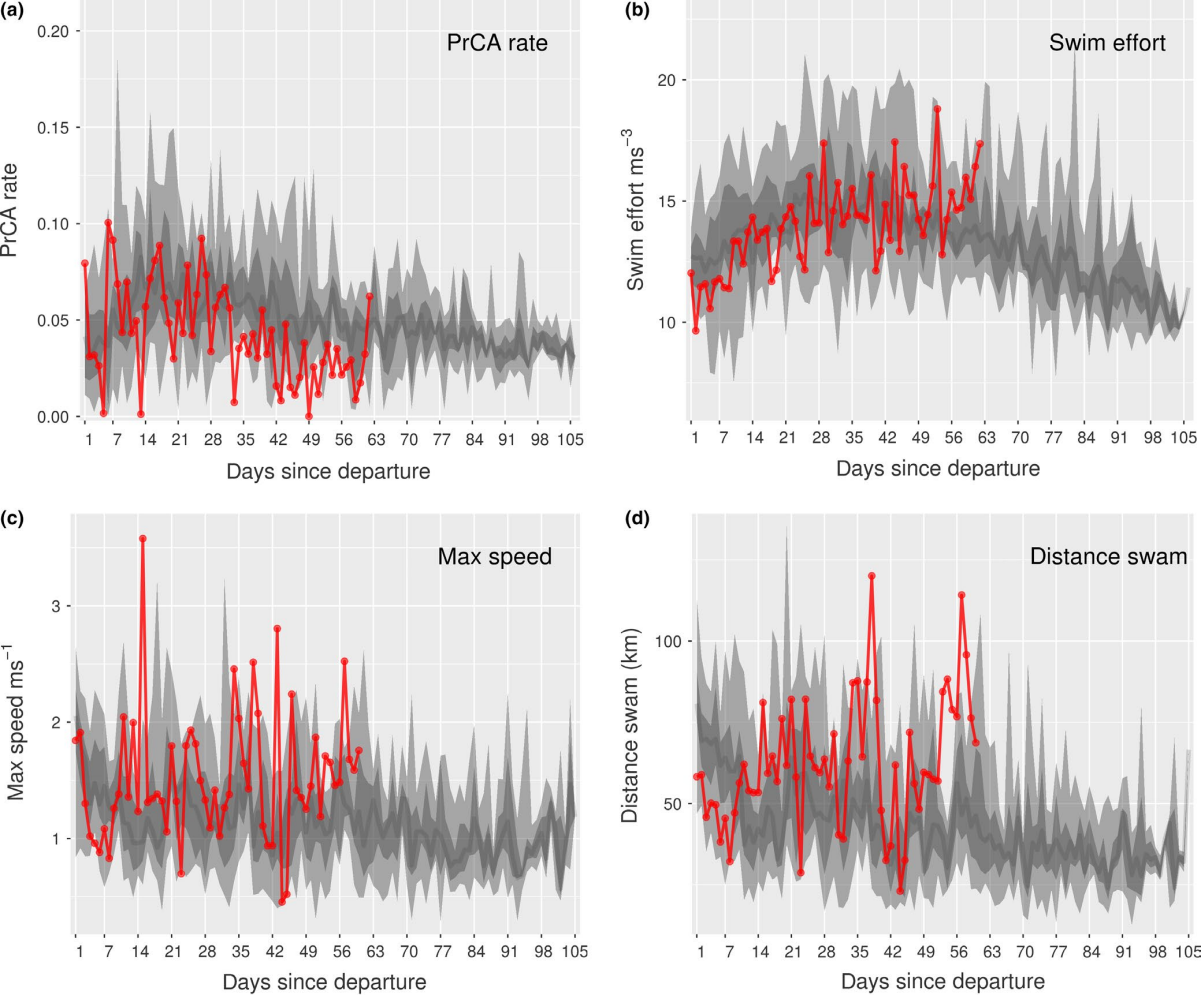
Time series behaviors for individual 140076. Daily values (red) for (a) mean prey catch attempt (PrCA) rates, (b) mean swim efforts, (c) maximum speeds, and (d) total distances swam. Large light gray bands represent concurrent 2.5%–97.5% quantiles of survival datasets, nested dark gray bands the 25%–75% quantiles, and dark gray lines the median

**Figure 6:**
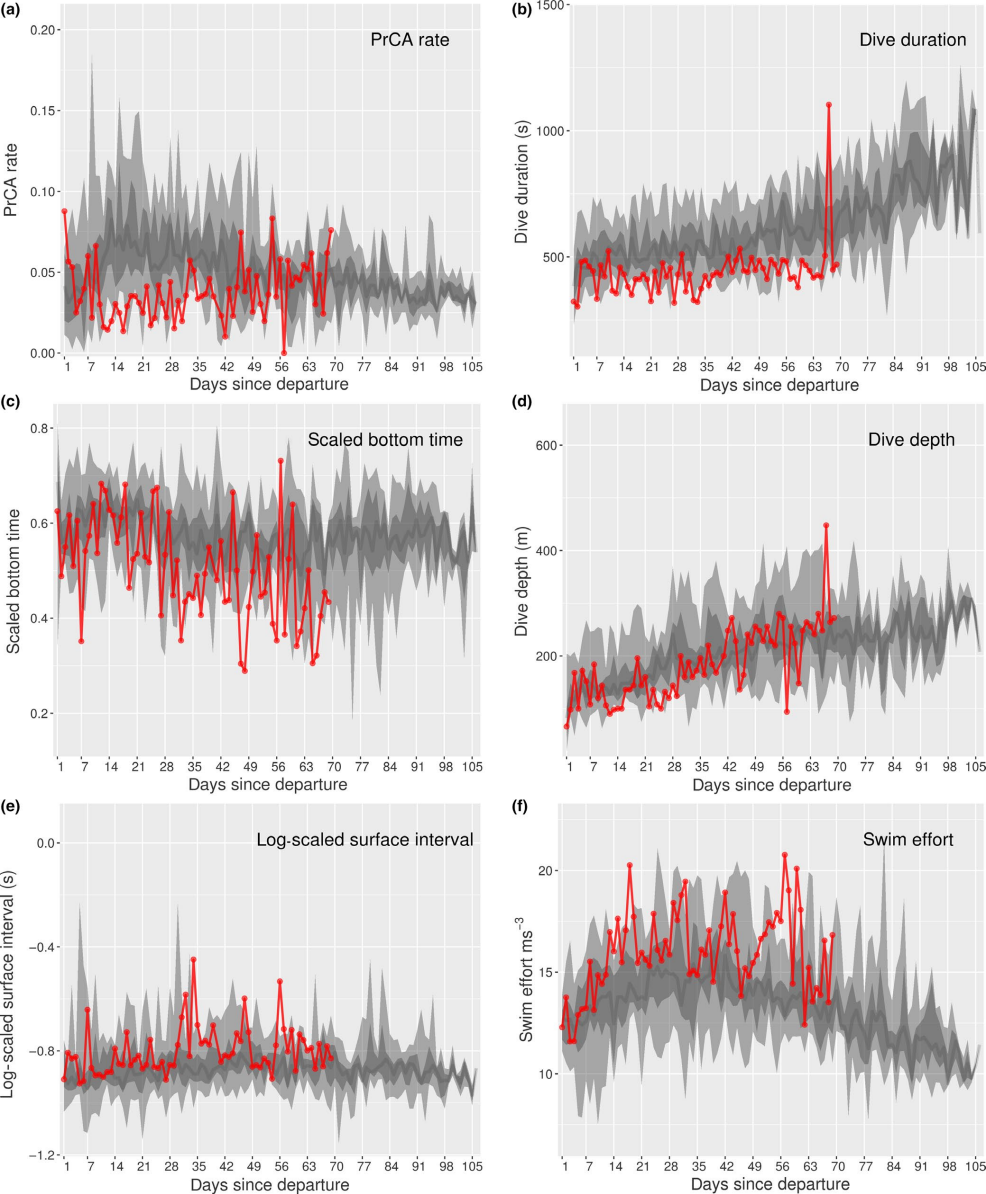
Time series behaviors for individual 140078. Daily values (red) for (a) mean prey catch attempt (PrCA) rates, (b) maximum dive durations, (c) mean scaled bottom times, (d) maximum dive depths, (e) mean log‐scaled surface intervals, and (f) mean swim efforts. Large light gray bands represent concurrent 2.5%–97.5% quantiles of survival datasets, nested dark gray bands the 25%–75% quantiles, and dark gray lines the median

For four non‐survivors (IDs: 140061, 140074, 140076, and 140078), periods of decreased dive and forage ability were noted. This was particularly prominent for the lightest individual at departure (pup 140074; Table 2). Specifically, maximum daily dive depths and durations alongside mean bottom times and PrCA rates were low (Figure 4). This was accompanied by increased daily mean surface intervals and swimming efforts, alongside total daily distances traveled and maximum swim speeds (Figure 4). For pups 140061, 140076, and 140078, while behavioral indices indicative of dive and forage ability were generally consistent with those of the surviving group, prolonged periods with decreased PrCA rates were observed (Table 2, Figures 5 and 6). For individual 140076, this was most prominent in the weeks immediately prior to death and was accompanied by an increase in daily travel distances, maximum swim speeds, and daily mean swimming efforts (Figure 5). For individual 140078, maximum daily dive durations were in the lower range of that observed in surviving pups (Figure 6). Moreover, in the weeks immediately prior to death, there was a decrease in bottom duration alongside a slight increase in daily maximum dive depths. In addition, surface intervals were sometimes increased as were daily mean swimming efforts (Figure 6).

**Figure 7:**
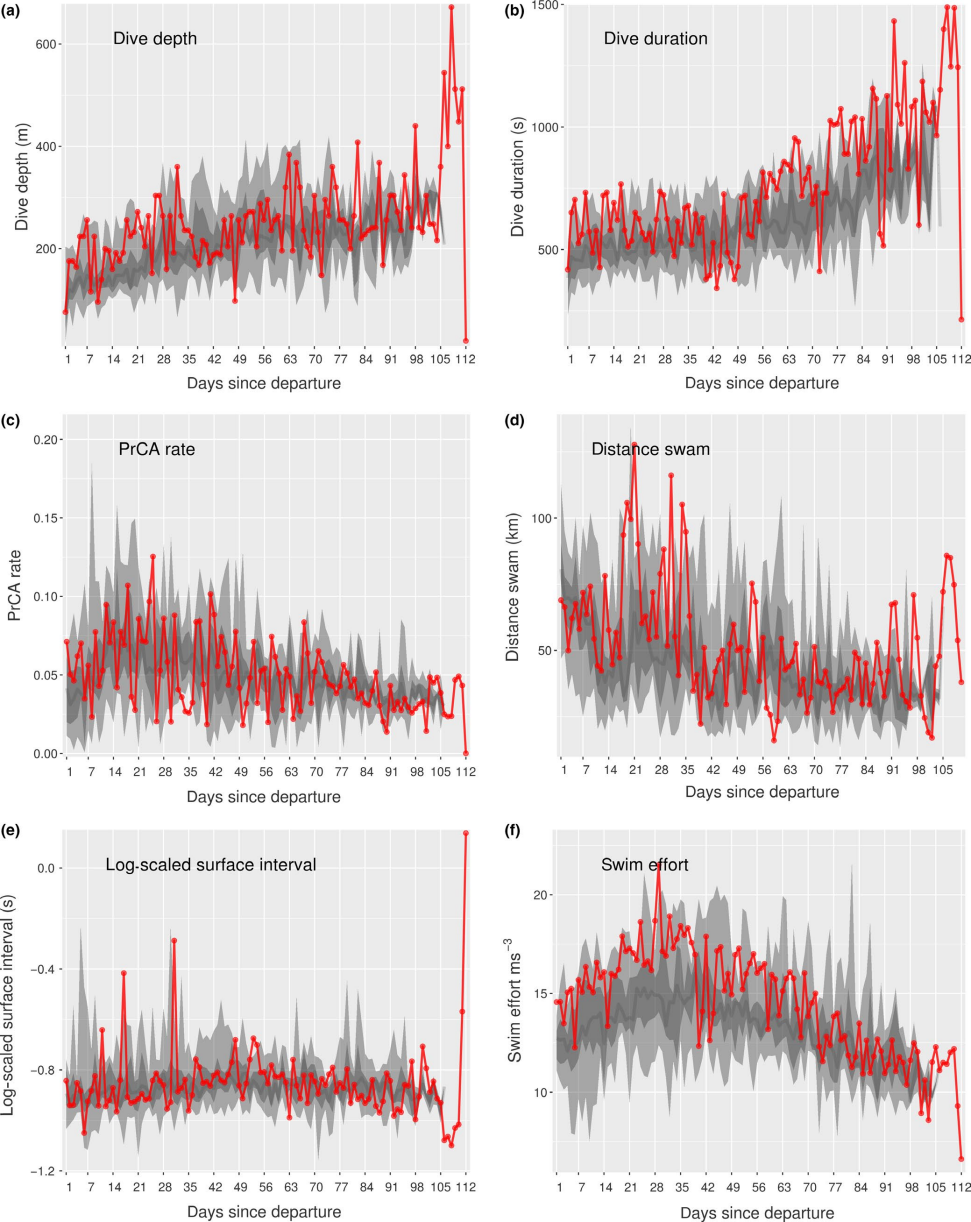
Time series behaviors for individual 140071. Daily values (red) for (a) maximum dive depths, (b) maximum dive durations, (c) mean prey catch attempt (PrCA) rates, (d) total distances swam, (e) mean log‐scaled surface intervals, and (f) mean swim efforts. Large light gray bands represent concurrent 2.5%–97.5% quantiles of survival datasets, nested dark gray bands the 25%–75% quantiles, and dark gray lines the median

For individual 140071, while persistent differences in dive and forage ability to the surviving group were not present, the days immediately prior to death saw a distinct behavioral change (Table 2, Figure 7). This included a sharp peak in maximum daily dive depths and durations, which were substantially increased compared to those both performed previously and of the surviving group at comparative times in development. These were accompanied by extreme surface intervals followed by a distinct drop‐off in daily mean PrCA rates and swimming efforts (Figure 7).

**Figure 8:**
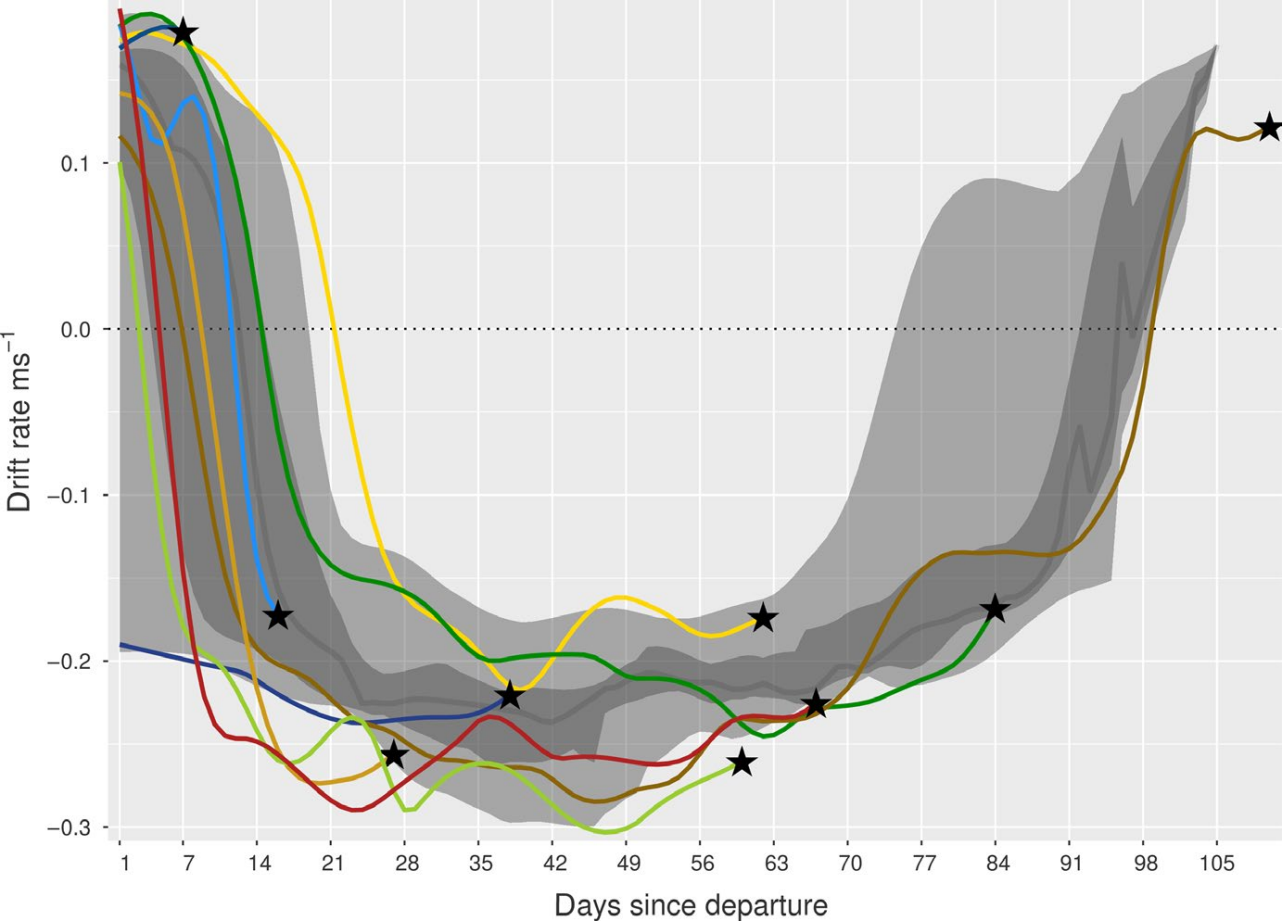
Drift rates of non‐survivors (colors correspond to individuals), with times of death (and corresponding drift rates) indicated by black stars. The large light gray band represents the 2.5%–97.5% quantiles of the survival dataset, the nested dark gray band the 25%–75% quantiles, and the dark gray line the daily median

While distinct differences in drift rates were not evident (suggesting no mean difference in body condition between survivors and non‐survivors), all individuals bar two (IDs: 140070 and 140071) died when negatively buoyant (indicative of decreased body condition; Figure 8). For pup 140070 (positively buoyant at death), mortality occurred within the first 2 weeks at sea, when the majority of individuals were positively buoyant following weaning.

**Figure 9:**
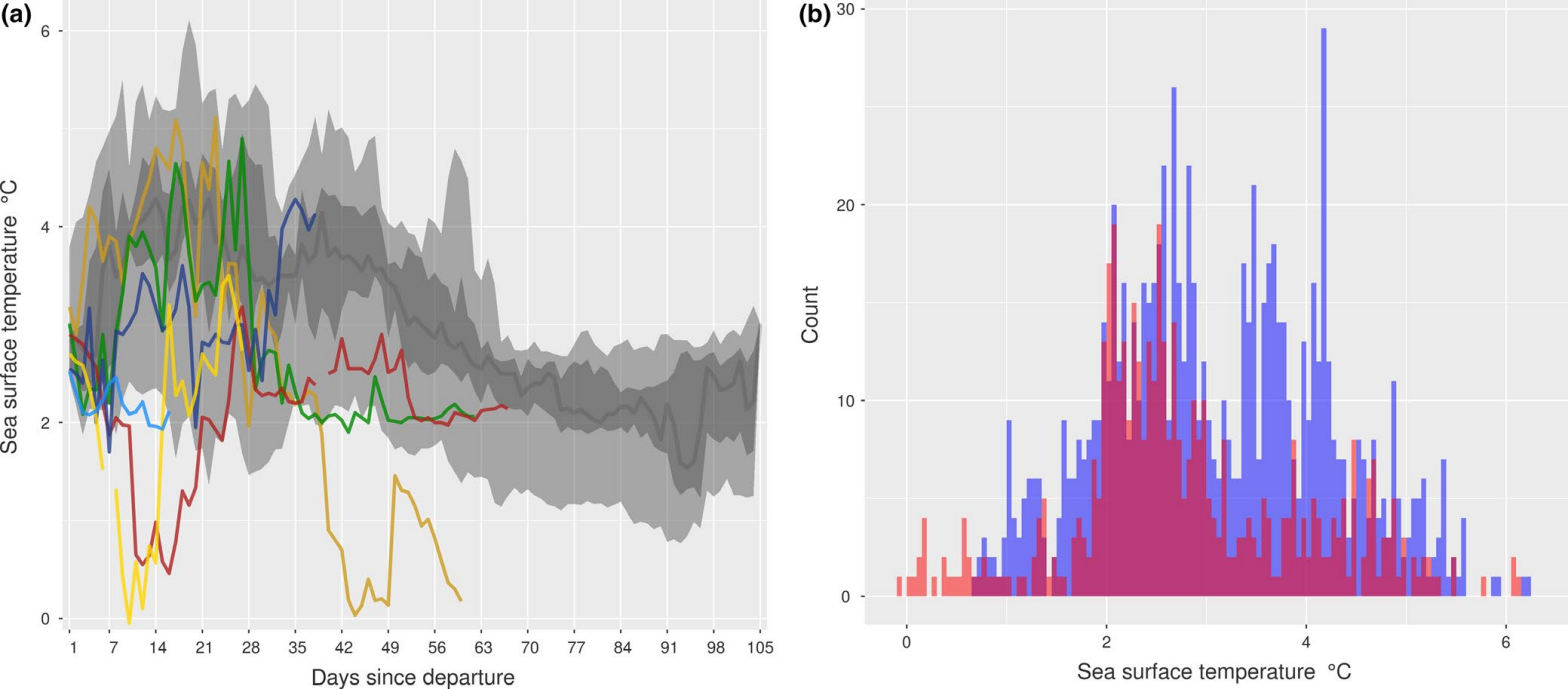
Sea surface temperatures (SSTs) of survivors and non‐survivors. Plot (a), time series for six individuals (140061, 140064, 140065, 140074, 140076, and 140078) that appear to visit waters of reduced SSTs. Here, the large light gray band represents the 2.5%–97.5% quantiles of the survival dataset, the nested dark gray band the 25%–75% quantiles, and the dark gray line the daily median. Individual plots for each pup are in Supporting Information S.8. Plot (b), histogram of SSTs of non‐survivors (red) and survivors (blue; outward trip phase only)

Finally, six of the nine pups that died foraged predominantly in waters cooler than those generally visited by surviving individuals (Table 2, Figure 9). For three of these individuals (and an additional one that remained in waters of SSTs similar to those visited by survivors), maximum TKE values were also reduced (Supporting Information S.9).

### 3.3 Statistical analyses of common causes of mortality across the sampled population

Eight predictors of survival outcome were selected by at least two of the three implemented methods (Table 3). In all instances, magnitude and sign agreement was good. Variable selection cutoffs for the horseshoe analysis were a parameter estimate of at least 0.05 and a shrinkage less than 0.855 (further details in Supporting Information S.10). Results from the retrospective power analysis performed on this method suggest the likelihood of a selected predictor reflecting a true signal to be 50% (Supporting Information S.7).

**Table 3:** Common causes of mortality as identified via the three modeling frameworks

Variable name	LMM estimate	COX ph estimate	COX ph *p*‐value	Elastic net estimate	Horseshoe estimate	Horseshoe shrinkage
Sex***	—	**1.76 (M)**	**0.015**	**0.21 (M)**	**0.12 (M)**	**0.81**
Departure weight	—	0.32	0.40	—	0.05	0.85
Departure date	—	0.09	0.79	—	0.00	0.87
Departure drift rate	—	0.16	0.68	—	0.01	0.87
Max speed						
Intercept***	**1.29 (0.12)**	**1.38**	**0.003**	**0.11**	**0.08**	**0.85**
Slope	−0.10 (0.099)	0.43	0.23	—	0.02	0.88
Distance swam						
Intercept***	**51.30 (7.45)**	**1.28**	**0.002**	**0.26**	**0.10**	**0.83**
Slope	−5.98 (5.09)	0.48	0.16	—	0.05	0.87
Current deviation						
Intercept	91.58 (10.75)	−0.19	0.64	—	−0.01	0.90
Slope	5.31 (11.12)	−0.29	0.51	—	−0.03	0.89
Dive depth						
Intercept	135.06 (29.93)	−0.50	0.16	—	−0.02	0.86
Slope	20.60 (13.54)	−0.62	0.11	—	−0.03	0.87
Dive duration						
Intercept	447.43 (86.26)	−0.32	0.39	—	−0.00	0.87
Slope**	**35.21 (34.98)**	**−0.87**	**0.03**	**−0.06**	**−0.04**	**0.86**
Scaled bottom duration						
Intercept	0.57 (0.03)	−0.17	0.64	—	−0.00	0.88
Slope	−0.02 (0.02)	−0.37	0.33	—	−0.02	0.88
PrCA rate						
Intercept	0.05 (0.01)	−0.76	0.07	−0.03	−0.05	0.86
Slope**	**−0.002 (0.007)**	**0.74**	**0.11**	**0.05**	**0.10**	**0.83**
Scaled surface interval						
Intercept	0.15 (0.03)	0.36	0.36	—	−0.00	0.87
Slope	0.003 (0.005)	0.66	0.13	—	0.01	0.90
Swim effort						
Intercept	14.00 (1.64)	0.13	0.73	—	0.00	0.87
Slope**	**0.01 (0.99)**	**0.70**	**0.06**	**0.07**	**0.05**	**0.85**
Drift rate change						
Intercept	−0.005 (0.006)	−0.85	0.03	—	−0.02	0.87
Slope	0.006 (0.005)	−0.55	0.18	—	−0.05	0.86
Max wind						
Intercept	14.51 (0.50)	−0.13	0.73	—	0.01	0.89
Slope	−0.09 (0.35)	−0.39	0.33	—	−0.01	0.90
Max wave height						
Intercept	4.63 (0.19)	−0.12	0.80	—	−0.00	0.91
Slope	NA	NA	NA	NA	NA	NA
Sea surface temperature						
Intercept**	**2.99 (0.71)**	**−0.57**	**0.07**	**−0.10**	**−0.06**	**0.85**
Slope	−0.29 (0.47)	0.36	0.35	—	0.05	0.86
Max EKE						
Intercept**	**0.05 (0.02)**	**−0.48**	**0.15**	**−0.08**	**−0.08**	**0.85**
Slope	−0.003 (0.02)	0.11	0.75	—	0.02	0.88

For each potential predictor of survival: (a) coefficient estimates from linear mixed‐effects models (LMMs; standard deviation around random effects in brackets), (b) Cox ph estimates, (c) p‐values from analysis of variance on Cox ph tests, (d) elastic net parameter estimates, (e) parameter estimates from horseshoe analyses, and (f) proportion of shrinkage applied to each horseshoe parameter estimate. Note that for Cox ph, elastic net and horseshoe estimates, a positive value means a decrease in survival with increasing LMM coefficient. Variables selected by two (**) or thee (***) multiple methods are highlighted in bold with selected values underlined.

Male survival was lower than female survival. Survival decreased with increased daily maximum speeds and distances traveled. These results were consistent across all three methods (Table 3). A positive influence of an increase in dive duration through time on survival was identified by the Cox ph and elastic net analyses, but was one of the weaker predictors from the horseshoe analysis (relatively small parameter estimate and high shrinkage; Table 3). The four other predictors deemed to impact survival were identified by both the elastic net and horseshoe analyses, but yielded *p*‐values from the Cox ph analysis that were above the .05 cutoff (but note that these were all between .06 and .15). There was an influence of a decrease through time in PrCA rates, such that a greater decrease increased survival probability. Individuals with swim efforts that increased through time were more likely to die than those whose swim efforts did not (or did so less). Finally, individuals that typically visited waters characterized by increased SSTs and TKE estimates were more likely to survive than those visiting waters with decreased estimates.

## 4 DISCUSSION

In this study, we capitalized on new techniques in the abstraction and transmission of accelerometer and dive data, and coupled these to double tagging methods to investigate and gain a more mechanistic understanding of how the fine‐scale behaviors of juvenile southern elephant seals impact survival outcome during their first trip at sea following weaning. Using a cutting‐edge yet conservative analytical approach, we provide novel insight toward the causes of early mortality in this far‐ranging marine predator, which we suggest results from a mix of reduced dive and forage ability alongside habitat quality and possible predation. This is one of few studies to use bio‐logging technologies (and particularly accelerometers) to assess the factors impacting survival in a long‐lived marine vertebrate predator, and the methods we employ have the potential to be applied across a number of other taxa to gain novel insight of the drivers of individual mortality and population dynamics.

### 4.1 Overall survival rates and sex dependency

Despite our small sample size, survival rates were similar to published estimates for 0‐ to 1st‐year southern elephant seals (McMahon et al., 1999, 2015; Pistorius & Bester, 2002). In addition, males were twice as likely to die as females, which is congruent with previous studies on southern elephant seals (McMahon, Burton, & Bester, 2003) alongside other pinnipeds (e.g., gray seals; Hall et al., 2001). This sex difference may stem from early‐year developmental disparities in dive and forage ability (Carter et al., 2017).

### 4.2 Initial condition at departure

In contrast to previous findings (e.g., Baker, 2008; Hall et al., 2001; McMahon et al., 2003; McMahon et al., 2015), we did not find departure condition (i.e., drift rate, weight, or date (better condition individuals may leave later); Arnbom et al., 1993, Hindell et al., 1999) to be indicative of survival outcome. This may be reflective of our small sample size and/or range of weights/initial departure conditions (Table 2 and Supporting Information S.11). For example, the maximum estimated departure weight in our sample was 96.1 kg, but studies elsewhere on juvenile elephant seals often include individuals exceeding 135 kg (McMahon, Burton, & Bester, 2000; McMahon et al., 2003). Nonetheless, of all non‐surviving individuals, the pup that exhibited the most prominent difference in dive and forage ability to that of the surviving group was also the lightest (individual 140074; Figure 4; weight at departure ~44.6 kg vs. mean of all pups ~79.9 ± 17.7 kg).

### 4.3 Changes in condition at sea, temporal patterns in mortality, and possible predation

Across most individuals (surviving and non‐surviving), there was a marked and rapid decrease in drift rates following departure from Kerguelen Islands, which likely reflected the depletion of fat reserves obtained via maternal provisioning prior to weaning (Figure 8; Biuw et al., 2003; Orgeret et al., 2019) and was supportive of our choice of mortality hazard in the horseshoe analysis. Following this initial decrease, all pups experienced a prolonged period of negative buoyancy. While we did not detect direct links between drift rates (either initial or change through time) and survival outcome, all but two pups that died did so during this time. For the 11 that survived, all but three individuals returned to positive buoyancy before returning to Kerguelen Islands. As such, during early life, individuals appear more vulnerable to the adverse consequences of fatigue and starvation when negatively buoyant (and likely of poor body condition). For southern elephant seals, during the initial, outward phase of the first trip at sea, the rapid development of dive and forage skills is thus crucial as fat reserves gained from maternal provisioning diminish (McConnell, Fedak, Burton, Engelhard, & Reijnders, 2002), and a return to positive buoyancy following this appears an important determinant in the decision to end the first trip at sea and return to land (Orgeret et al., 2019). This may also reflect the sufficient development of muscular lean tissue, after which excess energy is stored as fat. The one pup (140071) that died while positively buoyant during return to Kerguelen Islands was performing well right up until the days immediately prior to death (Figures 7 and 8). Here, an abrupt behavioral change occurred, possibly supportive of a predation event (which juvenile pinnipeds may be particularly vulnerable to; Horning & Mellish, 2012). A series of fast movements and extended dives, characteristic of escape‐type behaviors and avoidance, were followed by very slow movements and a dramatic decrease in dive and forage ability, indicative of fatigue/injury. Potential predators of juvenile southern elephant seals in the region include killer whales *Orcinus orca* and sleeper sharks *Somniosus antarcticus* (Van den Pistorius, Meyer, Reisinger, & Kirkman, 2012; Hoff & Morrice, 2007).

### 4.4 Dive and forage ability

Individuals that experienced early mortality appeared to exhibit slower development in dive capability compared to surviving pups. A slowing in the rate at which dive durations increased through time was linked to reduced survival, as was an increase in swimming effort through time (i.e., individuals are using more energy to do less). This pattern appeared most prominent for pups 140067, 140074, 140076, and 140078. Temporal improvements in dive capability (e.g., duration) are a key component of diving marine predator ontogeny, as juveniles begin life with abilities that are significantly reduced compared to adults (Burns, 1999; Carter et al., 2017; Orgeret et al., 2019, 2016). Our observations support this and suggest that failure to develop in a timely manner increases mortality risk.

**Figure 10:**
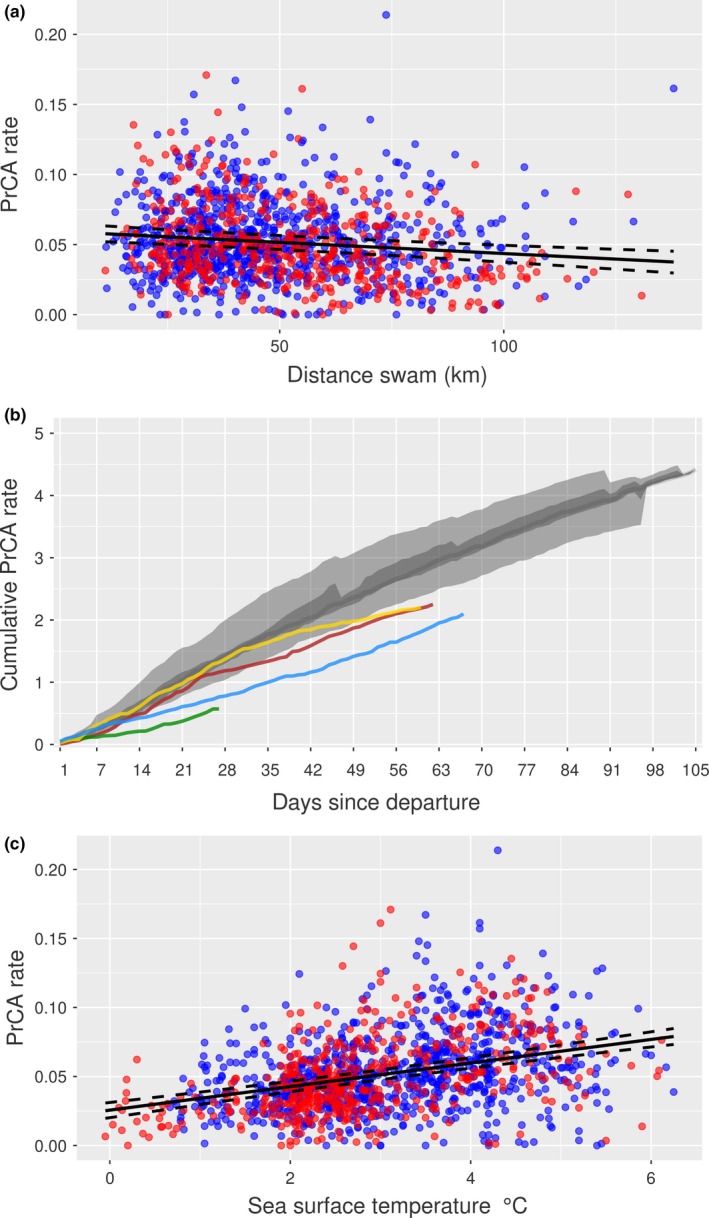
From top to bottom: (a) daily mean PrCA rates against total distances swam, (b) cumulative daily mean prey catch attempts (PrCA), and (c) daily mean PrCA rates against daily mean sea surface temperatures. In subplots (a) and (c), non‐survivors are red and survivors blue. The line of best fit from linear mixed‐effects models (with random intercept of individual ID; nlme package in R—Pinheiro & Bates, 2014) is in black, and dashed lines are 95% confidence intervals. In subplot (b), colored lines are pups 140076 (yellow), 140061 (red), 140078 (blue), and 140074 (green). The large light gray band represents the 2.5%–97.5% quantiles of the survival dataset, the nested dark gray band the 25%–75% quantiles, and the dark gray line the daily median

In addition, non‐surviving individuals moved faster (horizontally) than survivors, which may betray reduced foraging ability. Upon location of profitable foraging grounds, animals tend to reduce travel speeds and increase turning angles, so as to focus efforts in areas where prey are more likely to be encountered (Fauchald & Tveraa, 2003). PrCA rates increased when distances traveled decreased (Figure 10), and so increased mortality with increased travel could relate to failure to locate and/or exploit sufficient resources. For four non‐survivors, PrCA rates were reduced either continually, periodically, at the beginning of a trip, or just prior to death. This was particularly pronounced when plotted cumulatively (i.e., taking the additive mean PrCA rate per individual per day; Figure 10), which suggests that even if an individual manages to increase its PrCA rate after a period when it is low, it cannot “catch up.” We also found individuals that reduced their average PrCA rates through time had a better survival outcome than those that did not, or did so to a lesser extent. This was not generally accompanied by further decreases in buoyancy/drift rate, and in some cases coincided with a positive change in buoyancy/drift rate (Figure 8—from ~day 50 onward). Such a trend supports a change in diet composition and/or improvement of forage ability. For example, in contrast to older individuals that feed predominantly on fish and squid (Slip, 1995), juvenile southern elephant seals have a high proportion of crustaceans (e.g., krill) in their diet (Lubcker et al., 2017; Walters et al., 2014), while the size of other preys (e.g., squid) is generally smaller (Field, Bradshaw, Hoff, Burton, & Hindell, 2007; Slip, 1995). As foraging skills improve, diet composition may change as individuals are able to expand/select/switch to other, possibly more nutrient‐rich prey sizes and/or types, thus requiring less prey items to be caught, resulting in a decrease in PrCA rate (Chaigne, Authier, Richard, Cherel, & Guinet, 2013; Field et al., 2007; Walters et al., 2014). Additionally/alternatively, initial high PrCA rates may include a larger number of unsuccessful attempts, which would decrease in frequency as individuals hone their capture techniques.

### 4.5 Encountered environmental conditions

Non‐surviving juvenile southern elephant seals from Kerguelen Islands encountered waters of lower temperature and reduced TKE compared to the surviving group. Here, PrCA rates were typically reduced (Figure 10 and Supporting Information S.12). While confounding processes (e.g., temporal changes in foraging success and/or prey quality) suggest increased PrCA rates are not necessarily directly linked to better foraging (Orgeret et al., 2019; Richard et al., 2016), combined with increased survival rates these patterns suggest foraging habitats associated with increased SSTs and TKE estimates are more suitable for southern elephant seals during this time in their development.

Across the spatial domain visited by our tracked southern elephant seal pups, areas of higher SST and increased TKE correspond roughly to the southern edge of the polar front, where dynamic habitats associated with frontal zones and eddy edges are more likely to be found (Bost et al., 2009; Supporting Information S.13). Both juvenile and adult southern elephant seals are known to forage within such regions (Cotté, d'Ovidio, Dragon, Guinet, & Lévy, 2015; Dragon, Monestiez, Bar‐Hen, & Guinet, 2010; Field, Bradshaw, Burton, & Hindell, 2004; Tosh et al., 2012), where increased prey availability and abundance can be found (Abrahms et al., 2018; Bost et al., 2009; Cotté et al., 2015). These habitats may thus be particularly important during early life, when juveniles need to compensate reduced dive and forage ability (Burns, 1999). Our results support this and suggest that failure to locate such regions increases mortality risk. All but two pups (one survivor and one non‐survivor) departed Kerguelen in a southeastward direction, and broad‐scale spatial distributions between the surviving and non‐surviving groups were similar with no evidence of distinct segregation, further highlighting the importance of fine‐scale habitat use within a larger common area. Whether the successful location of such features is down to chance or driven by individual innate knowledge and/or other cues is unknown.

## 5 CONCLUSIONS

The first few months following independence represent a particularly critical time in the life cycle of long‐lived marine predators such as the southern elephant seal. Results from this study both support previous assertions that juvenile mortality rates during this period are high and additionally provide direct evidence that this is driven, at least partly, by insufficient development of dive and forage ability. Furthermore, the habitat type encountered, as depicted by SST and TKE, appears to be important to survival outcome as does predation. In all instances, chance seems to play a role, and individuals need only deviate slightly from the norm to increase their mortality risk. As such, survival rates in juvenile southern elephant seals (and possibly other long‐lived marine predators) may be particularly vulnerable to even small changes in climate and environment, which will have concomitant consequences on demography and population dynamics.

## CONFLICT OF INTEREST

None declared.

## AUTHOR CONTRIBUTIONS

SLC, MA, FO, HW, and CG conceived the ideas and designed methodology; FO, HW, and CG collected the data; SLC and MA analyzed the data; SLC led the writing of the manuscript. All authors contributed critically to the drafts and gave final approval for publication.

## Data Availability

Matlab and R codes and scripts used to process and analyze the data are available under GitHub account “SamLCox” or within the Supporting Information. Data used in analysis are available at Dryad: https://doi.org/10.5061/dryad.n5tb2rbrq.

## References

[ece35905-bib-0001] Abrahms, B. , Scales, K. L. , Hazen, E. L. , Bograd, S. J. , Schick, R. S. , Robinson, P. W. , & Costa, D. P. (2018). Mesoscale activity facilitates energy gain in a top predator. Proceedings of the Royal Society B: Biological Sciences, 285, 20181101 10.1098/rspb.2018.1101 PMC6125900 30135161

[ece35905-bib-0002] Arnbom, T. , Fedak, M. A. , Boyd, I. L. , & McConnell, B. J. (1993). Variation in weaning mass of pups in relation to maternal mass, postweaning fast duration, and weaned pup behaviour in southern elephant seals (*Mirounga leonina*) at South Georgia. Canadian Journal of Zoology, 71, 1772–1781.

[ece35905-bib-0003] Baker, J. D. (2008). Variation in the relationship between offspring size and survival provides insight into causes of mortality in Hawaiian monk seals. Endangered Species Research, 5, 55–64. 10.3354/esr00122

[ece35905-bib-0004] Beauplet, G. , Barbraud, C. , Chambellant, M. , & Guinet, C. (2005). Interannual variation in the post‐weaning and juvenile survival of subantarctic fur seals: Influence of pup sex, growth rate and oceanographic conditions. Journal of Animal Ecology, 74, 1160–1172. 10.1111/j.1365-2656.2005.01016.x

[ece35905-bib-0005] Biuw, M. , McConnell, B. , Bradshaw, C. J. , & Fedak, M. (2003). Blubber and buoyancy: Monitoring the body condition of free‐ranging seals using simple dive characteristics. Journal of Experimental Biology, 206, 3405–3423. 10.1242/jeb.00583 12939372

[ece35905-bib-0006] Bost, C. A. , Cotté, C. , Bailleul, F. , Cherel, Y. , Charrassin, J. B. , Guinet, C. , … Weimerskirch, H. (2009). The importance of oceanographic fronts to marine birds and mammals of the southern oceans. Journal of Marine Systems, 78, 363–376. 10.1016/j.jmarsys.2008.11.022

[ece35905-bib-0007] Breed, G. A. , Bowen, W. D. , & Leonard, M. L. (2013). Behavioural signature of intraspecific competition and density dependence in colony‐breeding marine predators. Ecology and Evolution, 3, 3838–3854. 24198943 10.1002/ece3.754 PMC3810878

[ece35905-bib-0008] Burns, J. (1999). The development of diving behavior in juvenile Weddell seals: Pushing physiological limits in order to survive. Canadian Journal of Zoology, 77, 737–747. 10.1139/z99-022

[ece35905-bib-0009] Carpenter, B. , Gelman, A. , Hoffman, M. D. , Lee, D. , Goodrich, B. , Betancourt, M. , … Riddell, A. (2017). Stan: A probabilistic programming language. Journal of Statistical Software, 76, 1–32. 10.18637/jss.v076.i01 PMC9788645 36568334

[ece35905-bib-0010] Carter, M. I. D. , Russell, D. J. F. , Embling, C. B. , Blight, C. J. , Thompson, D. , Hosegood, P. , & Bennett, K. A. (2017). Intrinsic and extrinsic factors drive ontogeny of early‐life at‐sea behaviour in a marine top predator. Scientific Reports, 7, 15505 10.1038/s41598-017-15859-8 29138511 PMC5686064

[ece35905-bib-0011] Chaigne, A. , Authier, M. , Richard, P. , Cherel, Y. , & Guinet, C. (2013). Shift in foraging grounds and diet broadening during ontogeny in southern elephant seals from Kerguelen Islands. Marine Biology, 160, 977–986. 10.1007/s00227-012-2149-5

[ece35905-bib-0012] Costa, D. P. (1991). Reproductive and foraging energetics of high latitude penguins, albatrosses and pinnipeds: Implications for life history patterns. American Zoologist, 31, 111–130. 10.1093/icb/31.1.111

[ece35905-bib-0013] Cotté, C. , d'Ovidio, F. , Dragon, A. C. , Guinet, C. , & Lévy, M. (2015). Flexible preference of southern elephant seals for distinct mesoscale features within the Antarctic Circumpolar Current. Progress in Oceanography, 131, 46–58. 10.1016/j.pocean.2014.11.011

[ece35905-bib-0014] Cox, D. R. (1972). Regression models and life‐tables. Journal of the Royal Statistical Society: Series B (Methodological), 34, 187–220. 10.1111/j.2517-6161.1972.tb00899.x

[ece35905-bib-0015] Cox, S. L. , Orgeret, F. , Gesta, M. , Rodde, C. , Heizer, I. , Weimerskirch, H. , & Guinet, C. (2018). Processing of acceleration and dive data on‐board satellite relay tags to investigate diving and foraging performance in free‐ranging marine predators. Methods in Ecology and Evolution, 9, 64–77. 29456829 10.1111/2041-210X.12845 PMC5812097

[ece35905-bib-0016] Daunt, F. , Afanasyev, V. , Adam, A. , Croxall, J. P. , & Wanless, S. (2007). From cradle to early grave: Juvenile mortality in European shags *Phalacrocorax aristotelis* results from inadequate development of foraging proficiency. Biology Letters, 3, 371–374. 17504733 10.1098/rsbl.2007.0157 PMC2390668

[ece35905-bib-0017] de Grissac, S. , Bartumeus, F. , Cox, S. L. , Weimerskirch, H. (2017). Early‐life foraging: Behavioural responses of newly fledged albatrosses to environmental conditions. Ecology and Evolution, 7, 6766–6778. 28904758 10.1002/ece3.3210 PMC5587467

[ece35905-bib-0018] Dee, D. P. , Uppala, S. M. , Simmons, A. J. , Berrisford, P. , Poli, P. , Kobayashi, S. , … Vitart, F. (2011). The ERA‐Interim reanalysis: Configuration and performance of the data assimilation system. Quarterly Journal Royal Meteorological Society, 137, 553–597. 10.1002/qj.828

[ece35905-bib-0019] Dragon, A. , Monestiez, P. , Bar‐Hen, A. , & Guinet, C. (2010). Linking foraging behaviour to physical oceanographic structures: Southern elephant seals and mesoscale eddies east of Kerguelen Islands. Progress in Oceanography, 87, 61–71. 10.1016/j.pocean.2010.09.025

[ece35905-bib-0020] Drymon, J. M. , & Wells, R. J. D. (2017). Double tagging clarifies post‐release fate of great hammerheads (*Sphyrna mokarran*). Anim Biotelemetry, 5, 28 10.1186/s40317-017-0143-x

[ece35905-bib-0021] Fauchald, P. , & Tveraa, T. (2003). Using first‐passage time in the analysis of area‐restricted search and habitat selection. Ecology, 84, 282–288. 10.1890/0012-9658(2003)084[0282:UFPTIT]2.0.CO;2

[ece35905-bib-0022] Fieberg, J. , & Kochanny, C. O. (2005). Quantifying home‐range overlap: the importance of the utilization distribution. The Journal of Wildlife Management, 69, 1346–1359.

[ece35905-bib-0023] Field, I. C. , Bradshaw, C. J. A. , Burton, H. R. , & Hindell, M. A. (2004). Seasonal use of oceanographic and fisheries management zones by juvenile southern elephant seals (*Mirounga leonina*) from Macquarie Island. Polar Biology, 27, 432–440. 10.1007/s00300-004-0615-3

[ece35905-bib-0024] Field, I. C. , Bradshaw, C. J. A. , Van den Hoff, J. , Burton, H. R. , & Hindell, M. A. (2007). Age‐related shifts in the diet composition of southern elephant seals expand overall foraging niche. Marine Biology, 150, 1441–1452. 10.1007/s00227-006-0417-y

[ece35905-bib-0025] Friedman, J. , Hastie, T. , Simon, N. , Qian, J. , & Tibshirani, R. (2017). Glmnet: Lasso and elastic‐net regularized generalized linear models.

[ece35905-bib-0026] Friedman, J. , Hastie, T. , & Tibshirani, R. (2010). Regularization paths for generalized linear models via coordinate descent. Journal of Statistical Software, 33, 1–22. 10.18637/jss.v033.i01 20808728 PMC2929880

[ece35905-bib-0027] Gaspar, P. , Georges, J.‐Y. , Fossette, S. , Lenoble, A. , Ferraroli, S. , & Le Maho, Y. (2006). Marine animal behaviour: Neglecting ocean currents can lead us up the wrong track. Proceedings of the Royal Society B: Biological Sciences, 273, 2697–2702. 10.1098/rspb.2006.3623 PMC1635505 17015330

[ece35905-bib-0028] Gordine, S. A. , Fedak, M. , & Boehme, L. (2015). Fishing for drifts: Detecting buoyancy changes of a top marine predator using a step‐wise filtering method. Journal of Experimental Biology, 218, 3816–3824. 10.1242/jeb.118109 26486362 PMC4712810

[ece35905-bib-0029] Grambsch, P. M. , & Therneau, T. M. (1994). Proportional hazards tests and diagnostics based on weighted residuals. Biometrika, 81, 515–526. 10.1093/biomet/81.3.515

[ece35905-bib-0030] Guinet, C. (1994). Poids a la naissance et croissance des éléphants de mer austraux. Quelles informations nous apportent‐ils sur le milieu marin? Recueil De Medecine Veterinaire, 170, 105–110.

[ece35905-bib-0031] Guinet, C. , Jouventin, P. , & Weimerskirch, H. (1992). Population changes, movements of southern elephant seals on Crozet and Kerguelen Archipelagos in the last decades. Polar Biology, 12, 349–356. 10.1007/BF00243106

[ece35905-bib-0032] Hall, A. J. , McConnell, B. , & Barker, R. J. (2001). Factors affecting first‐year survival in grey seals and their implications for life history strategies. Journal of Animal Ecology, 70, 138–149.

[ece35905-bib-0033] Hart, K. M. , & Hyrenbach, K. D. (2009). Satellite telemetry of marine megavertebrates: The coming of age of experimental science. Endangered Species Research, 10, 9–20.

[ece35905-bib-0034] Hays, G. C. , Bradshaw, C. J. A. , James, M. C. , Lovell, P. , & Sims, D. W. (2007). Why do Argos satellite tags deployed on marine animals stop transmitting. Journal of Experimental Marine Biology and Ecology, 349, 52–60. 10.1016/j.jembe.2007.04.016

[ece35905-bib-0035] Hazen, E. L. , Maxwell, S. M. , Bailey, H. , Bograd, S. J. , Hamann, M. , Gaspar, P. , … Shillinger, G. L. (2012). Ontogeny in marine tagging and tracking science: Technologies and data gaps. Marine Ecology Progress Series, 457, 221–240. 10.3354/meps09857

[ece35905-bib-0036] Heerah, K. , Cox, S. L. , Blevin, P. , Guinet, C. , & Charrassin, J. B. (2019). Validation of dive foraging indices using archived and transmitted acceleration data: The case of the Weddell seal. Frontiers in Ecology and Evolution, 7, 30 10.3389/fevo.2019.00030

[ece35905-bib-0037] Henderson, R. , Diggle, P. , & Dobson, A. (2000). Joint modelling of longitudinal measurements and event time data. Biostatistics, 1, 465–480. 10.1093/biostatistics/1.4.465 12933568

[ece35905-bib-0038] Hindell, M. A. , McConnell, B. J. , Fedak, M. A. , Slip, D. J. , Burton, H. R. , Reijnders, P. J. H. , & McMahon, C. R. (1999). Environmental and physiological determinants of successful foraging by naive southern elephant seal pups during their first trip to sea. Canadian Journal of Zoology, 77, 1807–1821. 10.1139/z99-154

[ece35905-bib-0039] Hindell, M. A. , McMahon, C. R. , Bester, M. N. , Boehme, L. , Costa, D. , Fedak, M. A. , … Charrassin, J. B. (2016). Circumpolar habitat use in the southern elephant seal: Implications for foraging success and population trajectories. Ecosphere, 7, e01213 10.1002/ecs2.1213

[ece35905-bib-0040] Horning, M. , & Mellish, J.‐A.‐E. (2012). Predation on an upper trophic marine predator, the Steller sea lion: Evaluating high juvenile mortality in a density dependent conceptual framework. PLoS One, 7, e30173 10.1371/journal.pone.0030173 22272296 PMC3260237

[ece35905-bib-0041] Irvine, L. G. , Hindell, M. A. , van den Hoff, J. , & Burton, H. R. (2000). The influence of body size on dive duration of underyearling southern elephant seals (*Mirounga leonina*). Journal of Zoology, 251, 463–471.

[ece35905-bib-0042] Jonsen, I. D. , Flemming, J. M. , & Myers, R. A. (2005). Robust state‐space modelling of animal movement data. Ecology, 86, 2874–2880.

[ece35905-bib-0043] Lea, M. A. , Johnson, D. , Ream, R. , Sterling, J. , Melin, S. , & Gelatt, T. (2009). Extreme weather events influence dispersal of naive northern fur seals. Biology Letters, 5, 252–257. 10.1098/rsbl.2008.0643 19147444 PMC2665815

[ece35905-bib-0044] Lubcker, N. , Reisinger, R. R. , Oosthuizen, W. C. , Nico de Bruyn, P. J. , van Tonder, A. , Pistorius, P. A. , & Bester, M. N. (2017). Low trophic level diet of juvenile southern elephant seals *Mirounga leonina* from Marion Island: A stable isotope investigation using vibrissal regrowths. Marine Ecology Progress Series, 577, 237–250. 10.3354/meps12240

[ece35905-bib-0045] McConnell, B. , Fedak, M. , Burton, H. R. , Engelhard, G. H. , & Reijnders, P. J. H. (2002). Movements and foraging areas of naive, recently weaned southern elephant seal pups. Journal of Animal Ecology, 71, 65–78. 10.1046/j.0021-8790.2001.00576.x

[ece35905-bib-0046] McMahon, C. R. , & Burton, H. R. (2005). Climate change and seal survival: Evidence for environmentally mediated changes in elephant seal, *Mirounga leonina*, pup survival. Proceedings of the Royal Society B: Biological Sciences, 272, 923–928. 10.1098/rspb.2004.3038 PMC1564088 16024347

[ece35905-bib-0047] McMahon, C. R. , Burton, H. R. , & Bester, M. N. (1999). First‐year survival of southern elephant seals, *Mirounga leonina*, at sub‐Antarctic Macquarie Island. Polar Biology, 21, 279–284. 10.1007/s003000050363

[ece35905-bib-0048] McMahon, C. R. , Burton, H. R. , & Bester, M. N. (2000). Weaning mass and the future survival of juvenile southern elephant seals, *Mirounga leonina*, at Macquarie Island. Antarctic Science, 12, 149–153.

[ece35905-bib-0049] McMahon, C. R. , Burton, H. R. , & Bester, M. N. (2003). A demographic comparison of two southern elephant seal population. Journal of Animal Ecology, 72, 61–74.

[ece35905-bib-0050] McMahon, C. R. , Field, I. C. , Bradshaw, C. J. A. , White, G. C. , & Hindell, M. A. (2008). Tracking and data‐logging devices attached to elephant seals do not affect individual mass gain or survival. Journal of Experimental Biology, 360, 71–77. 10.1016/j.jembe.2008.03.012

[ece35905-bib-0051] McMahon, C. R. , Hindell, M. A. , Burton, H. R. , & Bester, M. N. (2005). Comparison of southern elephant seal populations, and observations of a population on a demographic knife‐edge. Marine Ecology Progress Series, 288, 273–283. 10.3354/meps288273

[ece35905-bib-0052] McMahon, C. R. , New, L. F. , Fairley, E. J. , Hindell, M. A. , & Burton, H. R. (2015). The effects of body size and climate on post‐weaning survival of elephant seals at Heard Island. Journal of Zoology, 297, 301–308. 10.1111/jzo.12279

[ece35905-bib-0053] Mitani, Y. , Andrews, R. D. , Sato, K. , Kato, A. , Naito, Y. , & Costa, D. P. (2010). Three‐dimensional resting behaviour of northern elephant seals: Drifting like a falling leaf. Biology Letters, 6, 163–166. 10.1098/rsbl.2009.0719 19864274 PMC2865059

[ece35905-bib-0054] Orgeret, F. , Cox, S. L. , Weimerskirch, H. , & Guinet, C. (2019). Body condition influences ontogeny of foraging behaviour in juvenile southern elephant seas. Ecology and Evolution, 9, 223–236. 30680109 10.1002/ece3.4717 PMC6341977

[ece35905-bib-0055] Orgeret, F. , Weimerskirch, H. , & Bost, C. A. (2016). Early diving behaviour in juvenile penguins: Improvement or selection processes. Biology Letters, 12, 20160490 10.1098/rsbl.2016.0490 27484650 PMC5014042

[ece35905-bib-0056] Oro, D. , Torres, R. , Rodriguez, C. , & Drummond, H. (2010). Climatic influence on demographic parameters of a tropical seabird varies with age and sex. Ecology, 91, 1205–1214. 10.1890/09-0939.1 20462134

[ece35905-bib-0057] Pavlou, M. , Ambler, G. , Seaman, S. , De Iorio, M. , & Omar, R. Z. (2016). Review and evaluation of penalised regression methods for risk prediction in low‐dimensional data with few events. Statistics in Medicine, 35, 1159–1177. 26514699 10.1002/sim.6782 PMC4982098

[ece35905-bib-0058] Photopoulou, T. , Lovell, P. , Fedak, M. A. , Thomas, L. , & Matthiopoulos, J. (2015). Efficient abstracting of dive profiles using a broken‐stick model. Methods in Ecology and Evolution, 6, 278–288. 10.1111/2041-210X.12328

[ece35905-bib-0059] Piironen, J. , & Vehtari, A. (2017). Sparsity information and regularization in the horseshoe and other shrinkage priors. Electronic Journal of Statistics, 11, 5018–5051. 10.1214/17-EJS1337SI

[ece35905-bib-0060] Pinheiro, J. , & Bates, D. M. (2014). nlme: Linear and nonlinear mixed effects models.

[ece35905-bib-0061] Pistorius, P. , & Bester, M. N. (2002). Juvenile survival and population regulation in southern elephant seals at Marion Island. African Zoology, 37, 35–41. 10.1080/15627020.2002.11657152

[ece35905-bib-0062] Pistorius, P. A. , Meyer, M. A. , Reisinger, R. R. , & Kirkman, S. P. (2012). Killer whale predation on subantarctic fur seals at Prince Edward Island, Southern Indian Ocean. Polar Biology, 35, 1767–1772. 10.1007/s00300-012-1216-1

[ece35905-bib-0063] Reiter, J. , Stinson, N. L. , & Le Boeuf, B. J. (1978). Northern elephant seal development: The transition from weaning to nutritional independence. Behavioral Ecology and Sociobiology, 3, 337–367. 10.1007/BF00303199

[ece35905-bib-0064] Richard, G. , Cox, S. L. , Picard, B. , Vacquie‐Garcia, J. , & Guinet, C. (2016). Southern elephant seals replenish their lipid reserves at different rates according to foraging habitat. PLoS One, 11, e0166747 10.1371/journal.pone.0166747 27902786 PMC5130208

[ece35905-bib-0065] Richard, G. , Vacquie‐Garcia, J. , Jouma'a, J. , Picard, B. , Genin, A. , Arnould, J. P. Y. , … Guinet, C. (2014). Variation in body condition during the post‐moult foraging trip of southern elephant seals and its consequences on diving behaviour. Journal of Experimental Biology, 217, 2609–2619. 10.1242/jeb.088542 24803471

[ece35905-bib-0066] Riotte‐Lambert, L. , & Weimerskirch, H. (2013). Do naive juvenile seabirds forage differently from adults? Proceedings of the Royal Society B: Biological Sciences, 280, 20131434 10.1098/rspb.2013.1434 PMC3757974 23926153

[ece35905-bib-0067] Simon, N. , Friedman, J. , Hastie, T. , & Tibshirani, R. (2011). Regularization paths for Cox's proportional hazards model via coordinate descent. Journal of Statistical Software, 39, 1–13. 10.18637/jss.v039.i05 PMC4824408 27065756

[ece35905-bib-0068] Slip, D. J. (1995). The diet of southern elephant seals (*Mirounga leonina*) from Heard Island. Canadian Journal of Zoology, 73, 1519–1528.

[ece35905-bib-0069] Therneau, T. M. , & Lumley, T. (2017). Survival.

[ece35905-bib-0070] Tibshirani, R. (1997). The LASSO method for variable selection in the Cox model. Statistics in Medicine, 16, 385–395. 10.1002/(SICI)1097-0258(19970228)16:4<385:AID-SIM380>3.0.CO;2-3 9044528

[ece35905-bib-0071] Tosh, C. A. , Steyn, J. , Bornemann, H. , Ven den Hoff, J. , Stewart, B. S. , Plotz, J. , & Bester, M. N. (2012). Marine habitats of juvenile southern elephant seals from Marion Island. Aquatic Biology, 17, 71–79. 10.3354/ab00463

[ece35905-bib-0072] Van den Hoff, J. , & Morrice, M. G. (2007). Sleeper sharks (*Somniosus antarcticus*) and other bite wounds observed on southern elephant seals (*Mirounga leonina*) at Macquarie Island. Marine Mammal Science, 42, 239–247.

[ece35905-bib-0073] Viviant, M. , Trites, A. W. , Rosen, D. A. S. , Monestiez, P. , & Guinet, C. (2010). Prey capture attempts can be detected in Steller sea lions and other marine predators using accelerometers. Polar Biology, 33, 713–719. 10.1007/s00300-009-0750-y

[ece35905-bib-0074] Volpov, B. L. , Hoskins, A. J. , Battaile, B. C. , Viviant, M. , Wheatley, K. E. , Marshall, G. , … Arnould, J. P. Y. (2015). Identification of prey captures in Australian fur seals (*Arctocephalus pusillus doriferus*) using head‐mounted accelerometers: Field validation with animal‐born video cameras. PLoS One, 10, e0128789. 26107647 10.1371/journal.pone.0128789 PMC4479472

[ece35905-bib-0075] Walters, A. , Lea, M. A. , Van den Hoff, J. , Field, I. C. , Virtue, P. , Sokolov, S. , … Hindell, M. A. (2014). Spatially explicit estimates of prey consumption reveal a new krill predator in the southern ocean. PLoS One, 9, e86452 10.1371/journal.pone.0086452 24516515 PMC3905967

[ece35905-bib-0076] Yang, J. , Zhu, H. , Choi, T. , & Cox, D. D. (2016). Smoothing and mean‐covariance estimation of functional data with a Bayesian hierarchical model. Bayesian Analysis, 11, 649–670. 10.1214/15-BA967 PMC8387981 34457106

[ece35905-bib-0077] Zou, H. , & Hastie, T. (2005). Regularization and variable selection via the elastic net. Journal of the Royal Statistical Society: Series B (Statistical Methodology), 67, 301–320. 10.1111/j.1467-9868.2005.00503.x

