# Supplementary material for: High mortality rates in a juvenile free‐ranging marine predator and links to dive and forage ability
Source: Ecol Evol. 2019 Dec 11;10(1):410–30. doi: 10.1002/ece3.5905 (PMC6972805; doi:10.1002/ece3.5905)
Supplement: Supplementary file 1 [file ECE3-10-410-s001.pdf]

<u>SUPPLEMENTARY INFORMATION</u>

S.1 Individual deployment metrics

S.2 Processing of Argos location data

S.3 Drift segment/dive identification and estimation of daily drift rates

S.4 Investigations into potential sampling biases

S.5 Broad-scale spatial distributions of surviving and non-surviving individuals

S.6 Survival analysis with a regularized horseshoe prior

S.7 Retrospective power analysis of the survival analysis using a regularized horseshoe prior

S.8 Visual comparisons of behaviours and encountered environmental conditions of non-surviving pups to those of grouped survivors

S.9 Comparisons between survivors and non-survivors of maximum total (eddy) kinetic energy (TKE) values of waters visited

S.10 Variable selection within the horseshoe survival analysis

S.11 Departure weights and conditions

S.12 Prey catch attempt (PrCA) rates and maximum total (eddy) kinetic energy (TKE)

S.13 Spatial distributions of juvenile southern elephant seals in relation to sea surface temperature (SST) and surface currents for four days during the austral summer

S.14 References

S.1 Individual deployment metrics

Table S1. Individual southern elephant seal pup IDs alongside sex, tag deployment date, weight at departure (kg), departure date, estimated departure weight (kg), the last date the DSA tag transmitted, the last date the SPOT tag transmitted, the absolute difference between the last transmission dates of the DSA and SPOT tags (in hours), survival outcome (yes = lived, no = died), the length of the first trip in days (till death or return to Kerguelen Islands), and the total number of dives transmitted during this first trip. All dates are show in a dd/mm/yy format.

Pup ID	Sex (F/M)	Deployment date	Deployment weight (kg)	Departure date	Departure weight (kg)	DSA end date	SPOT end date	Abs. diff. end dates (hrs)	Survival	Length of first trip	Total no. dives
140059	F	03/12/14	88.0	21/12/14	76.1	20/08/15	26/11/15	2358.0	Yes	176.2	1086
140060	M	03/12/14	85.0	12/12/14	79.4	14/06/15	26/11/15	3959.2	Yes	184.1	1192
140061	M	03/12/14	97.0	22/12/14	83.7	23/02/15	23/02/15	18.1	No	63.9	373
140062	F	04/12/14	61.6	08/12/14	59.8	09/09/15	25/11/15	1833.1	Yes	149.6	602
140063	F	04/12/14	78.0	08/12/14	76.0	05/06/15	09/11/15	3788.4	Yes	154.0	798
140064	M	04/12/14	73.4	04/12/14	73.4	13/01/15	12/01/15	3.4	No	39.8	165
140065	M	04/12/14	83.4	07/12/14	82.0	24/12/14	24/12/14	0.4	No	17.6	102
140066	M	26/11/14	64.0	05/12/14	59.2	04/11/15	26/11/15	534.6	Yes	176.1	1023
140067	F	05/12/14	60.6	08/12/14	59.4	04/03/15	04/03/15	4.0	No	85.9	533
140068	F	05/12/14	88.6	16/12/14	81.5	13/10/15	26/11/15	1055.9	Yes	187.7	1206
140069	F	05/12/14	64.8	20/12/14	56.5	19/09/15	11/11/15	1259.8	Yes	202.2	1098
140070	M	05/12/14	98.4	18/12/14	89.3	26/12/14	26/12/14	0.5	No	8.6	61
140071	M	05/12/14	92.4	18/12/14	83.0	09/04/15	09/04/15	1.7	No	111.8	683
140072	M	06/12/14	78.8	14/12/14	74.1	07/09/15	26/11/15	1929.6	Yes	147.9	980
140073	F	05/12/14	74.2	18/12/14	66.5	31/05/15	22/07/15	1244.3	Yes	163.8	952
140074	M	06/12/14	47.2	12/12/14	44.6	09/01/15	09/01/15	5.1	No	28.5	109
140075	F	06/12/14	59.4	11/12/14	57.1	24/09/15	26/11/15	1520.4	Yes	138.2	974
140076	M	06/12/14	116.4	01/01/15	96.1	03/03/15	02/03/15	28.1	No	61.7	356
140077	F	06/12/14	109.4	27/12/14	93.6	22/08/15	13/11/15	2002.6	Yes	184.9	836
140078	F	06/12/14	76.6	21/12/14	67.5	28/02/15	26/02/15	26.1	No	68.6	365

S.2 Processing of Argos location data

Argos location filtering

Argos location data were not equally positioned in time and were or varying quality (specified location class errors; www.argos-system.com). As such, all data were processed through a hierarchical first difference correlated random walk state-space model (SSM), via the R package 'bsam' (Jonsen *et al.*, 2005, 2017; Jonsen, 2016). Two Monte Carlo Markov chains of 150000 iterations, with a burn in of 150000 samples and thinning of 100 (to reduce within chain auto-correlation), were run to obtain filtered location estimates at a temporal resolution of one hour. The quality of the fit of the SSM for each individual was assessed visually (through plots of observed Argos and SSM estimated locations), whilst convergence of the Markov chain Mote Carlo algorithm was confirmed via visual observation of traceplots alongside the Gelman and Rubin's shrink factor plot (Brooks and Gelman, 1998).

Current corrections

Because interactions with oceanic currents can distort the observed trajectories of an individual (Gaspar *et al.*, 2006), prior to the generation of movement metrics, at-sea horizontal swimming behaviours were corrected to remove the influence of ocean currents. Sea surface current velocities were taken from the delayed time all-sat-merged Global Ocean Gridded Absolute Geostrophic Velocities Anomalies L4 product of AVISO (www.aviso.altimetry.fr) on a daily basis at a spatial resolution of 0.25°. Real juvenile swim directions and speeds were then calculated by decomposing each hourly step of a track into its observed velocity components, $u_{observed}$ (east-west) and $v_{observed}$ (north-south). The closest spatially time matched current velocity components, $u_{current}$ and $v_{current}$, were then subtracted from this to give u_{real} and v_{real}. A new heading was calculated as atan2(u_{real}, v_{real}) × 180/π, whilst speed was taken for the hourly segment as $\sqrt{u_{real}{}^2 + v_{real}{}^2}$ (Gaspar *et al.*, 2006; Cotté *et al.*, 2007).

S.3 Drift segment/dive identification and estimation of daily drift rates

Southern elephant seals regularly perform resting dives where individuals cease active movement and "drift" in the water column (Biuw *et al.*, 2003; Mitani *et al.*, 2010; Gordine *et al.*, 2015). Vertical movement during these inactive periods can be used to make inferences on an individual's buoyancy, which in turn reflects body composition (ratio of high-density lean to low-density lipid tissue; Biuw et al. 2003), and so body condition. Animals that are positively buoyant should be in 'better' condition than those that are negatively buoyant (due to increased fat reserves). During their first trip at sea, juvenile elephant seals are still developing and growing, and as such, changes in buoyancy could also reflect the acquisition of lean muscle in addition to changes in fat reserves (Biuw *et al.*, 2003; Orgeret *et al.*, 2019). Drift segment/dive identification was performed using a modified step-wise filtering process similar to that described in Biuw *et al.*, (2003) and Gordine *et al.*, (2015), but which also incorporated information from accelerometer transmissions on prey catch attempt (PrCA) behaviours and swimming effort. First, the first and last broken-stick segments of each dive were removed. Second, all segments within which PrCA behaviours were recorded were removed. Third, histograms of the average swimming efforts of dive segments (scaled by duration) were constructed. From this, a bimodal distribution was evident (Figure S1), and so the break between the two modes at $4ms^{-3}$ was selected as a threshold above which segments were not retained in drift identification (drift dives should have low levels of active movement and thus reduced swimming efforts).

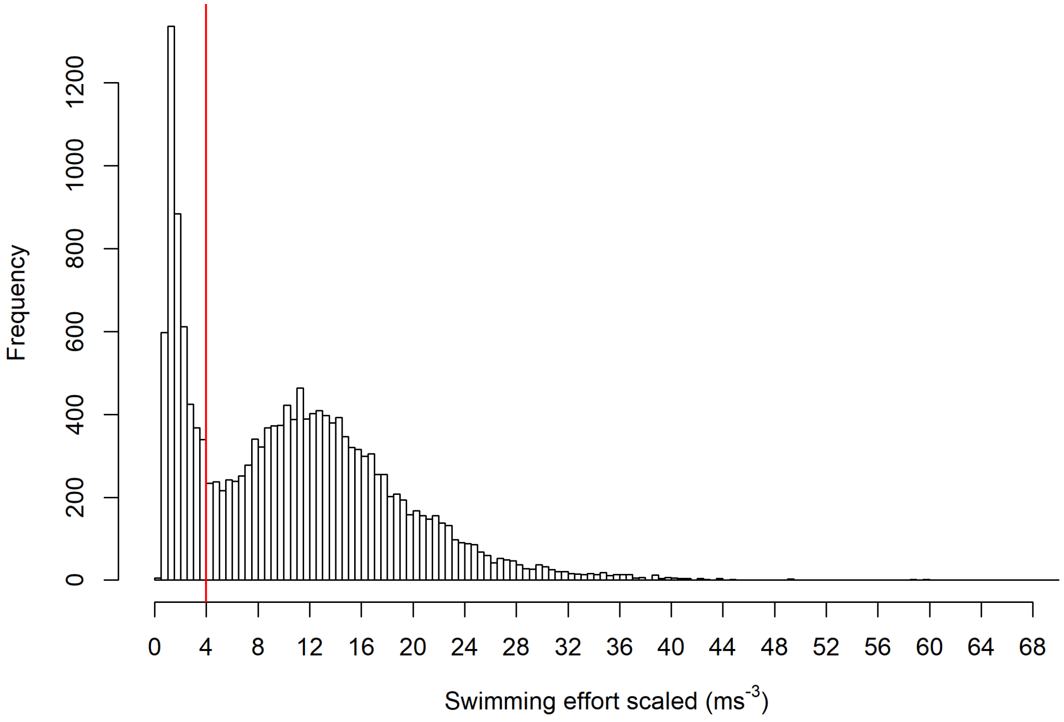

Figure S1. Histogram of average dive segment swimming efforts (scaled by segment duration). A bimodal distribution is noted. A break between the two peaks occurs at around $4ms^{-3}$ (marked by the vertical red line), suggesting the presence of two discrete behavioural groups. Drift segments were associated with the group of lower swimming efforts ($< 4ms^{-3}$).

Fourth, the dive zone index (DZI) of each dive was estimated following Photopoulou *et al.*, (2015). The DZI describes how well a broken-stick model (BSM; Fedak *et al.*, 2001) representation of a dive fits its true profile. High values suggest a profile of more complex trajectory and vertical movement, whilst low values would suggest a dive with a smooth trajectory, such as a drift dive. We constructed a histogram of the DZI's of all dives and cut those segments associated with dives with DZIs above 0.2, which represented the upper limit of the normal distribution of dives thus far retained (Figure S2).

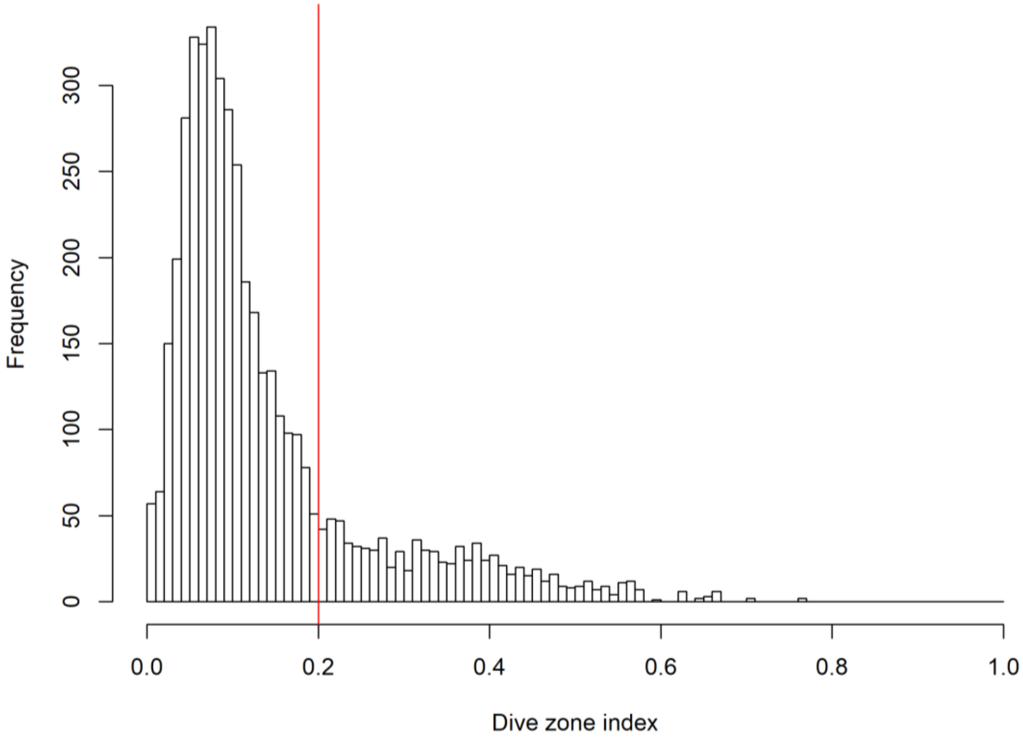

Figure S2. The dive zone indexes (DZI's) of all dives thus far retained (at step four of the step-wise filtering process). Dive segments associated with dives with DZIs greater than 0.2 (vertical red line) were discarded.

Fifth, we constructed histograms of the proportion of a total dive time a segment represented (as drift dives segments should be of extended lengths), and also plotted these values against the absolute vertical speed ($\text{depth}_{(t)} - \text{depth}_{(t+1)}$ where t is time in seconds) of the segment. We found that shorter segment proportions were associated with unrealistic absolute vertical drift rates (sometimes in excess of 1.5ms^{-1}; Biuw *et al.*, 2003; Gordine *et al.*, 2015), and so we discarded all segments with dive proportions of less than 0.15 (which was also the natural break in the histogram of all segment dive proportions; Figure S3). Sixth, we calculated the total proportion of a dive that was comprised of drift segments (totalling the durations of multiple drift segments). This extends upon the last step by considering the entire dive, rather than individual segments, and allows for instances where the drift phase may have been cut in two by the BSM. Again, dives where all candidate drift segments composed less than 40% of the total dive time (proportion less than 0.4) were associated with more variable and unrealistic drift rates, and so were discarded (Figure S4). Seventh, a histogram of estimated vertical speeds from thus far retained dive segments was constructed and used to identify outlier, unrealistic values. Dive segments with vertical speeds greater than -0.5ms^{-1} and less than

0.3ms^{-1} were retained (Figure S5), which reflects values reported previously by Biuw *et al.*, (2003) and Gordine *et al.*, (2015). Dive segments with vertical speeds of exactly 0ms^{-1} were also discarded as these were thought to represent periods when an individual was resting against the oceans floor (Figure S6; Biuw *et al.*, 2003). Indeed, such estimates generally occurred as outliers when individuals were within shelf-sea waters (i.e. the Kerguelen plateau). For a couple of individuals, this behaviour was verified through inspection of retrieved high resolution archival data (see Cox *et al.*, 2018). Finally, for each individual, all candidate segment drift rates were plotted through time. Outliers were manually examined, and generally excluded. For individuals from whom tags were retrieved, this again included an inspection of the high resolution archival data. Typically, outliers occurred because the BSM failed to adequately capture the correct shape of a drift dive (e.g. in some drift dives with positive vertical speeds, individuals float towards the surface and periodically reposition themselves in the water column, which sometimes looks like a drift dive from a negatively buoyant individual as the BSM captures the end of one drift phase and another, and doesn't filter out the repositioning in the middle).

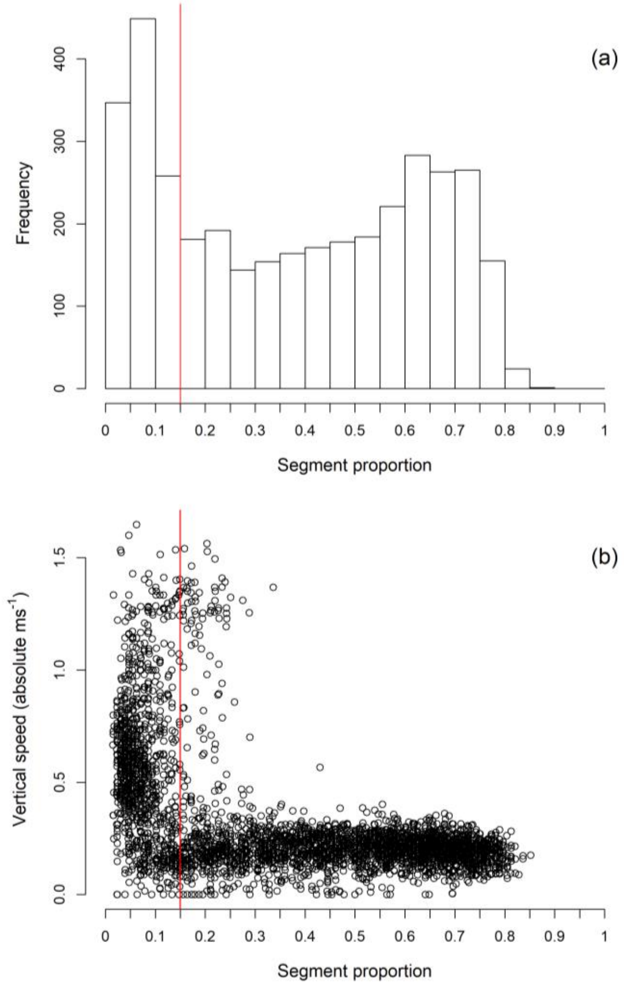

Figure S3. Segment lengths as a proportion of the total dive time in (a) a histogram and (b) a plot against corresponding absolute vertical speeds. Note higher variability in vertical speeds at lower dive segment proportions. Segments with lengths less than 0.15 (vertical red line) of the total dive length were discarded in the step-wise filtering process.

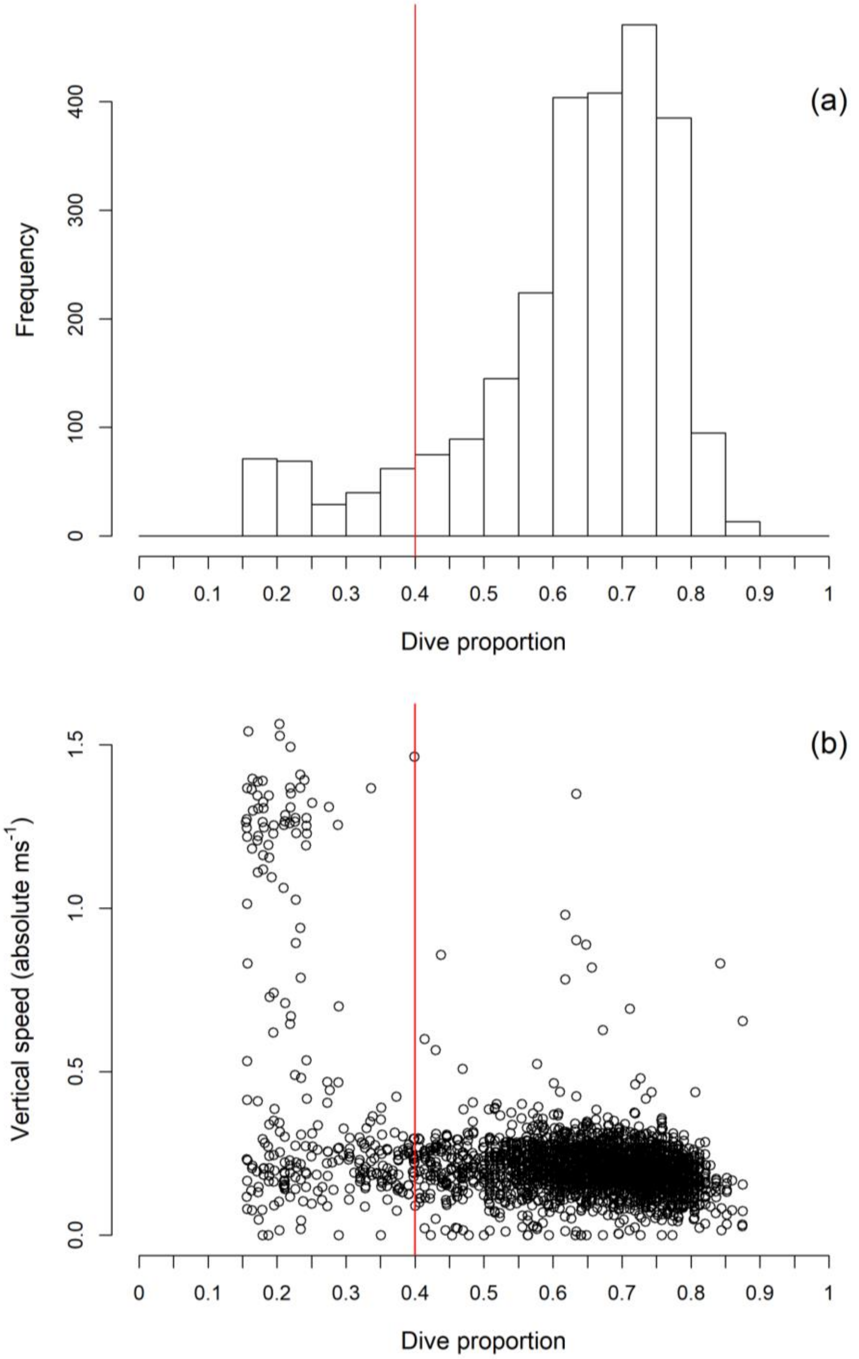

Figure S4. The total length of all candidate drift segments within a dive as a proportion of the total time of a dive as (a) a histogram and (b) a plot against corresponding absolute vertical speeds. Note much higher variability in vertical speeds at lower dive segments proportions. Those segments associated with dives where the total proportion of candidate drift segments was less than 0.4 (vertical red line) of the total dive length were discarded in the step-wise filtering process.

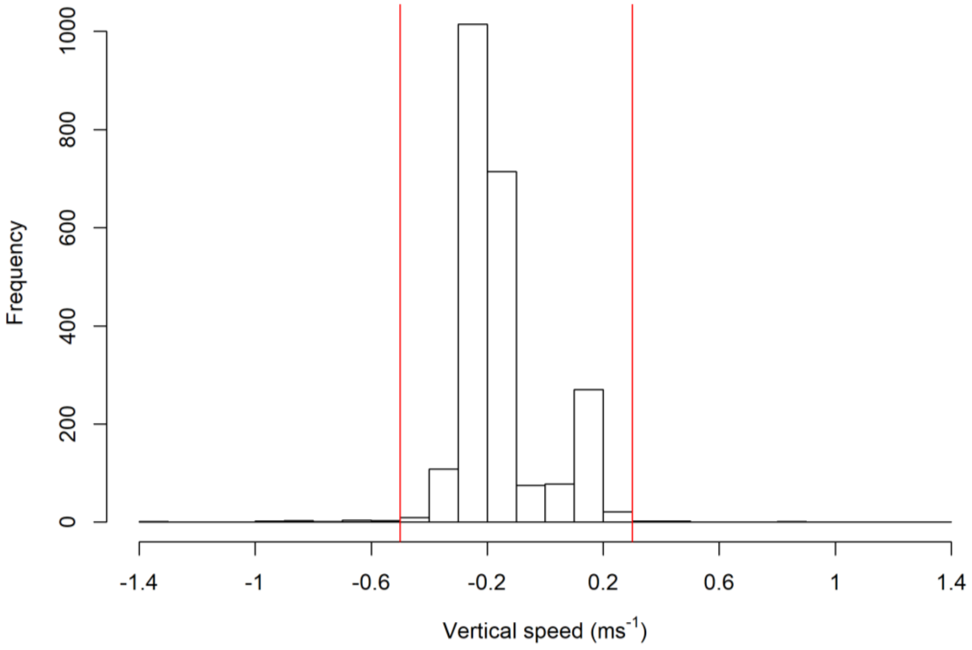

Figure S5. Histogram of segment vertical speeds. Those below -0.5ms^{-1} or above 0.3ms^{-1} (indicated by the vertical red lines) were discarded in the step-wise filtering process.

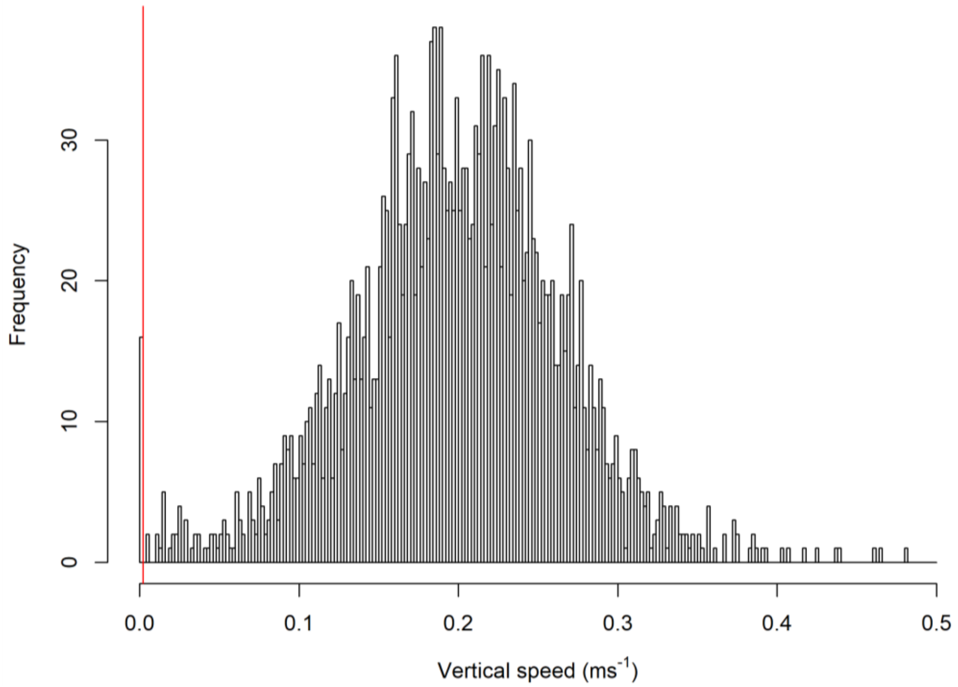

Figure S6. Histogram of absolute segment vertical speeds. Those of exactly 0ms^{-1} were discarded in the step-wise filtering process. These values represent a peak out-with the expected range of absolute vertical speeds following a normal distribution.

Following this step-wise filtering process, we then used functional data analysis to obtain daily drift rate estimates. During the beginning of their first trip at-sea, juvenile southern elephant seals are constrained in their ability to make deep dives (Biuw *et al.*, 2003; Orgeret *et al.*, in prep), and so initial drift rates are often obtained from relatively shallow depths. However, residual air in the lungs can bias these measurements (Kooyman and Ponganis, 1998; Biuw *et al.*, 2003). Rather than excluding such estimates (and loosing information on an individual's condition at the beginning of a trip), all filtered drift segments were retained, and instead we weighted the measurement error of our functional data analysis by the minimum depth recorded during a drift dive segment. Exploratory plots of drift rates with dive depth did not suggest the presence of any large bias in our dataset (Figure S7). Smoothed estimates from the functional data analysis were then used to obtain daily drift rate estimates. These are shown in Figure S8. Full modelling details are as follows:

For each pup equipped with a tag, data on drift rates were available. These data were used to build a hierarchical model and predict daily drift rates during the period when tags were transmitting. The model thus assumes that for each day, there is a 'true' drift rate value, which reflects the (unobservable) body condition of the pup. For some days, one or several measurements of this true value may be available, while for days when no drift dive was performed, there are no such measurements. We assumed that pup body condition was correlated across consecutive days, but that this correlation was in effect negligible after some sufficiently long timespan. Specifically, we assumed a Màtern correlation of order $3/2$ and fixed the range parameter to the value $50/2.738$, meaning that the correlation between two measurements taken 50 days apart is approximately 0.05. Drift rates were envisioned as a continuous curve over the period a tag was emitting, and we used functional data analysis with Gaussian processes for non-parametric modelling of these curves (Yang *et al.*, 2016) to obtain smoothed estimates of daily drift rate. Specifically, each curve was modelled as a realization from a Gaussian Process (Yang *et al.*, 2016). Let y_{ipt} denotes the *i*th measurement of drift rates available for pup *p* on day *t*, and T_p denotes the length of time during which tag was emitting for pup *p*:

$$y_{ipt} \sim N(d_{pt}, \sigma_{ipt}^{\text{error}})$$

where d_{pt} is the 'true' drift rate value at time *t* for pup *p*, and $\sigma_{ipt}^{\text{error}}$ is a scale parameter quantifying measurement error. Measurement error was dependent on the minimum depth recorded during a drift dive segment:

$$\log\left(\sigma_{ipt}^{error}\right) = \alpha_1 + \alpha_2 \times \text{Depth}_{ipt}$$

The vector of 'true' daily drift rates $\boldsymbol{d_p}$ (of length T_p) is modelled as:

$$\boldsymbol{d_p} \sim MVN(\mathbf{1}\mu_p, \sigma_p \boldsymbol{\Omega})$$

where $MVN()$ is the Multivariate Normal distribution μ_p is the mean drift value of pup *p*, and σ_p is a scale parameter quantifying the process variation (volatility) for pup *p*. $\boldsymbol{\Psi}$ is a covariance matrix of dimension $T_p \times T_p$. We used a stationary correlation structure for $\boldsymbol{\Psi}$ with the Màtern parameterization:

$$\Psi(t_1, t_2) = \left(1 + \frac{|t_1 - t_2|\sqrt{3}}{\rho}\right) e^{-|t_1 - t_2|\sqrt{3}/\rho}$$

where $|t_1 - t_2|$ is the distance between days t_1 and t_2, and ρ is a range parameter controlling correlation decay between days t_1 and t_2. The value for ρ was chosen so that:

$$\Psi(t, t + 50) \approx 0.05$$

Finally, the parameters (μ_p, σ_p) were modelled hierarchically to allow borrowing strength across pups:

$$\mu_p \sim N(\mu, \sigma_{\text{pup},1})$$

$$\log(\sigma_p) \sim N(\eta, \sigma_{\text{pup},2})$$

where $\sigma_{\text{pup},1}$ and $\sigma_{\text{pup},2}$ are scale parameters quantifying between pup variation in mean drift rate values and drift rate volatility. This model was implemented in Stan (Carpenter *et al.*, 2017) with weakly informative priors. The Stan code is given below:

```
data {
 int<lower = 1> n_ind; // number of pups
 int<lower = 1> n_day; // number of days during which tags were emitting
 int<lower = 1> n_obs_tot; // total sample size
 real Y[n_obs_tot]; // drift rate data
 int<lower = 1, upper = n_ind> ID[n_obs_tot]; // identifier for pup
 int<lower = 1, upper = n_day> DAY[n_obs_tot]; // identifier for day
 vector[n_obs_tot] DEPTH; // dive depth
 real<lower = 0> prior_rate;
 matrix[n_day, n_day] PSI; // Matern covariance matrix
 vector[2] prior_scale_inter;
 vector[2] prior_scale_alpha;
}
transformed data {
 matrix[n_day, n_day] chol_PSI; // Cholesky decomposition of PSI
 chol_PSI = cholesky_decompose(PSI);
}
parameters {
 real mu;
 real eta;
 vector[2] alpha;
 vector[2] alpha;
 vector[n_ind] mu_pup;
 vector[n_ind] eta_pup;
 vector[n_day] z[n_ind];
 vector<lower=0>[2] tau;
 vector<lower=0>[2] sigma2;
}
transformed parameters {
 vector[2] sigma_ind;
 vector[n_day] drift_rate[n_ind];
 vector[n_obs_tot] sigma_error;
 for(k in 1:2){
  sigma_ind[k] = sqrt(sigma2[k]);
```

```
 }
 for (i in 1:n_ind) {
  drift_rate[i] = rep_vector(mu + mu_pup[i], n_day) + chol_PSI * z[i];
 }
sigma_error = exp(alpha[1] + alpha[2]*DEPTH);
}
model {
 mu ~ normal(0.0, prior_scale_inter);
 alpha ~ normal(0.0, prior_scale_alpha);
 mu_pup ~ normal(0.0, sigma_pup[1]);
 eta_pup ~ normal(0.0, sigma_pup[2]);
 tau ~ gamma(2.0, 1.0);
 sigma2 ~ gamma(1.0, prior_rate*tau);
 for (i in 1:n_ind) {
  z[i] ~ normal(0.0, exp(eta + eta_pup[i]));
 }
 for(i in 1:n_obs_tot){
  Y[i] ~ normal(drift_rate[ID[i], DAY[i]], sigma_error[i]);
 }
}
```

In effect, this model provides simultaneous smoothing of all drift rate curves (Yang *et al.*, 2016). We tried other approaches, included splines (Crainiceanu *et al.*, 2005) and Hidden Markov Models, but obtained similar results (not shown). We chose functional data analysis using the method of (Yang *et al.*, 2016) because this model is easy to implement and fast to estimate with Stan.

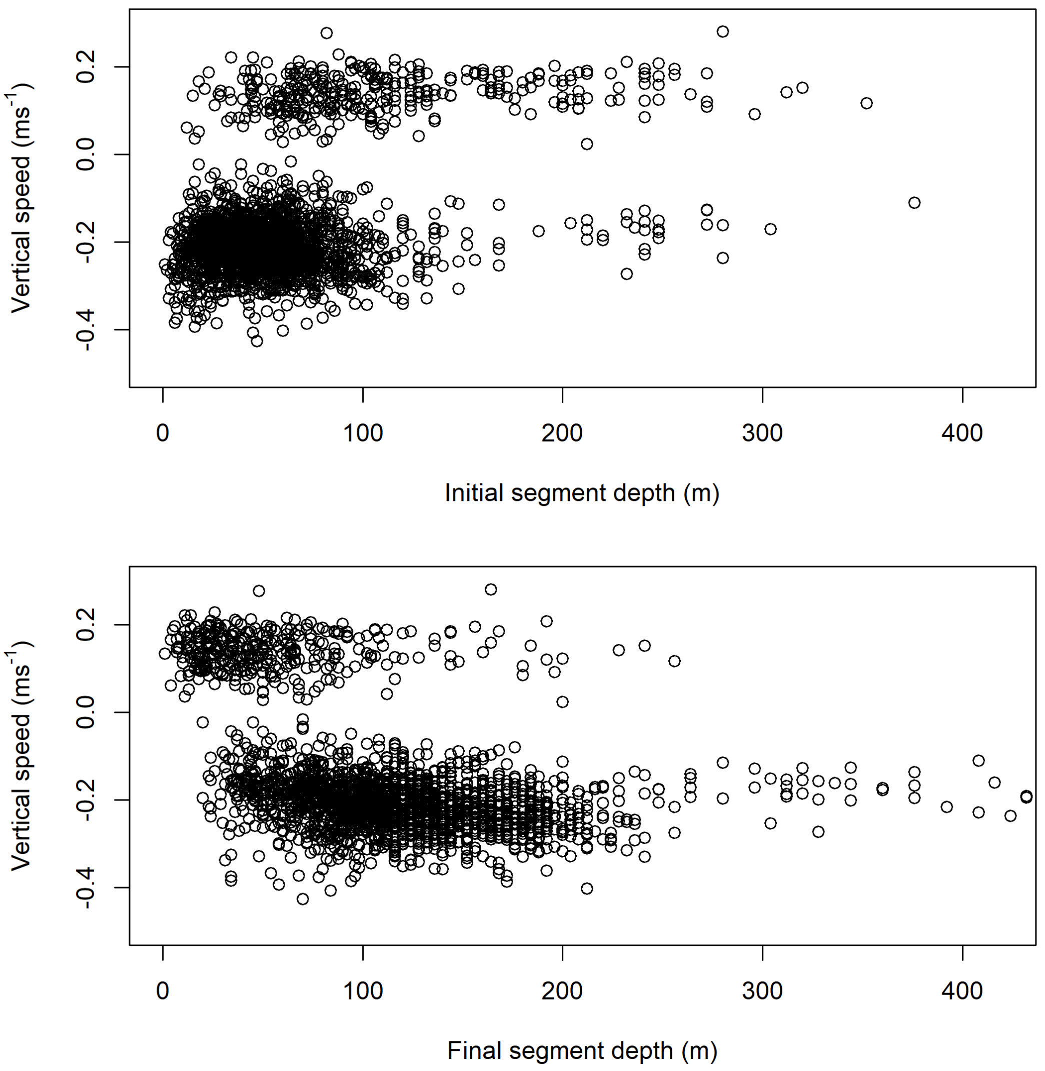

Figure S7. Vertical speeds of retained drift rates after the full step-wise filtering process, plotted in relation to (a) the initial depth of a segment, and (b) the final depth of a segment.

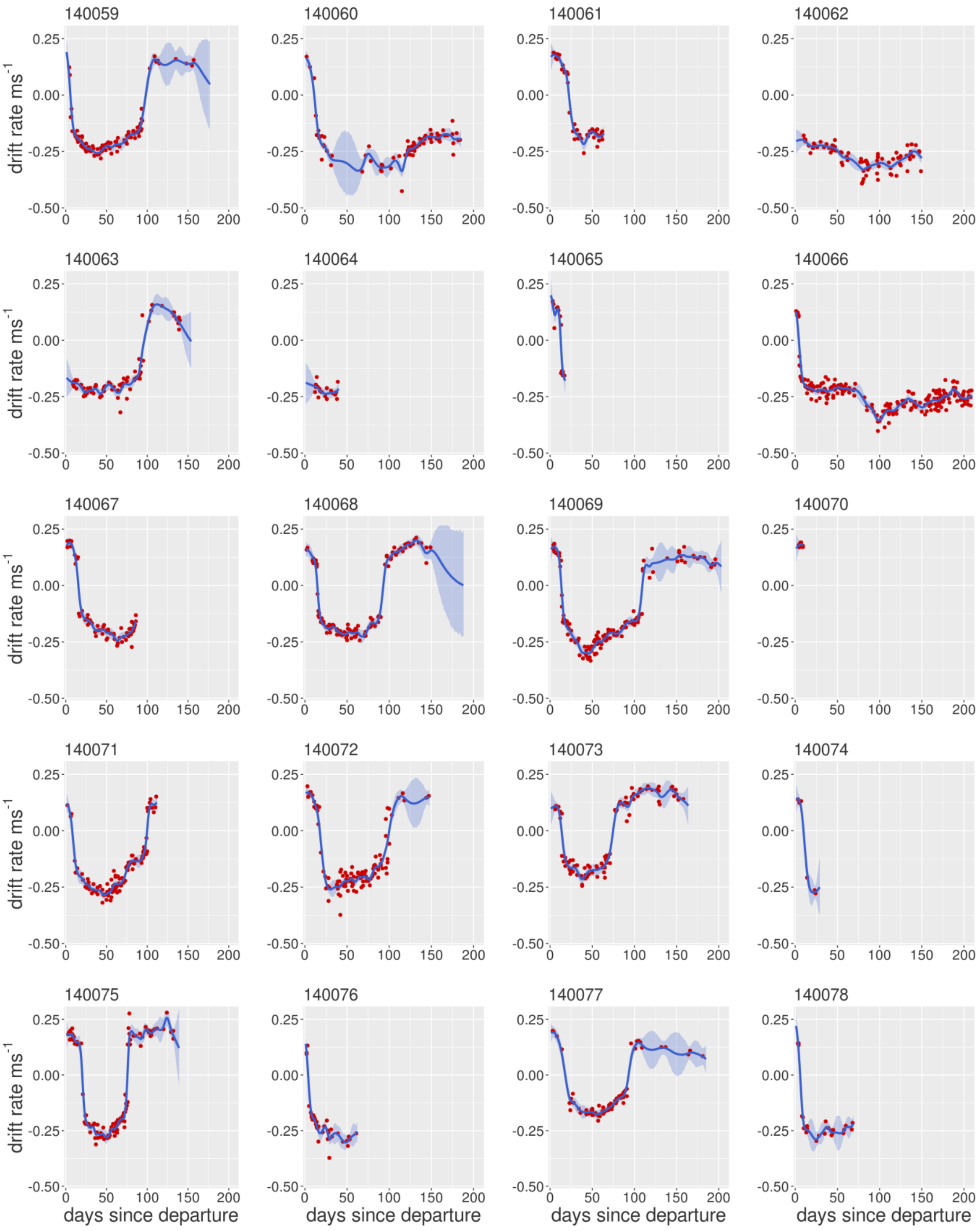

Figure S8. Predicted daily drift rates (blue line) with associated 95% confidence intervals (shaded blue) with day since departure for each of the juvenile southern elephant seals. Raw drift rates are indicated in red.

S.4 Investigations into potential sampling biases

Not all dives recorded by the DSA devices were transmitted (Cox *et al.*, 2018), and the number of dives received per day from an individual may vary with behaviour (e.g. surfacing intervals). As such, to assess potential sources of bias in our dataset that may impact any inferences made, we checked for differences in sampling rates both between surviving and dying individuals and through time using linear mixed effects model from the R package nlme (Pinheiro and Bates, 2014). Because eight out of nine of the individuals that died did so during the outward portion of their first trip at sea, all data were filtered to retain only samples taken before the distal point of the first trip of each individual. Models were fitted with a random intercept for each individual, and model selection was performed using AIC values via maximum likelihood estimation (Zuur *et al.*, 2009). To obtain parameter estimates, the final model was refitted using restricted maximum likelihood (REML).

We found no effect of survival outcome on daily sampling rates, or in an interaction with day since departure. However, an increase in sampling rate was observed with day since departure ($p < 0.001$, $\Delta AIC > 2$; Figure S9). The effect of this was small (parameter estimate = 0.009, marginal $r^2 = 0.01$; Nakagawa and Schielzeth 2013), such that at the two extremes of the analytical period (day 0 and day 110), the number of samples obtained per day would have varied by less than 1 dive (i.e. day 1 = 5.21 dives, day 110 = 6.20 dives). Individual variability in sampling rates was apparent but low (Figures S9, S10 & S11). The standard deviation around the random intercept was less than 1 dive per day, and accounted for 15.8% of variation in the number of samples obtained per day (assessed by calculating the difference in conditional and marginal r^2 values from the MuMin package in R; Nakagawa and Schielzeth 2013; Barton 2015).

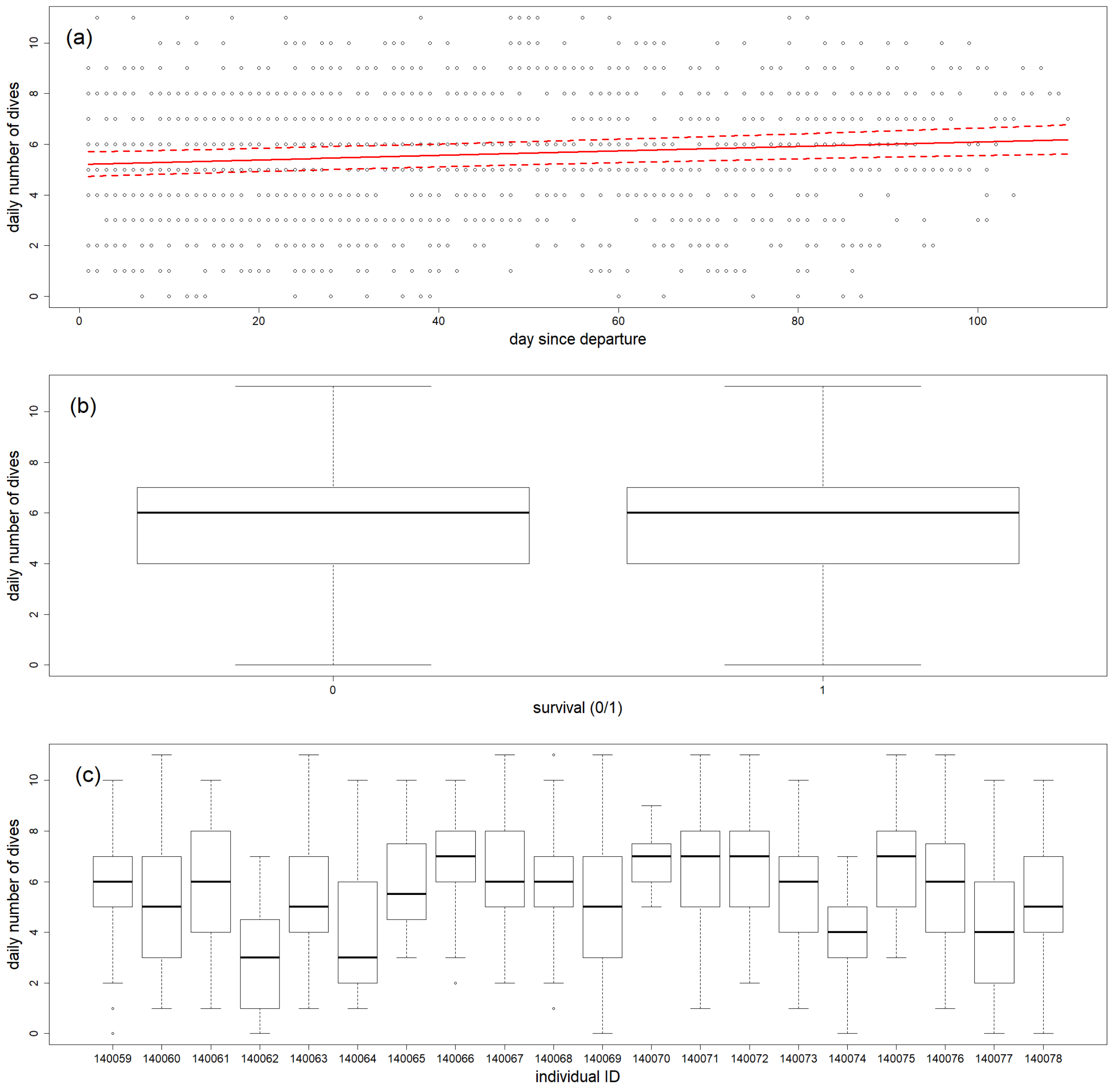

Figure S9. Summary plots of the daily number of dives sampled. From top to bottom: (a) with days since departure (red solid lines show bootstrapped means from a fitted linear mixed effects models with 95% confidence intervals shown by dashed red lines), (b) with survival outcome (0 = died, 1 = survived) and (c) averages by individual.

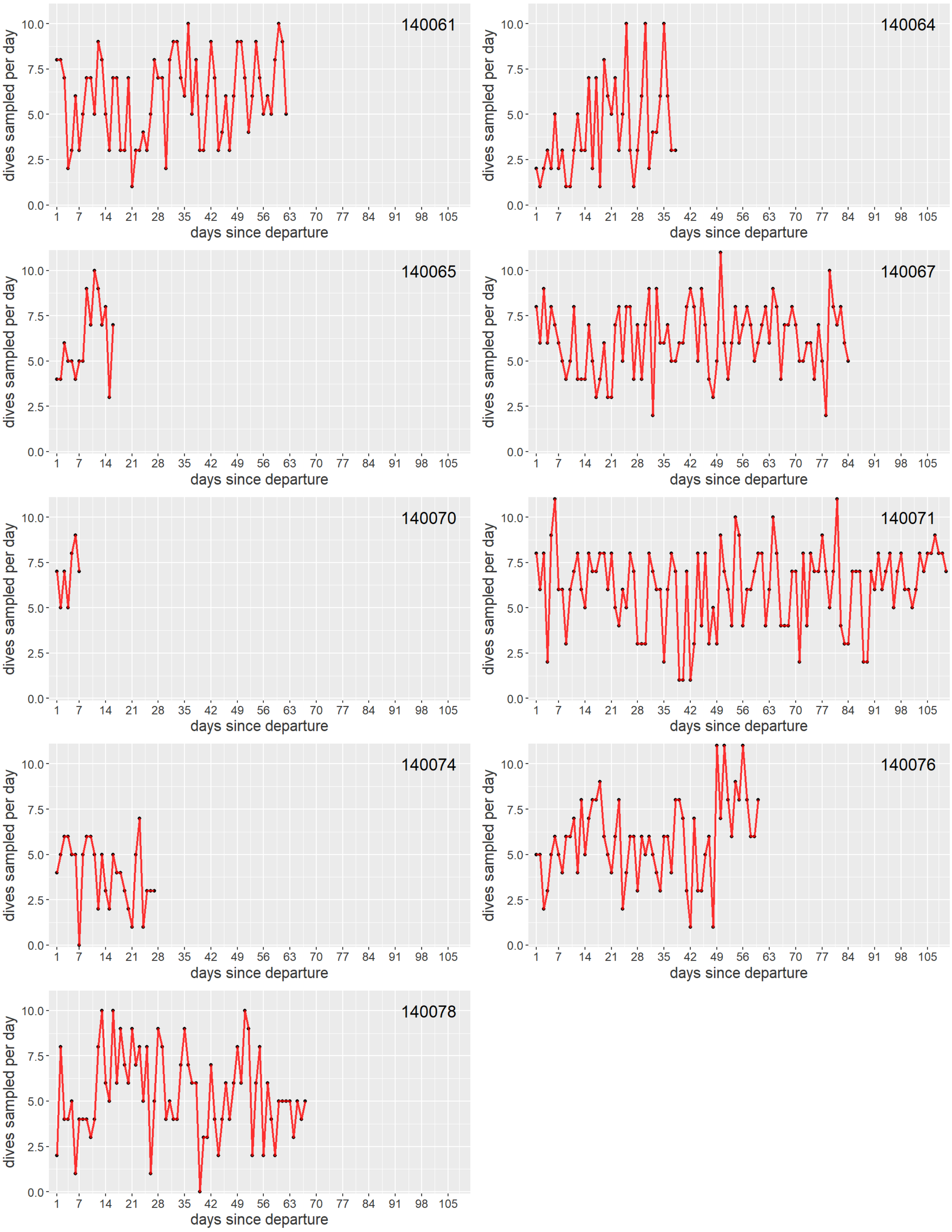

Figure S10. The daily number of dives sampled per individual that died (for plots of individuals that survived see Figure S11).

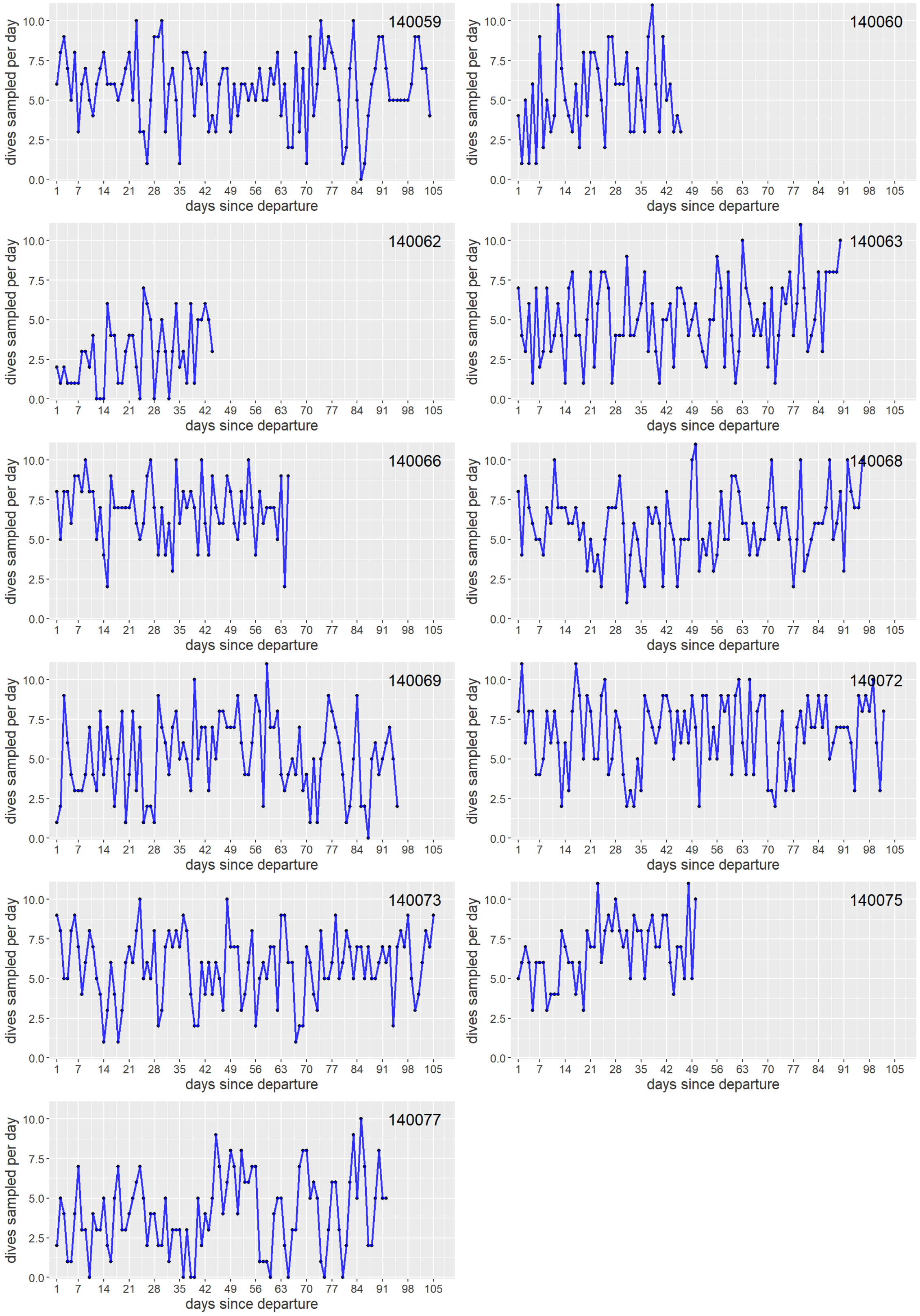

Figure S11. The daily number of dives sampled per individual that survived (for plots of individuals that died see Figure S10).

S.5 Broad scale spatial distributions of surviving and non-surviving individuals

Broad-scale spatial patterns in the distributions of the two (dead and surviving) groups were investigated to ensure comparability of behavioural indices and assess large scale patterns in space use. The 25%, 50%, 75% and 90% utilisation distributions (UDs) of the filtered SSM hourly locations were estimated using kernel density analysis via the R package 'adehabitatHR' (Calenge, 2014). Overlap between the spatial extents of the two groups was then estimated via Bhattacharyya's affinity (BA; Bhattacharyya 1943; Fieberg and Kochanny 2005). A randomisation technique was used to test the null hypothesis that there was no difference in the spatial distribution of those individuals that died and those that survived. If the null hypothesis is true, overlap magnitude between the two groups at each kernel level should not substantially differ from that calculated as if survival outcome were randomly assigned. As such, survival outcome for each individual was randomly assigned keeping the same ratio as that observed across the sample (i.e. nine mortalities and 11 survivals). A null distribution of BA values was then generated from 1000 randomisations of the dataset. Because the amount of data collected from the surviving group was much greater than that from the dead group, at each randomisation, these data were filtered to exclude all locations obtained after the time since departure at which a randomly matched dead individual died (with all dead individuals being matched at least once, and two twice). The observed distributions of the two groups were considered different if the maximum observed overlap (BA value) between the two was smaller than all 1000 randomly generated overlaps (Breed *et al.*, 2006).

Spatial overlap between the broad scale distributions of the two groups was strong (Figure 12). For all randomisation tests, at the four UD levels (25%, 505, 75% and 95%), the observed overlap was never less than that generated by random assignment of survival outcome (Figure 12).

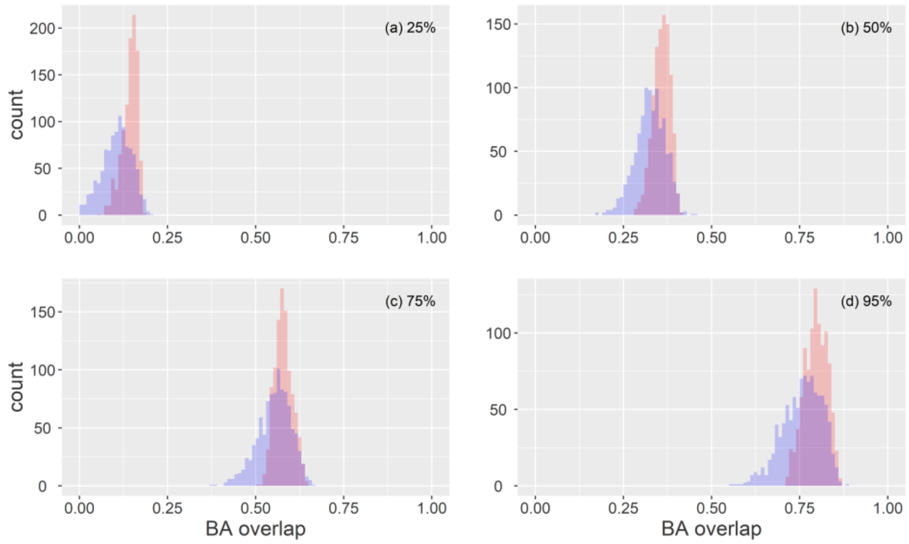

Figure 12. Distributions of Bhattacharyya's affinity (BA) for observed (red) and randomized (blue) datasets. Substantial overlap in the observed and randomized distributions at each level indicates no spatial segregation between the two groups (died and survived). Note that due to the scaling of BA values, comparisons should not be made between levels (e.g. 25% kernel utilisation density (KUD) values are not comparable to those from 75% KUDs).

S.6 Survival analysis with a regularized horseshoe prior

We analysed survival data on juvenile southern elephant seals with time-to-event methods. These methods rely on the modelling of the time until an event may happen or not, and they can use all the information in the data by including individuals for which survival times are censored (i.e. individuals that were still alive at the end of the study and whose time of death is thus unknown).

Parametric time-to-event modelling focuses on estimating hazards (i.e. the instantaneous probabilities of death over a specified period of time). For juvenile southern elephant seals, we modelled survival time (on a log scale) with a normal distribution. This choice equates to a non-monotonic hazard, which is plausible, as after leaving Kerguelen Islands, mortality hazard can be expected to increase after weaning (as energy capital from maternal provisioning depletes), and then decrease as pups learn to forage. T_i denotes the survival time of pup i:

$$log(T_i)\ N(\lambda_i, \sigma_{residual})$$

$$\lambda_i = \beta_0 + \sum_{j=1}^{p} \beta_j x_{ij}$$

where p denotes the number of covariates (features) thought to influence survival time. The regression coefficients β_j (excluding the intercept β_0) were modelled with a regularized horseshoe prior (Piironen and Vehtari, 2017). This prior belongs to the class of global-local shrinkage priors (Polson and Scott, 2010).

$$\beta_j \vee, \sigma^{global}, \sigma_j^{local}\ N\left(0, \sigma_j^{local}\right)$$

The overall global scale parameter σ^{global} shrinks, *a priori*, all coefficients β_j to 0, while the local scale parameters σ_j^{local} allow individual coefficients to escape this pull towards 0 when there is a signal in the data indicating the corresponding β_j is non-zero. To induce a horseshoe prior (Carvalho *et al.*, 2010), half-Cauchy priors are put on global and local scales:

$$+(0, \sigma_{residual})$$

$$\sigma^{global}\ C \quad forallj \in [1:p], \sigma_j^{local}\ C^{+\left(0, \sigma^{global}\right)}$$

Global-local shrinkage priors translate an assumption of sparsity, where most regression coefficients are expected to be zero, and only a few ones may be non-zero. In the words of Polson and Scott (2010), "strong global shrinkage handles the noise, the local σ_j^{local}'s act to detect the signals [...]." Shrinkage priors thus perform variable selection by simultaneously identifying and estimating non-zero β_j's. Carvalho *et al.*, (2010) further showed that the posterior means of β_j, obtained from a horseshoe prior, were akin to model-averaged estimates in linear models. Piironen and Vehtari (2017) discussed shortcomings of the horseshoe prior, including the choice of the global scale to control the *a priori* number of non-zero coefficients. They proposed a regularized horseshoe prior that addresses these

shortcomings, and allows the specification, *a priori*, of the number of expected non-zero regression coefficients, which we set to 10.

We fitted this model with Stan v.2.17.3 (Carpenter et al. 2017) using weakly informative priors on all parameters save the regression coefficients β_j, for which we used a regularized horseshoe prior. We ran four chains with a warmup of 1,000 iterations, and 1,000 more iterations for inference. All parameters appeared to have converged, as assessed by the Gelman-Brook-Rubin statistics (all $\hat{R} < 1.1$). The Stan code is given below:

```
/*  Variable naming:
  n_ind     = number of individuals
  n_cov    = number of covariates
  sigma    = residual scale parameter
  X          = covariates, including sex (standardized)
  TIME    = time to event data (log scale)
  SURVIVAL  = survival indicator
 */
 data {
   int<lower = 0> n_ind;
   vector<lower = 0>[n_ind] TIME;
   int<lower = 0, upper = 1> SURVIVAL[n_ind];
   int<lower = 0> n_cov;
   matrix[n_ind, n_cov] X_obs;
   real<lower = 1> nu_global;            // degrees of freedom for the half -t priors for global scale
   real<lower = 1> nu_local;             // degrees of freedom for the half -t priors for local scales
   real<lower = 0> prior_scale_for_mu;    // depends on log TIME scale
   real<lower = 0> prior_scale_for_sigma; // depends on log TIME scale
   real<lower = 0> prior_scale_for_global;// depends on how many features a priori
}

 parameters {
   real log_sigma;
   vector[n_cov] unscaled_beta;
   real unscaled_mu;
   vector<lower=0>[n_cov] local_sq;
   vector<lower=0>[n_cov] aux_local;
   real<lower=0> aux_global;
   real<lower=0> global_sq;
 }

 transformed parameters {
   real mu;
   real sigma;
   real global;
   vector[n_cov] beta;
   vector[n_cov] local;
   vector[n_ind] lambda;
   // intercept
   mu = unscaled_mu * prior_scale_for_mu;
   // residual std.  dev.
   sigma = exp(log_sigma) * prior_scale_for_sigma;
   // hierarchical horseshoe (Piironen & Vehtari 2017)
   global = aux_global * sqrt(global_sq) * prior_scale_for_global * sigma;
   local = aux_local .* sqrt(local_sq);
   // regression coefficients
   beta = unscaled_beta .* local * global;
```

```
  // linear predictors
  lambda = rep_vector(mu, n_ind) + X_obs * beta;
}

model {
  // normal regression of log TIME: hazard function is non-monotonic
  for (i in 1:n_ind) {
    if(SURVIVAL[i] == 0) { // observed
     target += normal_lpdf(TIME[i]| lambda[i], sigma);
    }
    else { // censored
      target += normal_lccdf(TIME[i]| lambda[i], sigma);
    }
  }

  // priors
  log_sigma ~ normal(0.0, 1.0);
  unscaled_beta ~ normal(0.0, 1.0);
  unscaled_mu ~ normal(0.0, 1.0);
  // horseshoe: scale mixture of normal distributions
  aux_local ~ normal(0.0, 1.0);
  local_sq ~ inv_gamma(0.5 * nu_local, 0.5 * nu_local);
  aux_global ~ normal(0.0, 1.0);
  global_sq ~ inv_gamma(0.5 * nu_global, 0.5 * nu_global);
}

generated quantities{
  vector[n_ind] y_rep;
  real rmse;
  for(i in 1:n_ind) {
    y_rep[i] = normal_rng(lambda[i], sigma);
  }
  rmse = sum(square(TIME - y_rep));
}
```

We assessed model fit by plotting the Kaplan-Meier survival estimates against the survival curve predicted from the fitted model (Figure S13).

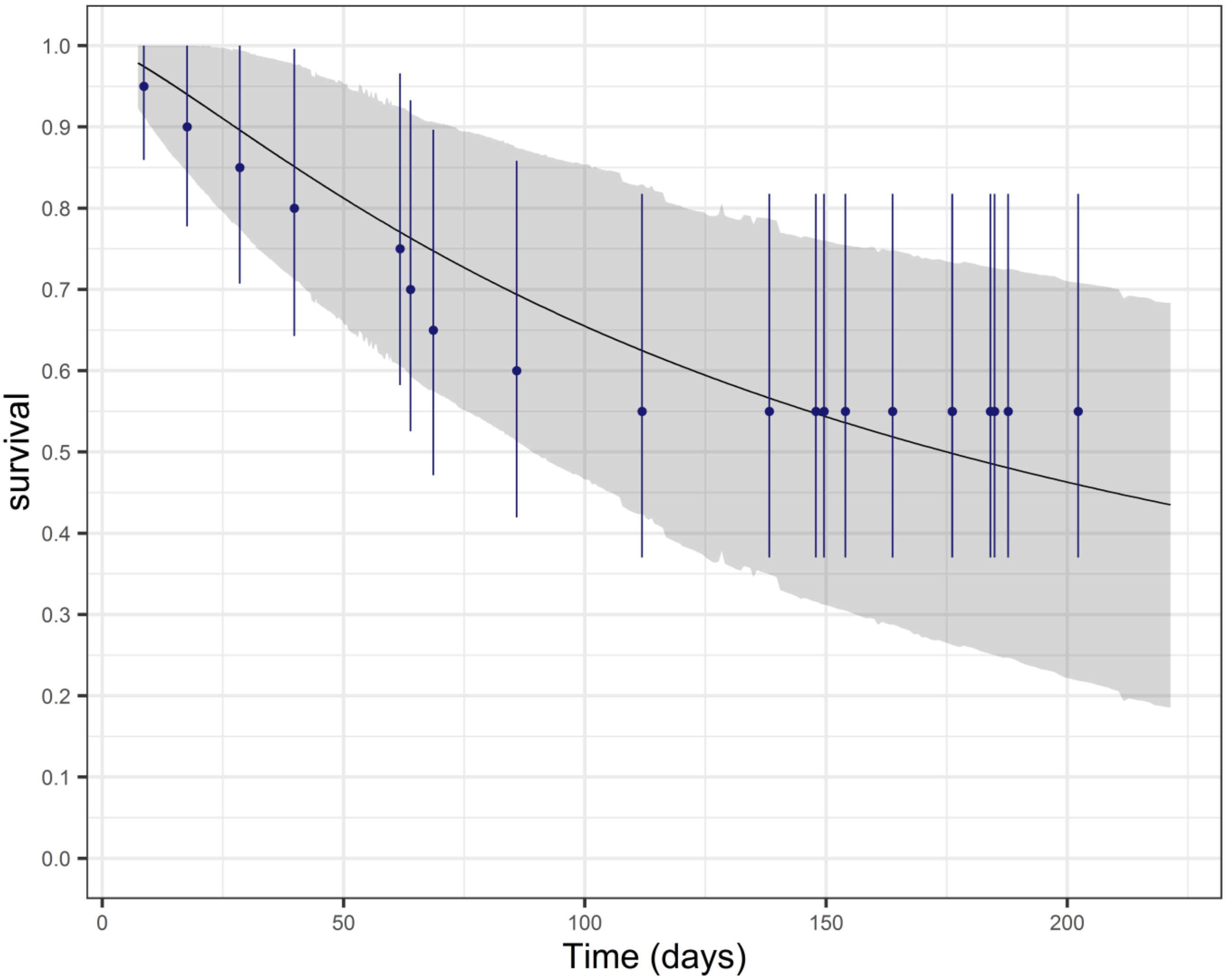

Figure S13. Assessing survival model fit, survival rate is plotted against time in days. The survival curve predicted by the model is in black and the 80% credible interval in light blue. Kaplan-Meier estimates are in dark blue. Model fit was deemed acceptable.

S.7 Retrospective power analysis of the survival analysis using a regularized horseshoe prior

We conducted a retrospective power analysis to investigate the true positive (TPR) and false positive (FPR) rates of the survival model with regularized horseshoe prior (the third applied method (and second shrinkage method) of the study; Piironen and Vehtari, 2017). TPR is the long-term expected frequency with which the method picks true signals, and the FPR is the long-term expected frequency with which the method wrongly selects a null effect.

The simulation setting was the following: sample size was set to 20 individuals, the number of candidate covariates (features) was set to 50, and the number of non-zero effects (true signals) varied between 0 and 15 by increment of 5. The regularized horseshoe prior was calibrated for an expected numbers of 10 active features. The effect size of true signals was generated from a normal distribution $\mathcal{N}(\log(1.1), 0.02)$. We thereby assumed that the average effect size was an increase (or decrease) of 10% in average longevity, with a prior range between 5% and 15%. This figure roughly matches the estimated effect sizes in the actual analysis of the true longevity data from elephant seal pups. We then simulated data according to the following processes:

The (log) duration (in days) of a pup's first trip at sea z was generated from a normal distribution $z \sim \mathcal{N}(\mu_{\text{trip}}, \sigma_{\text{trip}})$ with parameters $(\mu_{\text{trip}}, \sigma_{\text{trip}})$ generated from a multivariate normal distribution:

$$\begin{bmatrix} \mu_{\text{trip}} \\ \sigma_{trip} \end{bmatrix} \sim \mathcal{MNV}(\begin{bmatrix} 5.13 \\ 0.15 \end{bmatrix}, \begin{bmatrix} 0.05^2 & 0.00 \\ 0.00 & 0.04^2 \end{bmatrix})$$

The hyperparameter values were taken from the estimated values of the true data on elephant seal pups. The (log) longevity (in days) of a pup y was generated from a normal distribution $y \sim \mathcal{N}(\mu_{\varphi}, \sigma_{\varphi})$ with parameters $(\mu_{\varphi}, \sigma_{\varphi})$ generated from a multivariate normal distribution:

$$\begin{bmatrix} \mu_{\varphi} \\ \sigma_{\varphi} \end{bmatrix} \sim \mathcal{MNV}(\begin{bmatrix} 5.22 \\ 1.49 \end{bmatrix}, \begin{bmatrix} 0.50^2 & 0.55 \times 0.50 \times 0.51 \\ 0.55 \times 0.50 \times 0.51 & 0.51^2 \end{bmatrix})$$

The hyperparameter values were taken from the estimated values of the true data on elephant seal pups.

The observed data for a pup was the minimum of (y, z): if $y > z$, the pup survived its first trip at sea, and if $y < z$ it died during its first trip at sea. The modelling of both longevity and trip duration is needed to mimic the censoring mechanism that is assumed in our analysis of the true data.

We simulated 100 datasets for each scenario and analysed each simulated data with the same model that was used for the true data. All analysed were carried out with software Stan (Carpenter *et al.*, 2017). Results are summarized in Figure S14.

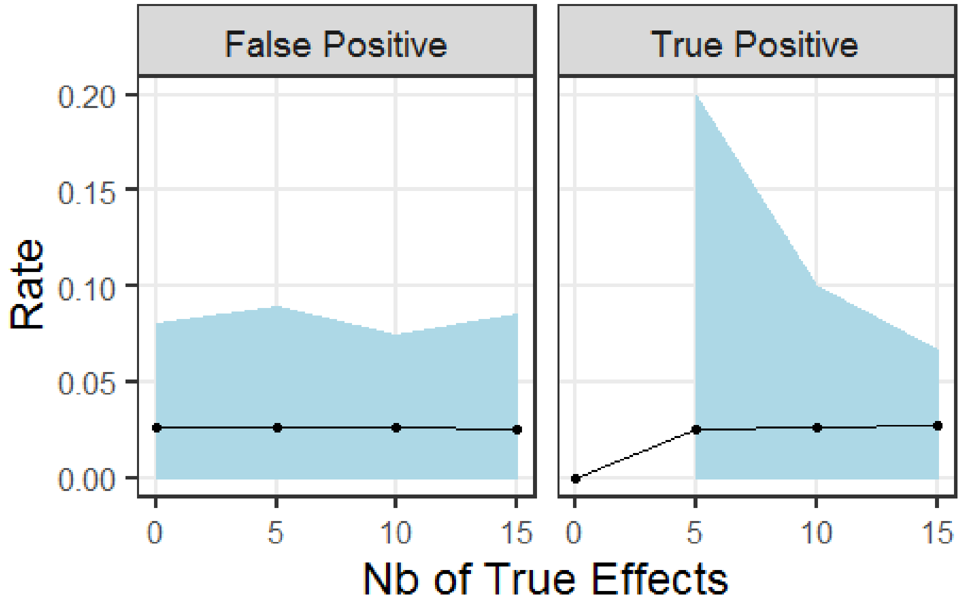

Figure S14. False positive rates (left panel) and true positive rates (right panel) of the model with a regularized horseshoe prior.

For all scenarios, results were similar: FPR and TPR were roughly equal and around 2.5%. In other words, the probability for a feature picked up by the regularized horseshoe prior to be a true signal was 50%. Although this is low, this retrospective power analysis nevertheless shows how conservative our analysis was. It is extremely unlikely to pick up false signals, and it is also extremely unlikely to pick true ones among 50 candidates with the regularized horseshoe and a sample size of 20 data points. Thus this analysis is efficient in discarding irrelevant features, and for the few ones that survived the selective filter of the regularized horseshoe, the probability that they are true signals is 50%.

Applying these results to the true data means that this analysis is effective in discarding most of the candidate covariates as irrelevant, and the ones that are picked up are good candidates for further investigation. Hence our focus on a descriptive but robust analysis on the correlates of early life longevity in elephant seal pups from Kerguelen Islands.

R code snippet to simulate data:

```
sim_data <- function(n_obs, p = 0, n_cov = 50) {
# generate parameters
 theta_surv = mvtnorm::rmvnorm(1, c(5.22, 1.49), matrix(c(0.50*0.50, 0.55*0.50*0.51, 0.55*0.50*0.51, 0.51*0.51), nrow = 2, byrow = TRUE))
 theta_cens = mvtnorm::rmvnorm(1, c(5.13, 0.15), matrix(c(0.05*0.05, 0, 0, 0.04*0.04), nrow = 2, byrow = TRUE))
# features
 X_obs <- cbind(rep(1, n_obs), replicate(n_cov, rnorm(n_obs)))
 if(p != 0) {
# effect size is 10% increase/decrease compared to baseline with std.  dev chosen so that prior 99% CI  is 1.04:1.15
  beta = c(theta_surv[1, 1], (-1)^rbinom(p, 1, prob = 0.5)*rnorm(p, log(1.1), 0.05/qnorm(0.995)), rep(0, n_cov - p))
 }
 else { beta <- c(theta_surv[1, 1], rep(0, n_cov)) }
 linpred <- as.numeric(X_obs %*% beta)
 # lifetime
 y <- exp(rnorm(n = n_obs, linpred, theta_surv[1, 2]))
 # trip duration
 z <- exp(rnorm(n = n_obs, theta_cens[1, 1], theta_cens[1, 2]))
 data <- data.frame(surv_days = y,
          cens_days = z,
          censored = ifelse(y > z, 1, 0),
          event = ifelse(y > z, 0, 1),
          stringsAsFactors = FALSE
          ) %>%
  dplyr::mutate(status = ifelse(censored == 0, 'DECEASED', 'LIVING'), t = ifelse(y > z, z, y) )
 return(list(Y = data, X = X_obs[, -1], beta = beta[-1], theta = as.numeric(cbind(theta_surv, theta_cens))))
}
```

S.8 Visual comparisons of behaviours and encountered environmental conditions of non-surviving pups to those of grouped survivors

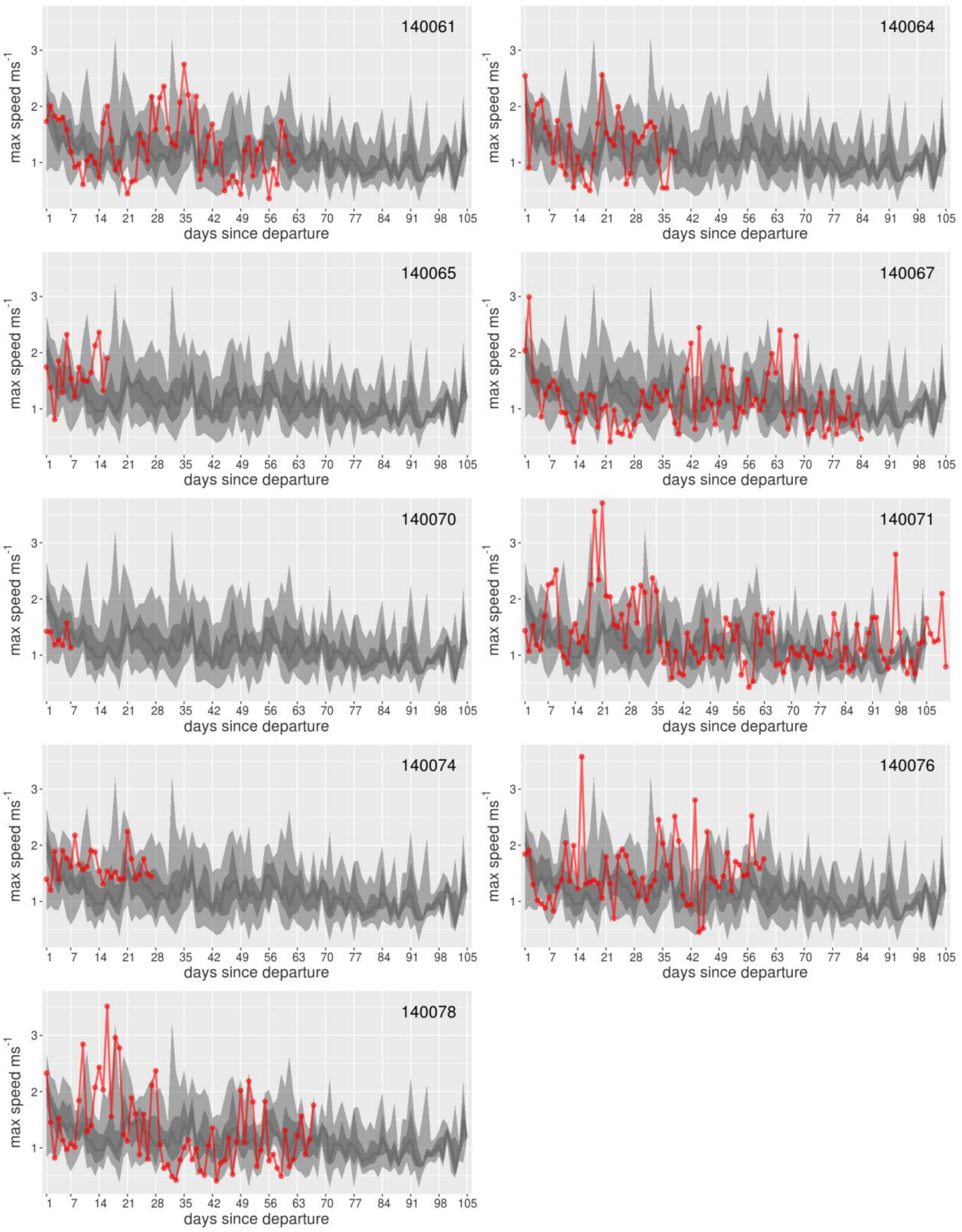

Figure S15. Time series plots of the daily maximum corrected speeds for each individual that died (for plots of individuals that survived see Figure S16). Large light grey bands represent the 2.5% to 97.5% quantiles of the survival datasets. Nested within this in darker grey are the 25 to 75% quantiles. The daily median of the survival data is represented by a dark grey line. The red line indicates the daily values of the individual identified in the top right-hand corner.

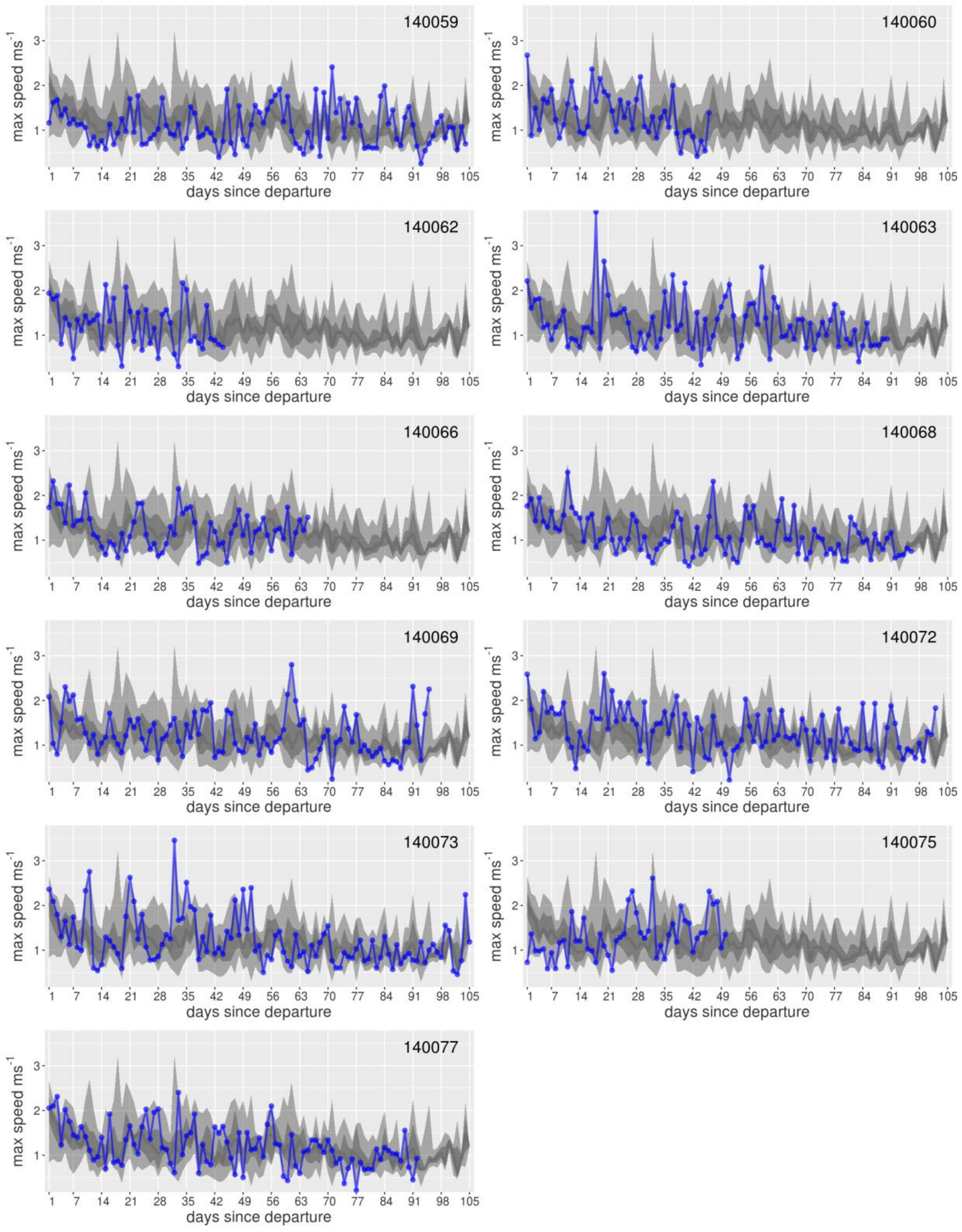

Figure S16. Time series plots of the daily maximum corrected speeds of each individual that survived (for plots of individuals that died see Figure S15). Large light grey bands represent the 2.5% to 97.5% quantiles of the survival datasets. Nested within this in darker grey are the 25 to 75% quantiles. The daily median of the survival data is represented by a dark grey line. The blue line indicates the daily values of the individual identified in the top right-hand corner.

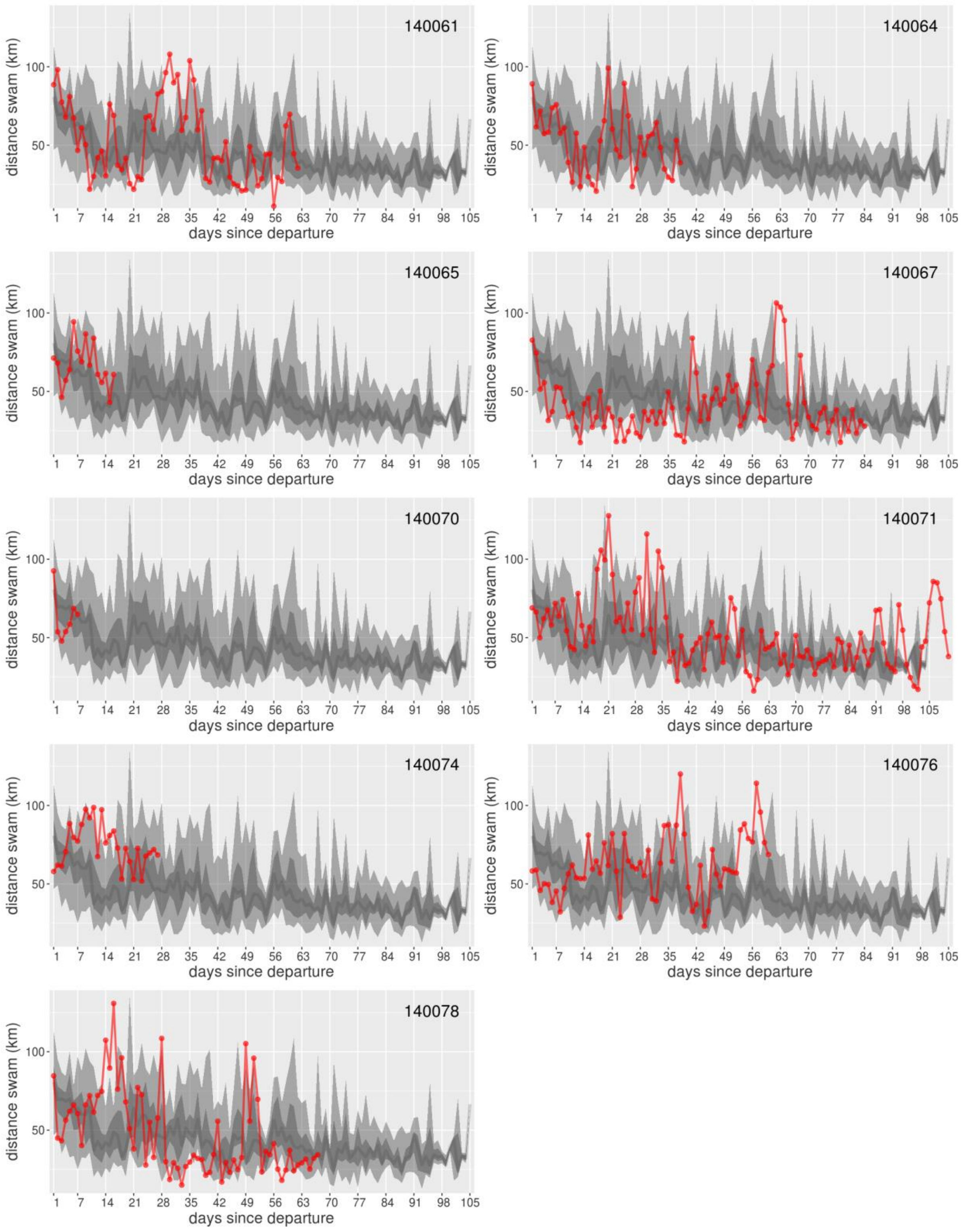

Figure S17. Time series plots of daily corrected distances swam by each individual that died (for plots of individuals that survived see Figure S18). Large light grey bands represent the 2.5% to 97.5% quantiles of the survival datasets. Nested within this in darker grey are the 25 to 75% quantiles. The daily median of the survival data is represented by a dark grey line. The red line indicates the daily values of the individual identified in the top right-hand corner.

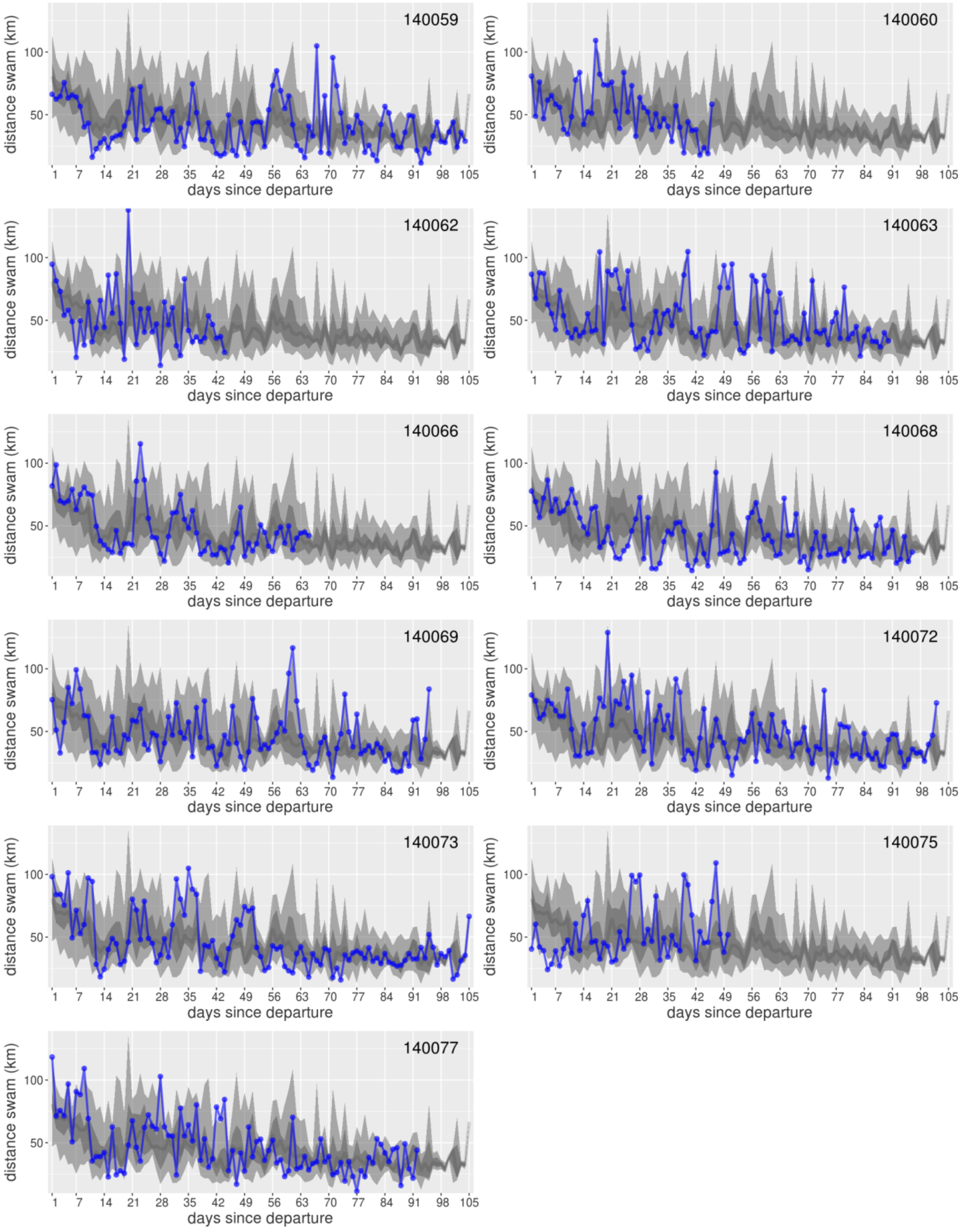

Figure S18. Time series plots of daily corrected distances swam by each individual that survived (for plots of individuals that died see Figure S17). Large light grey bands represent the 2.5% to 97.5% quantiles of the survival datasets. Nested within this in darker grey are the 25 to 75% quantiles. The daily median of the survival data is represented by a dark grey line. The blue line indicates the daily values of the individual identified in the top right-hand corner.

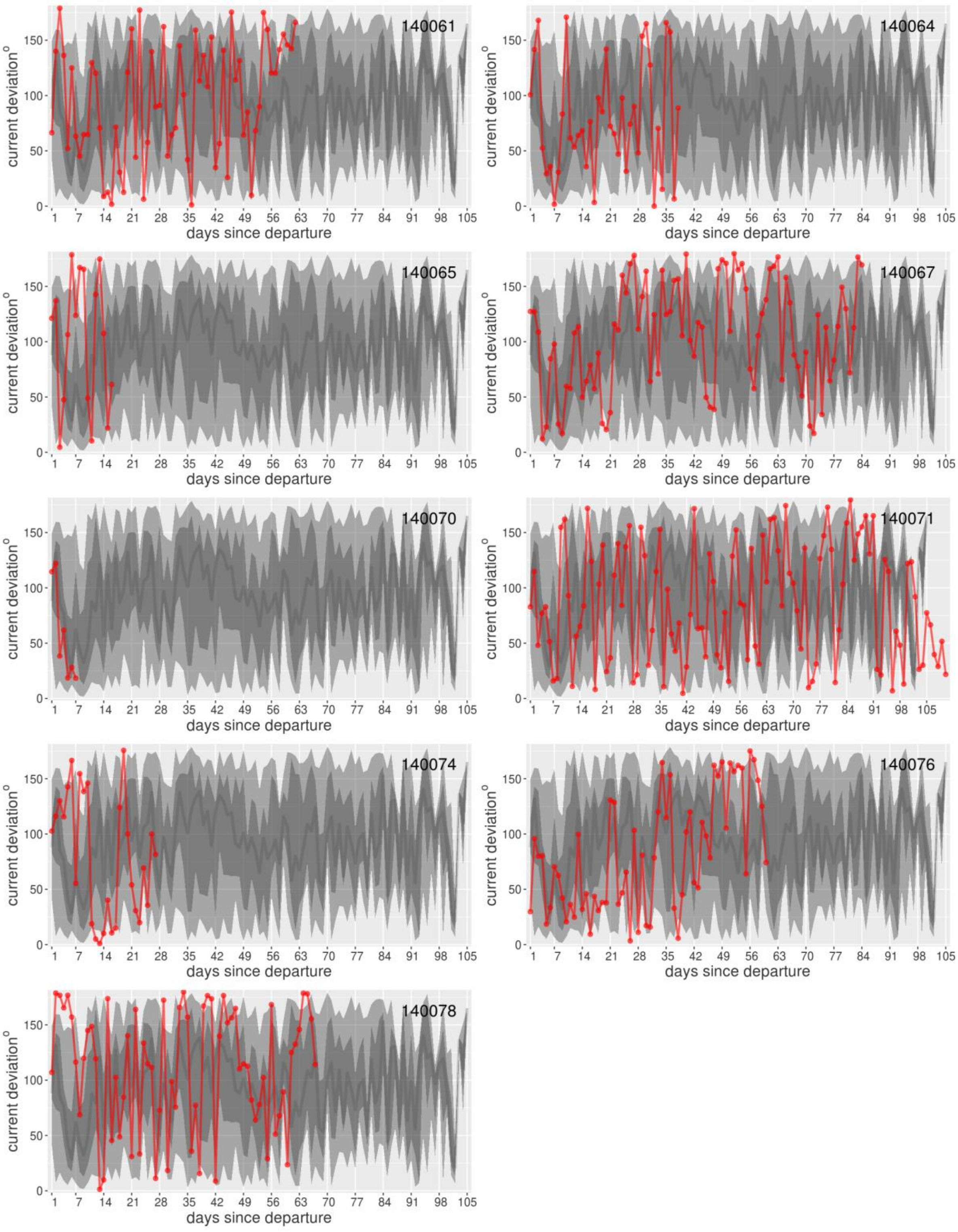

Figure S19. Time series plots of daily mean deviations from ocean currents by each individual that died (for plots of individuals that survived see Figure S20). Large light grey bands represent the 2.5% to 97.5% quantiles of the survival datasets. Nested within this in darker grey are the 25 to 75% quantiles. The daily median of the survival data is represented by a dark grey line. The red line indicates the daily values of the individual identified in the top right-hand corner.

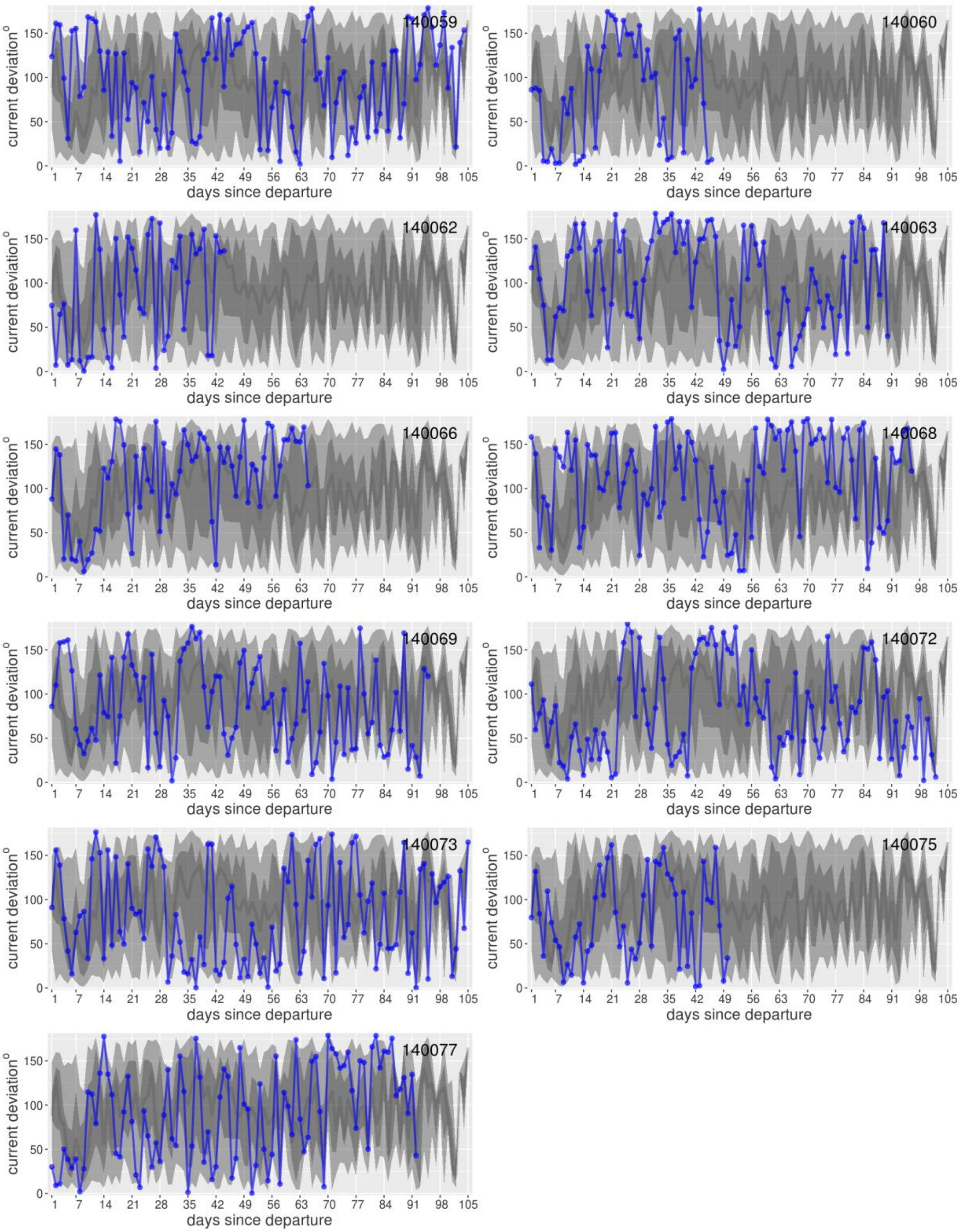

Figure S20. Time series plots of daily mean deviations from ocean currents by each individual that survived (for plots of individuals that died see Figure S19). Large light grey bands represent the 2.5% to 97.5% quantiles of the survival datasets. Nested within this in darker grey are the 25 to 75% quantiles. The daily median of the survival data is represented by a dark grey line. The blue line indicates the daily values of the individual identified in the top right-hand corner.

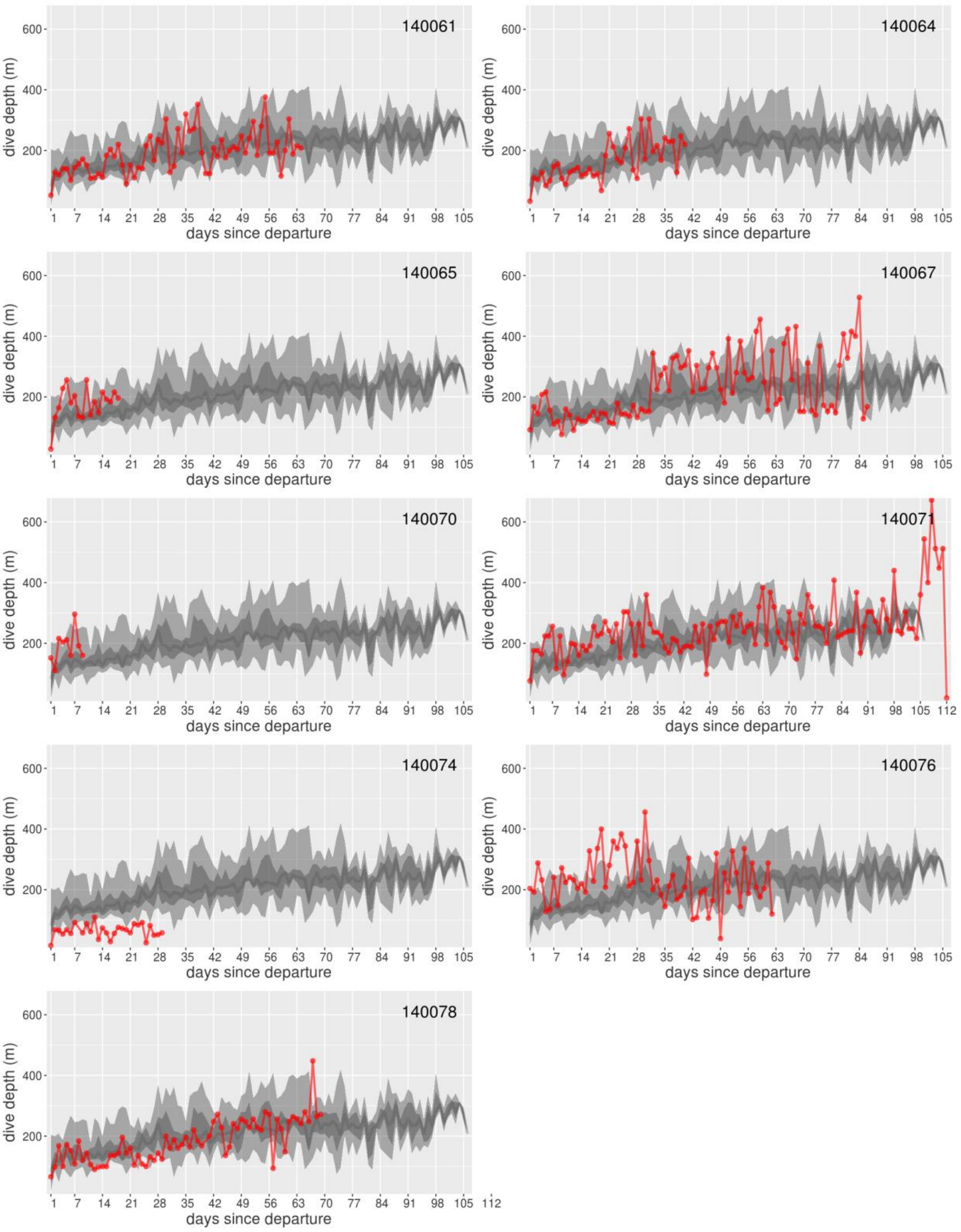

Figure S21. Time series plots of daily maximum dive depths for each individual that died (for plots of individuals that survived see Figure S22). Large light grey bands represent the 2.5% to 97.5% quantiles of the survival datasets. Nested within this in darker grey are the 25 to 75% quantiles. The daily median of the survival data is represented by a dark grey line. The red line indicates the daily values of the individual identified in the top right-hand corner.

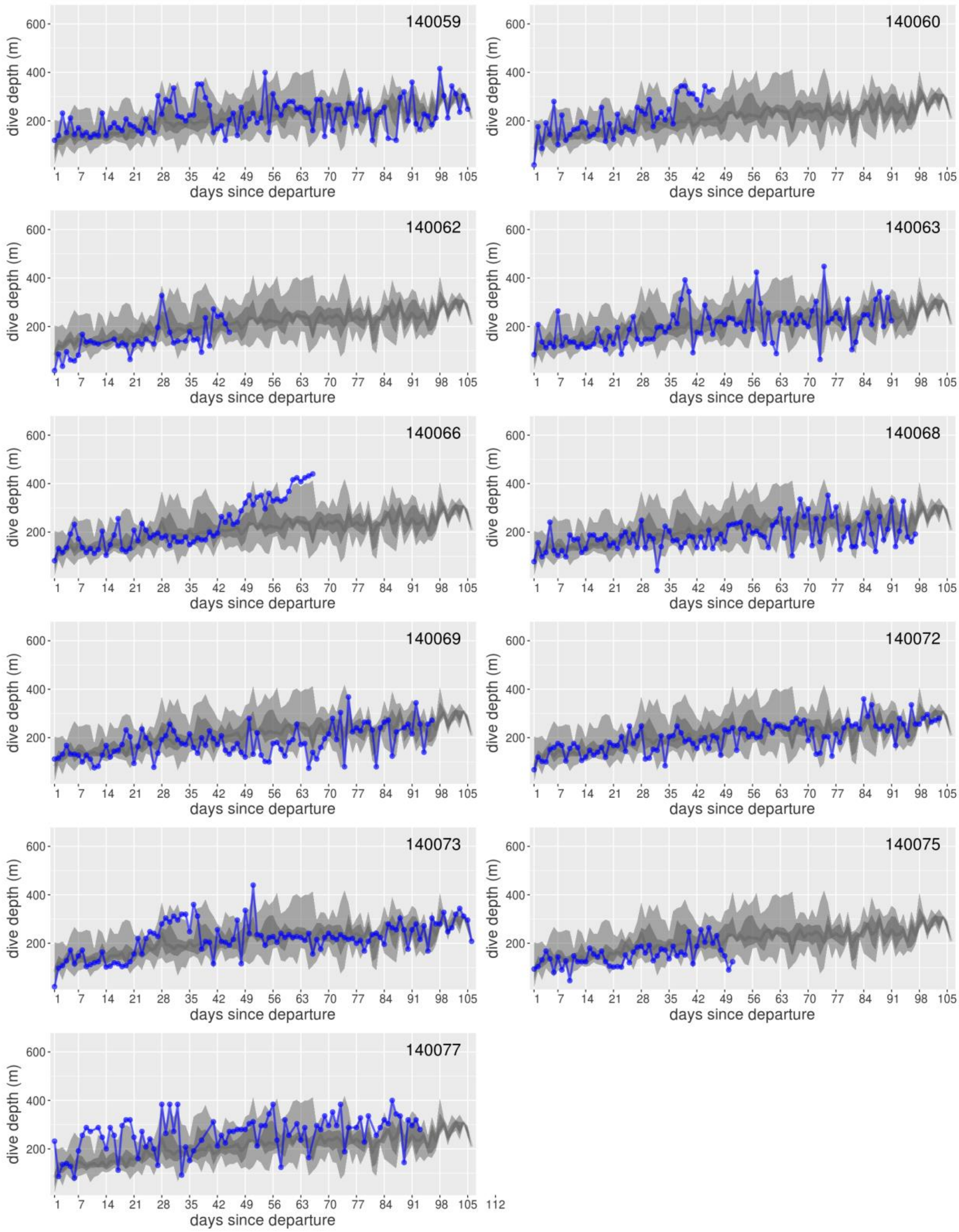

Figure S22. Time series plots of daily maximum dive depths for each individual that survived (for plots of individuals that died see Figure S21). Large light grey bands represent the 2.5% to 97.5% quantiles of the survival datasets. Nested within this in darker grey are the 25 to 75% quantiles. The daily median of the survival data is represented by a dark grey line. The blue line indicates the daily values of the individual identified in the top right-hand corner.

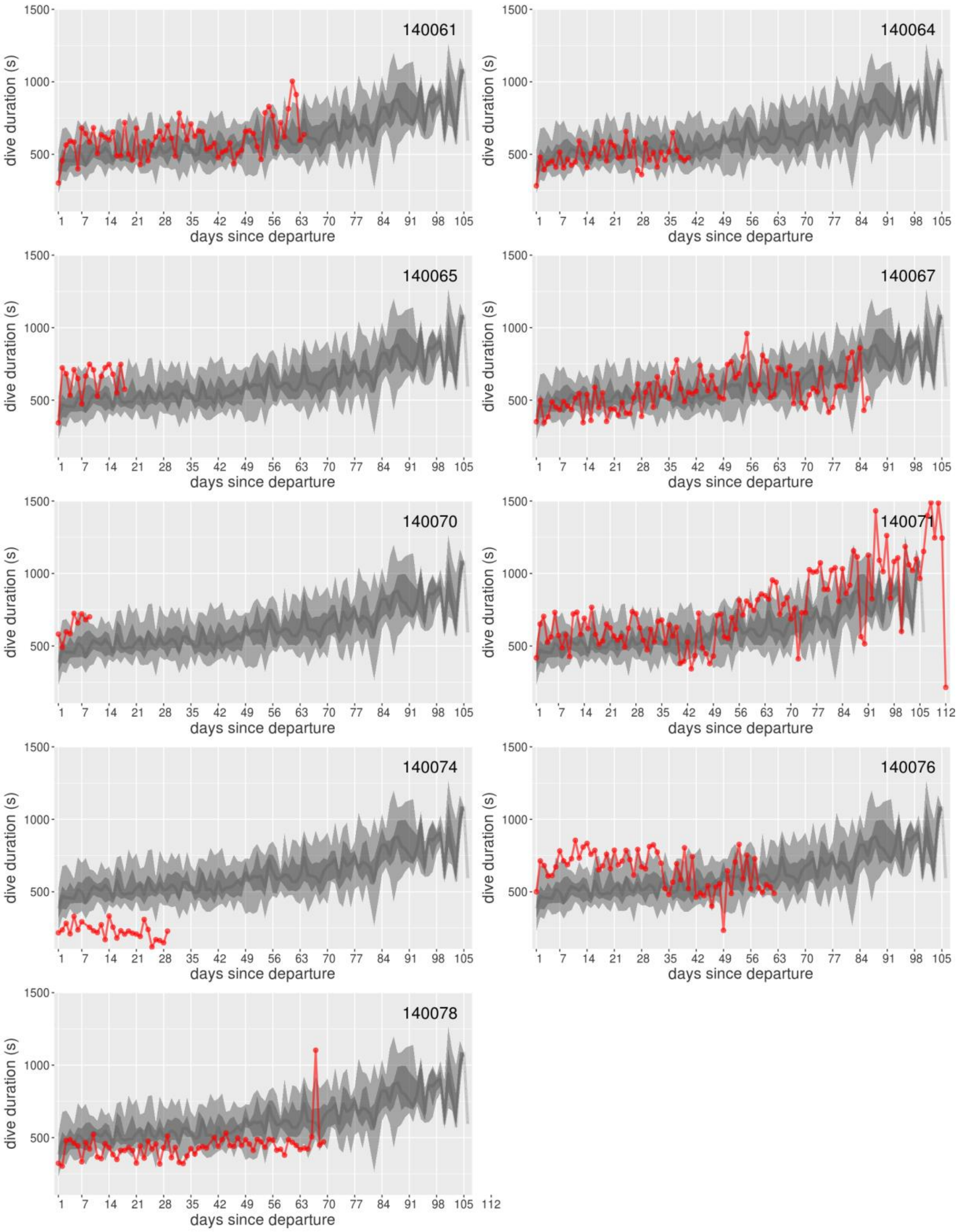

Figure S23. Time series plots of daily maximum dive durations for each individual that died (for plots of individuals that survived see Figure S24). Large light grey bands represent the 2.5% to 97.5% quantiles of the survival datasets. Nested within this in darker grey are the 25 to 75% quantiles. The daily median of the survival data is represented by a dark grey line. The red line indicates the daily values of the individual identified in the top right-hand corner.

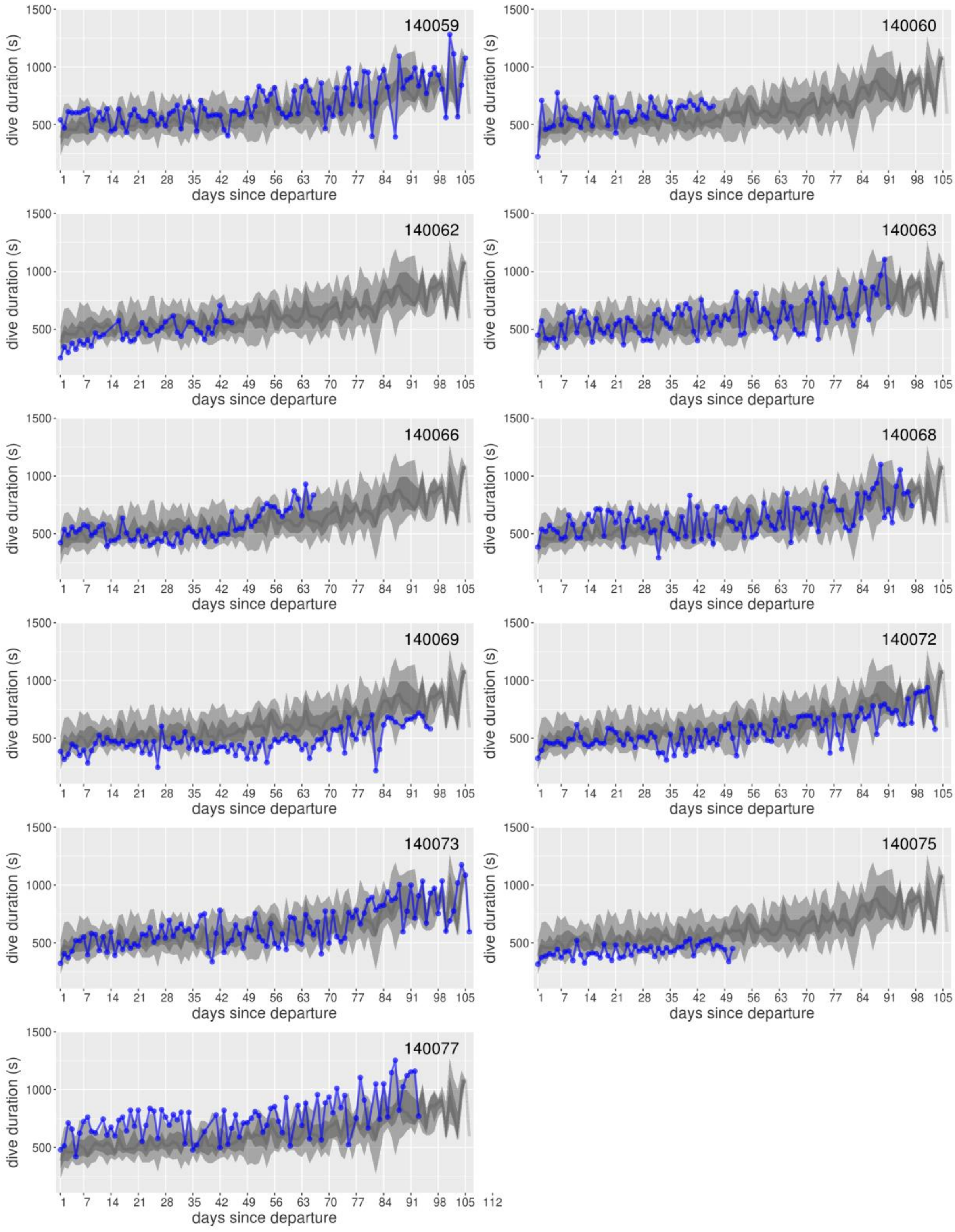

Figure S24. Time series plots of daily maximum dive durations for each individual that survived (for plots of individuals that died see Figure S23). Large light grey bands represent the 2.5% to 97.5% quantiles of the survival datasets. Nested within this in darker grey are the 25 to 75% quantiles. The daily median of the survival data is represented by a dark grey line. The blue line indicates the daily values of the individual identified in the top right-hand corner.

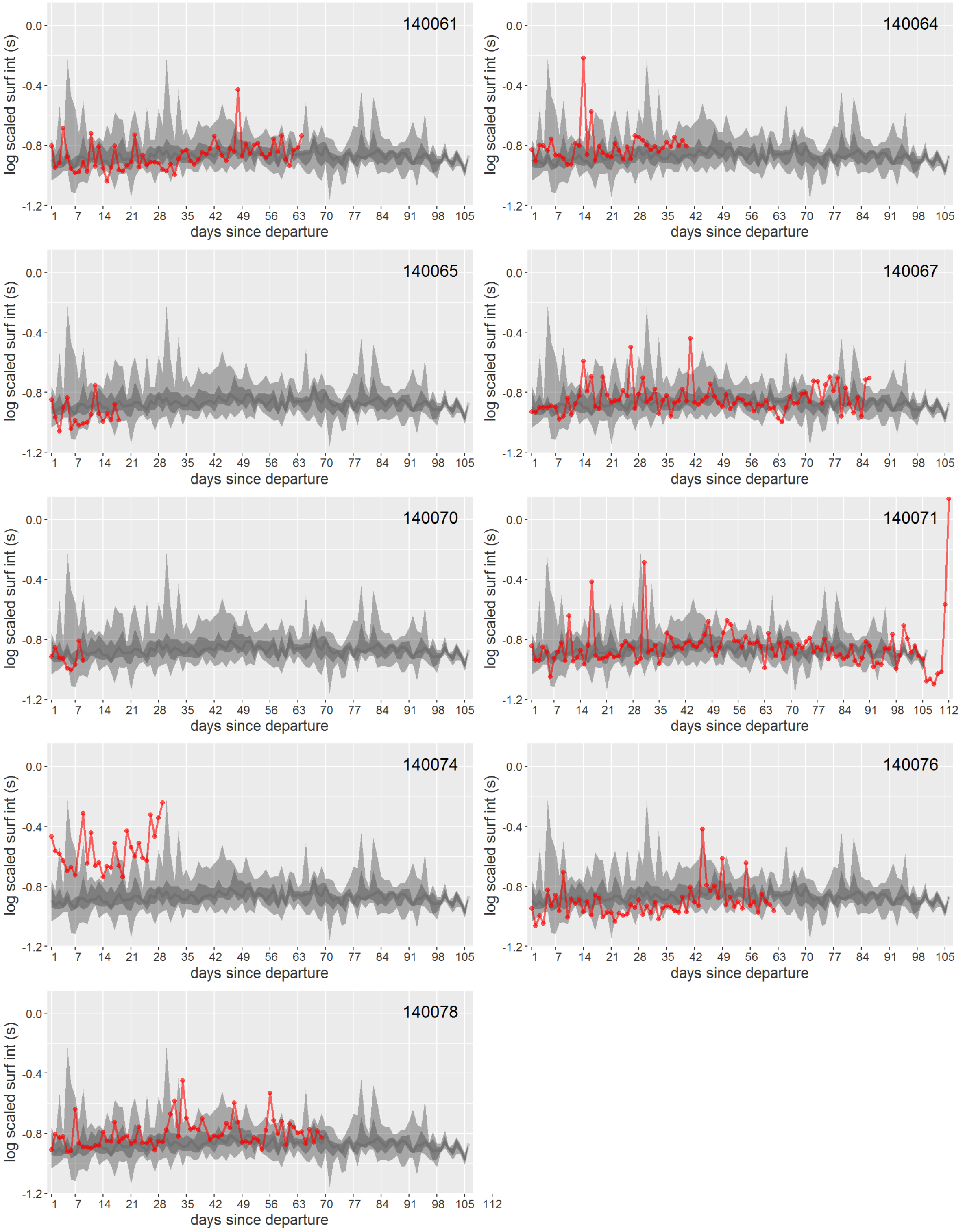

Figure S25. Time series plots of daily mean surface intervals (logged) for each individual that died (for plots of individuals that survived see Figure S26). Large light grey bands represent the 2.5% to 97.5% quantiles of the survival datasets. Nested within this in darker grey are the 25 to 75% quantiles. The daily median of the survival data is represented by a dark grey line. The red line indicates the daily values of the individual identified in the top right-hand corner.

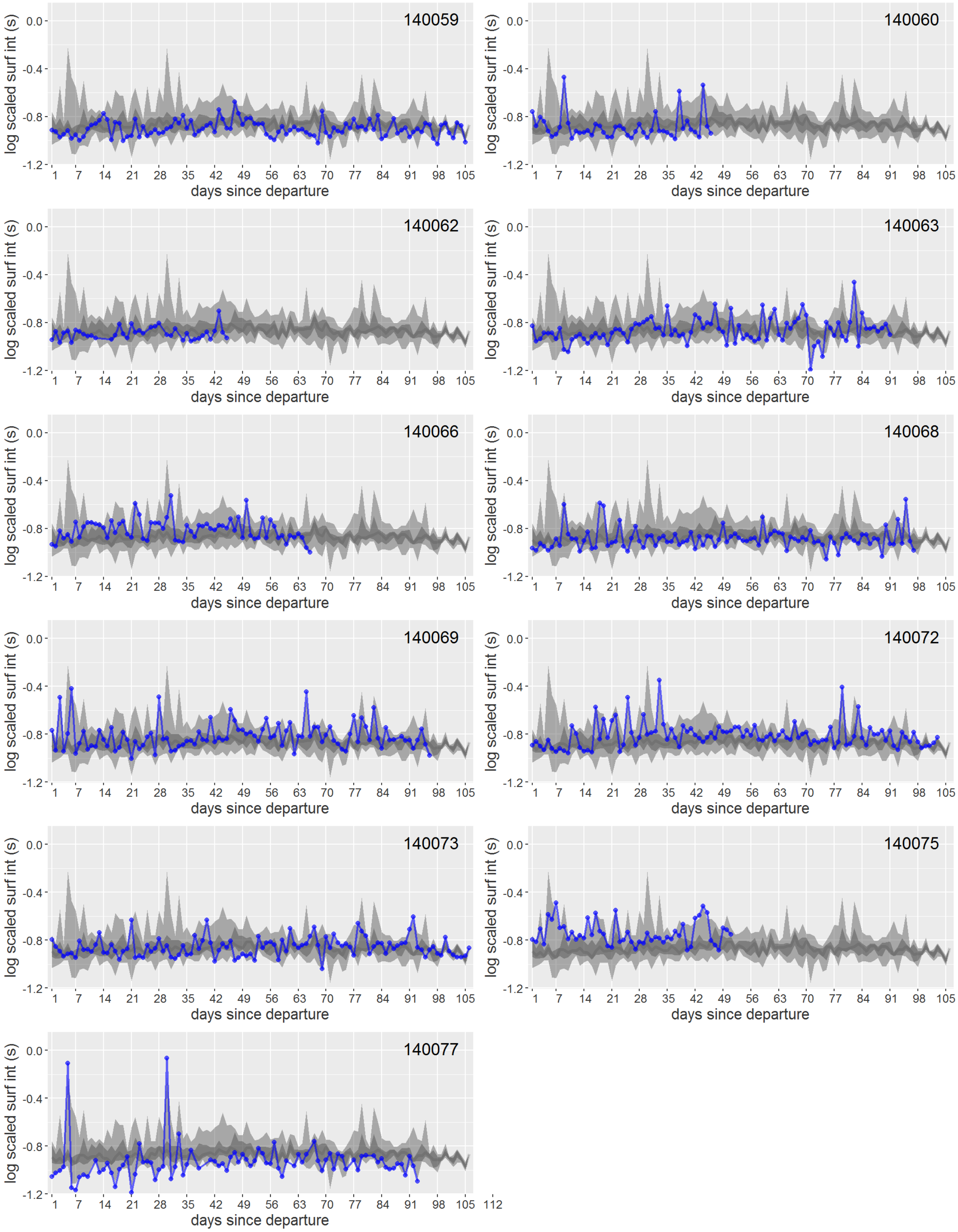

Figure S26. Time series plots of daily mean surface intervals (logged) for each individual that survived (for plots of individuals that died see Figure S25). Large light grey bands represent the 2.5% to 97.5% quantiles of the survival datasets. Nested within this in darker grey are the 25 to 75% quantiles. The daily median of the survival data is represented by a dark grey line. The blue line indicates the daily values of the individual identified in the top right-hand corner.

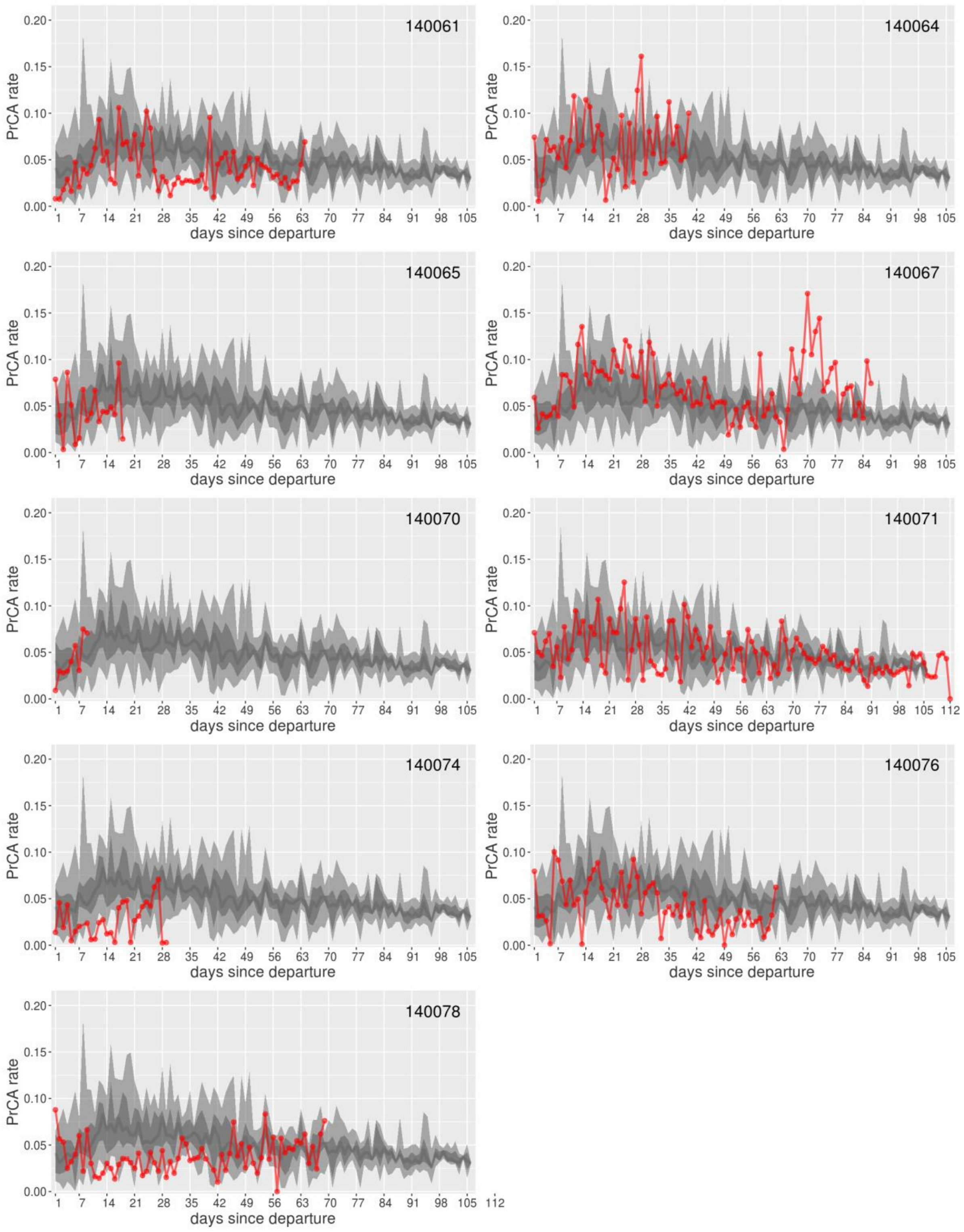

Figure S27. Time series plots of mean daily prey catch attempt (PrCA) rates for each individual that died (for plots of individuals that survived see Figure S28). Large light grey bands represent the 2.5% to 97.5% quantiles of the survival datasets. Nested within this in darker grey are the 25 to 75% quantiles. The daily median of the survival data is represented by a dark grey line. The red line indicates the daily values of the individual identified in the top right-hand corner.

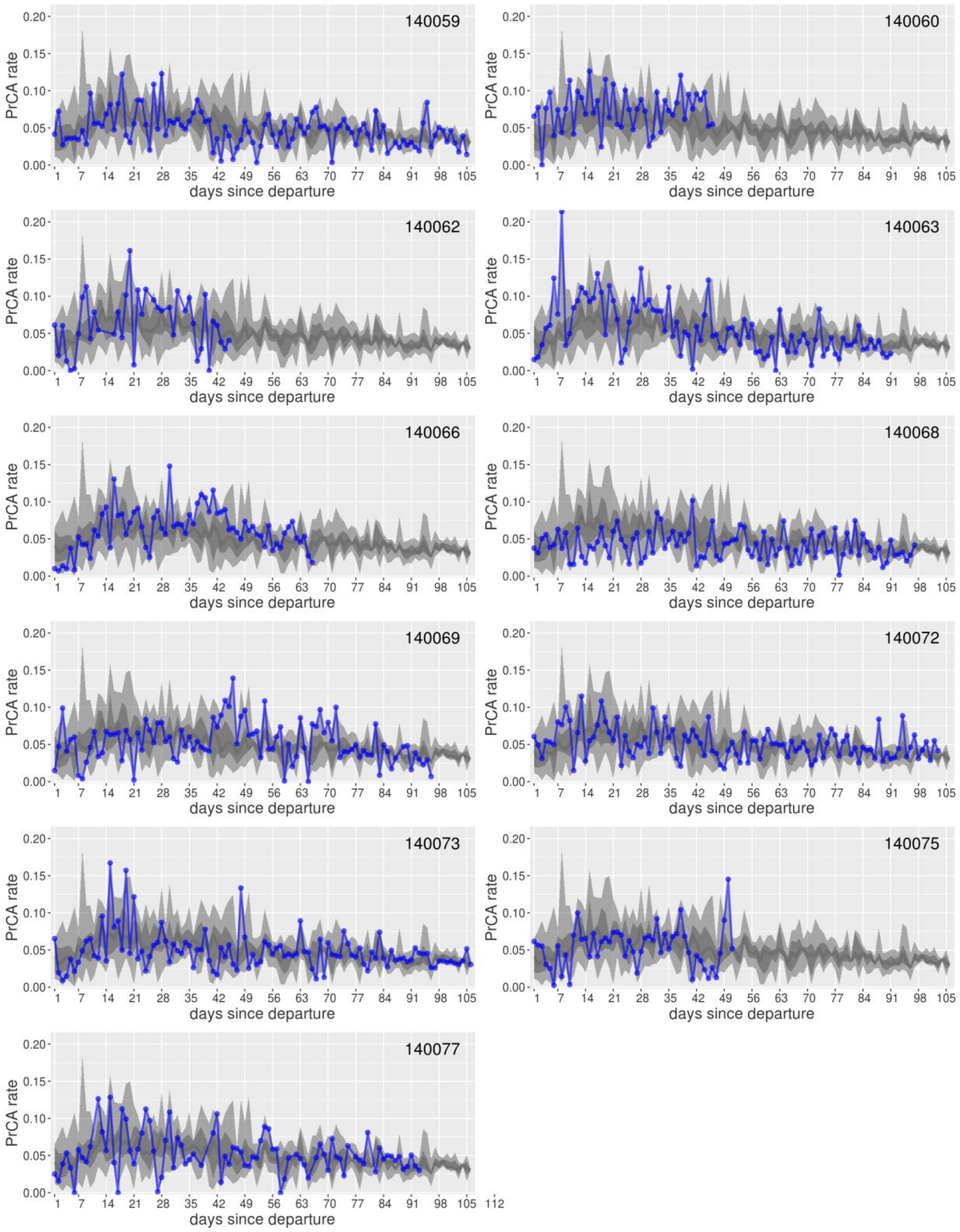

Figure S28. Time series plots of mean daily prey catch attempt (PrCA) rates for each individual that survived (for plots of individuals that died see Figure S27). Large light grey bands represent the 2.5% to 97.5% quantiles of the survival datasets. Nested within this in darker grey are the 25 to 75% quantiles. The daily median of the survival data is represented by a dark grey line. The blue line indicates the daily values of the individual identified in the top right-hand corner.

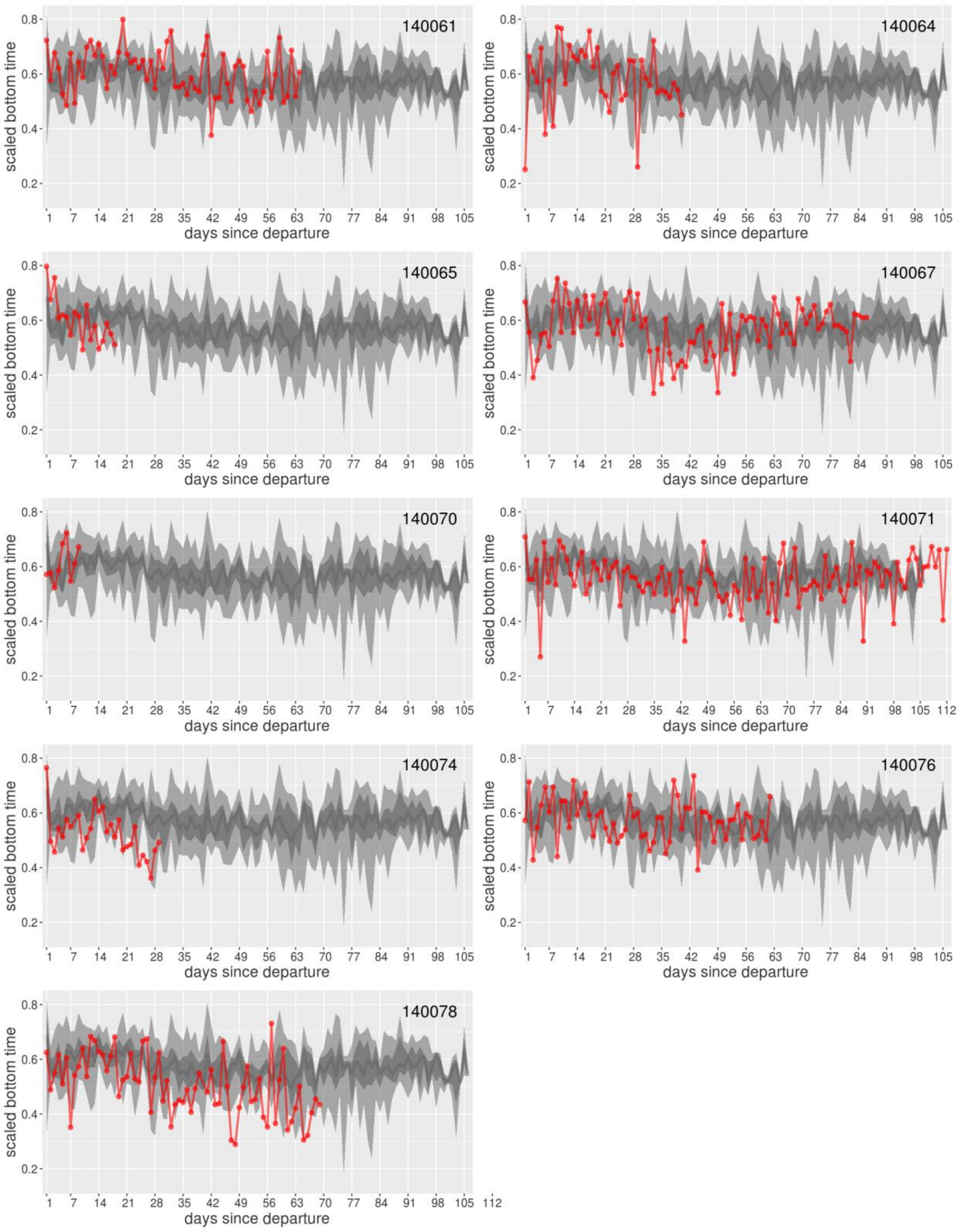

Figure S29. Time series plots of daily mean scaled bottom times (by dive duration) for each individual that died (for plots of individuals that survived see Figure S30). Large light grey bands represent the 2.5% to 97.5% quantiles of the survival datasets. Nested within this in darker grey are the 25 to 75% quantiles. The daily median of the survival data is represented by a dark grey line. The red line indicates the daily values of the individual identified in the top right-hand corner.

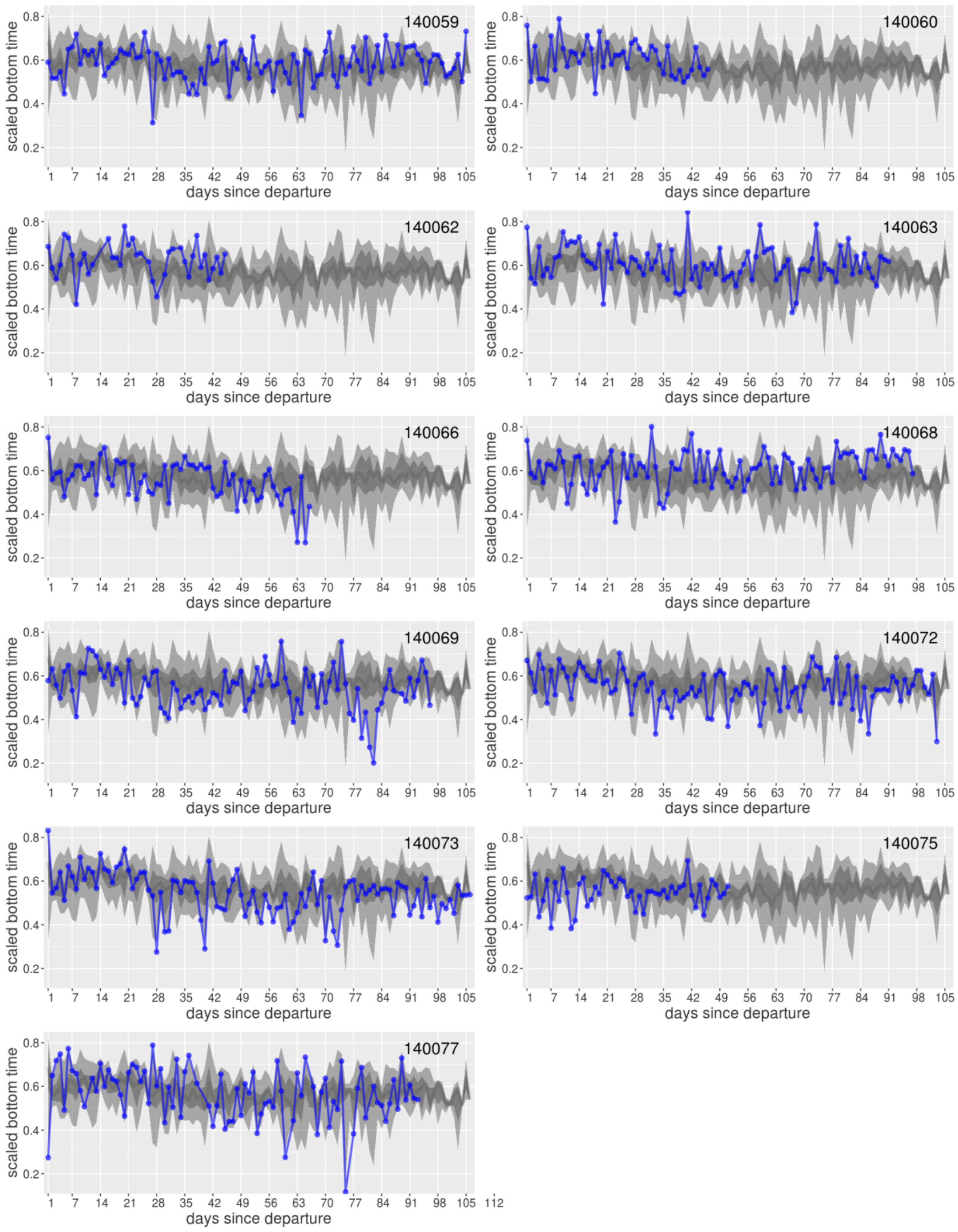

Figure S30. Time series plots of daily mean scaled bottom times (by dive duration) for each individual that survived (for plots of individuals that died see Figure S29). Large light grey bands represent the 2.5% to 97.5% quantiles of the survival datasets. Nested within this in darker grey are the 25 to 75% quantiles. The daily median of the survival data is represented by a dark grey line. The blue line indicates the daily values of the individual identified in the top right-hand corner.

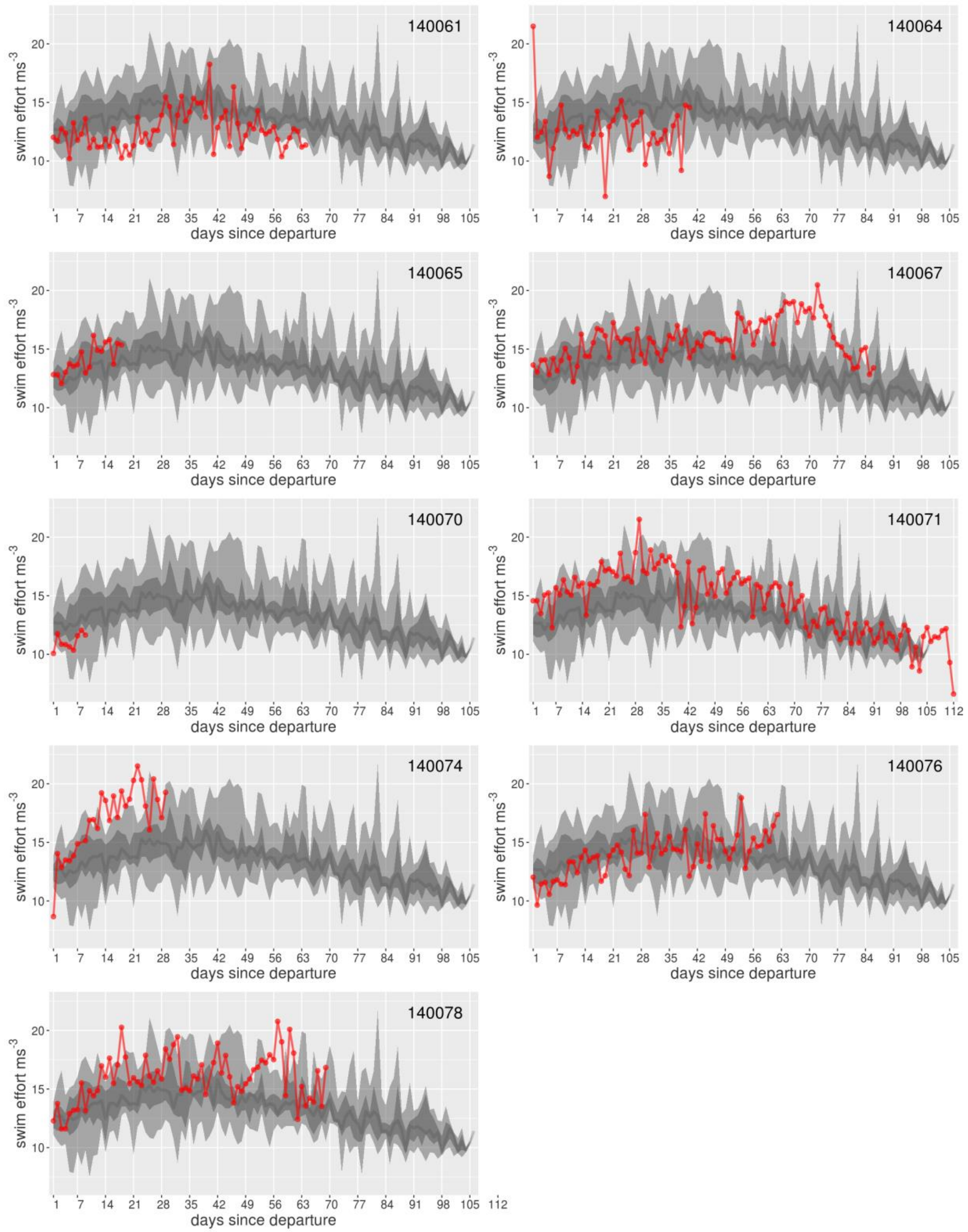

Figure S31. Time series plots of daily mean total ascent and descent swimming efforts for each individual that died (for plots of individuals that survived see Figure S32). Large light grey bands represent the 2.5% to 97.5% quantiles of the survival datasets. Nested within this in darker grey are the 25 to 75% quantiles. The daily median of the survival data is represented by a dark grey line. The red line indicates the daily values of the individual identified in the top right-hand corner.

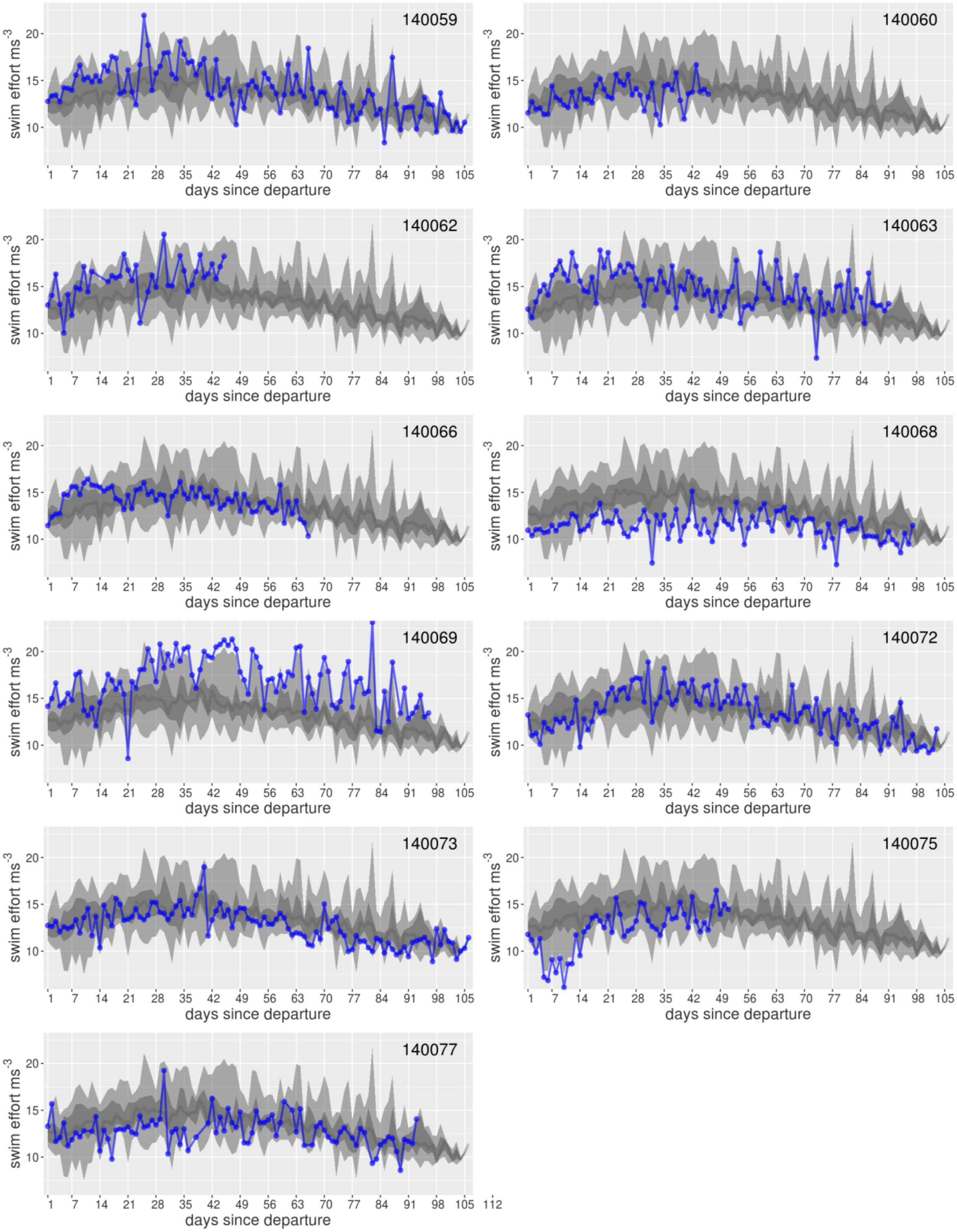

Figure S32. Time series plots of daily mean total ascent and descent swimming efforts for each individual that survived (for plots of individuals that died see Figure S31). Large light grey bands represent the 2.5% to 97.5% quantiles of the survival datasets. Nested within this in darker grey are the 25 to 75% quantiles. The daily median of the survival data is represented by a dark grey line. The blue line indicates the daily values of the individual identified in the top right-hand corner.

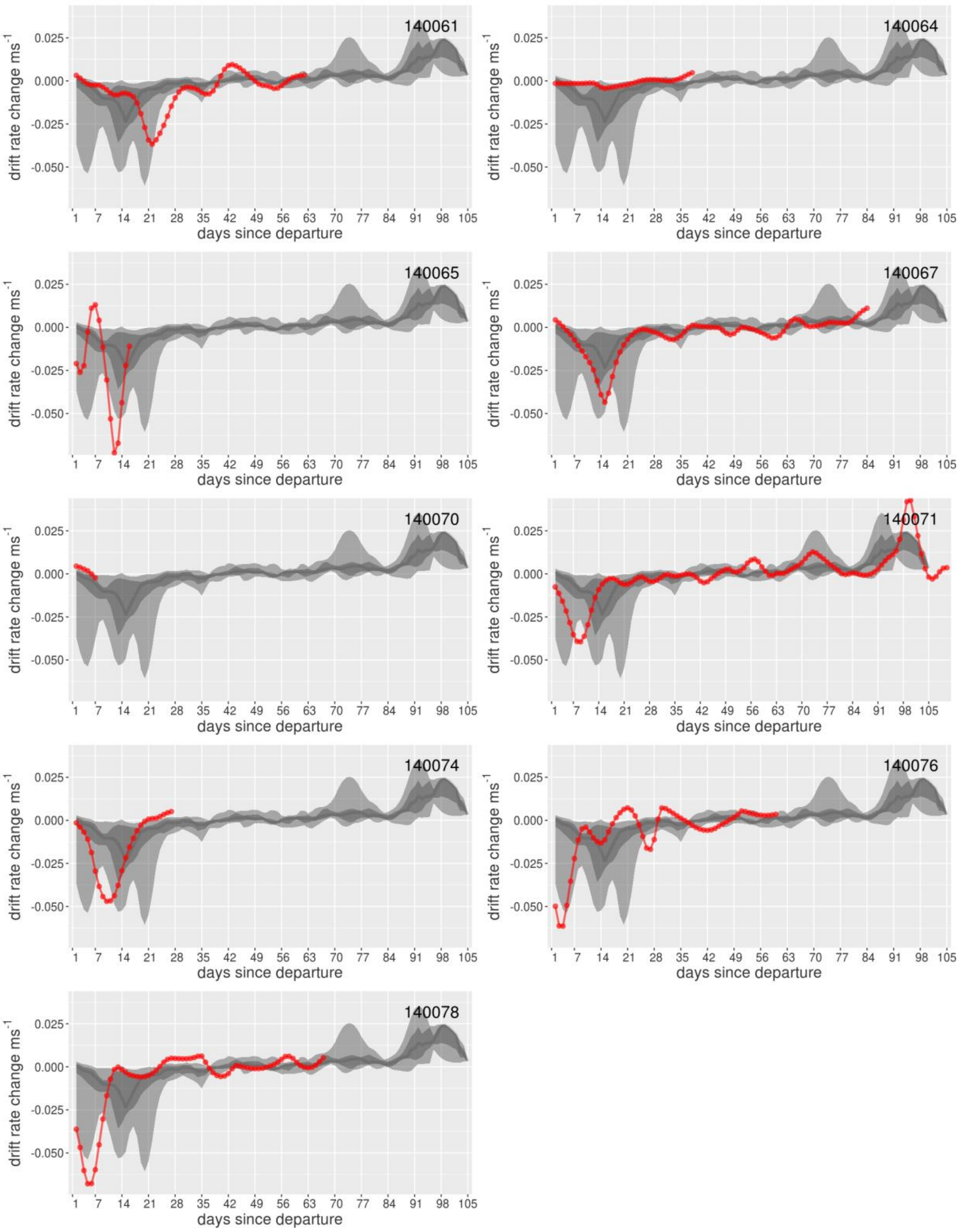

Figure S33. Time series plots of daily changes in drift rate by each individual that died (for plots of individuals that survived see Figure S34). Large light grey bands represent the 2.5% to 97.5% quantiles of the survival datasets. Nested within this in darker grey are the 25 to 75% quantiles. The daily median of the survival data is represented by a dark grey line. The red line indicates the daily values of the individual identified in the top right-hand corner.

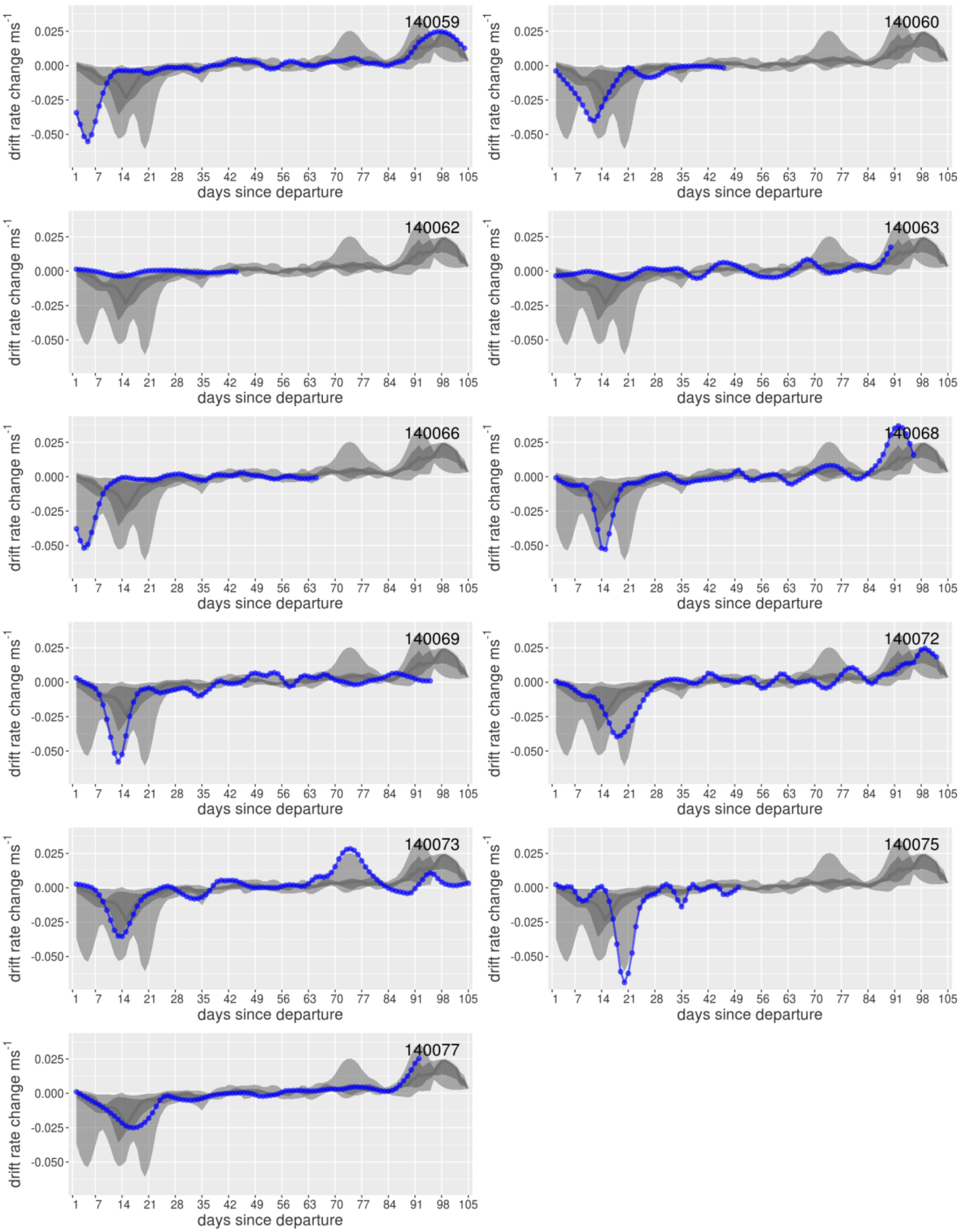

Figure S34. Time series plots of daily changes in drift rate by each individual that survived (for plots of individuals that died see Figure S33). Large light grey bands represent the 2.5% to 97.5% quantiles of the survival datasets. Nested within this in darker grey are the 25 to 75% quantiles. The daily median of the survival data is represented by a dark grey line. The blue line indicates the daily values of the individual identified in the top right-hand corner.

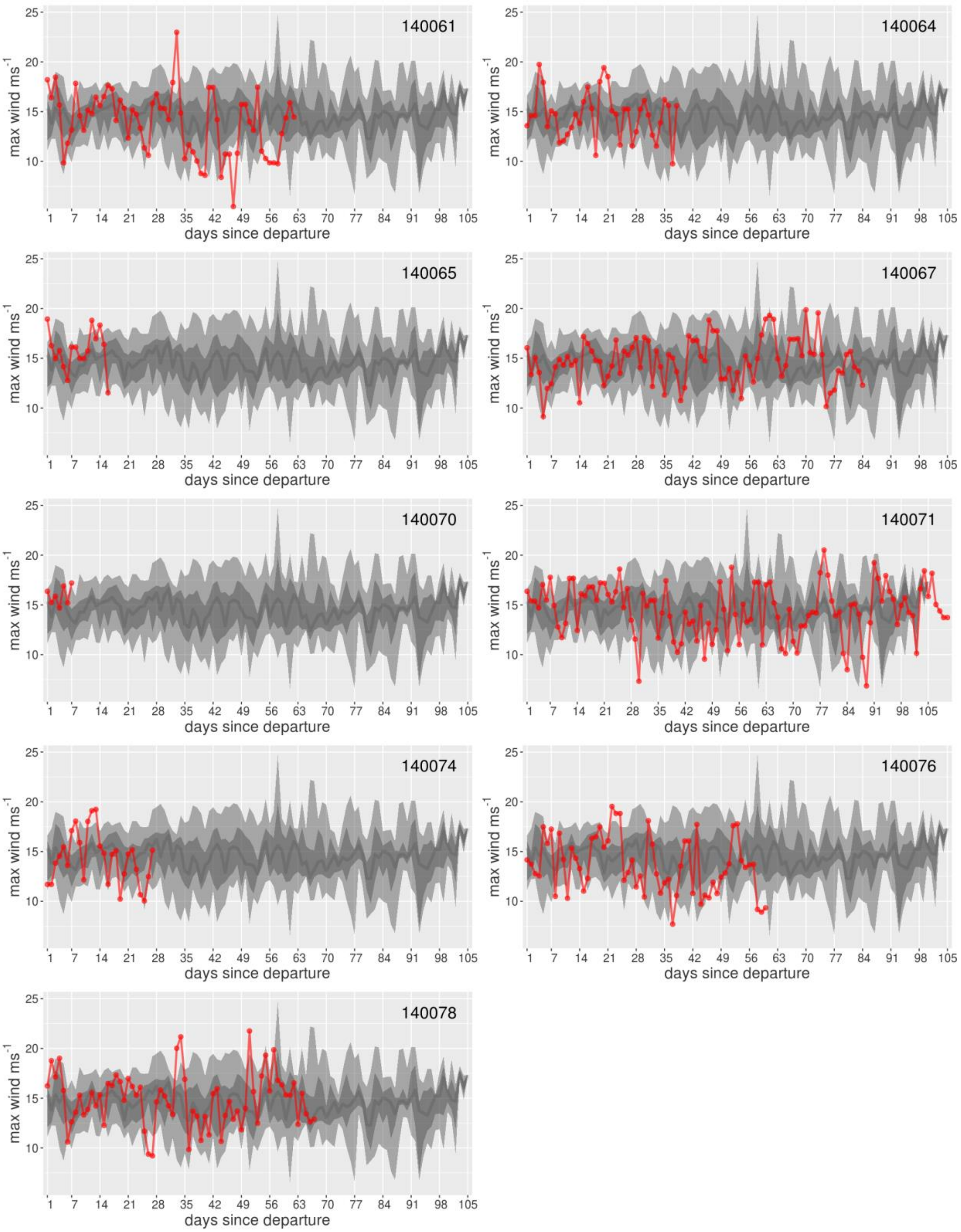

Figure S35. Time series plots of maximum daily wind conditions experienced by each individual that died (for plots of individuals that survived see Figure S36). Large light grey bands represent the 2.5% to 97.5% quantiles of the survival datasets. Nested within this in darker grey are the 25 to 75% quantiles. The daily median of the survival data is represented by a dark grey line. The red line indicates the daily values of the individual identified in the top right-hand corner.

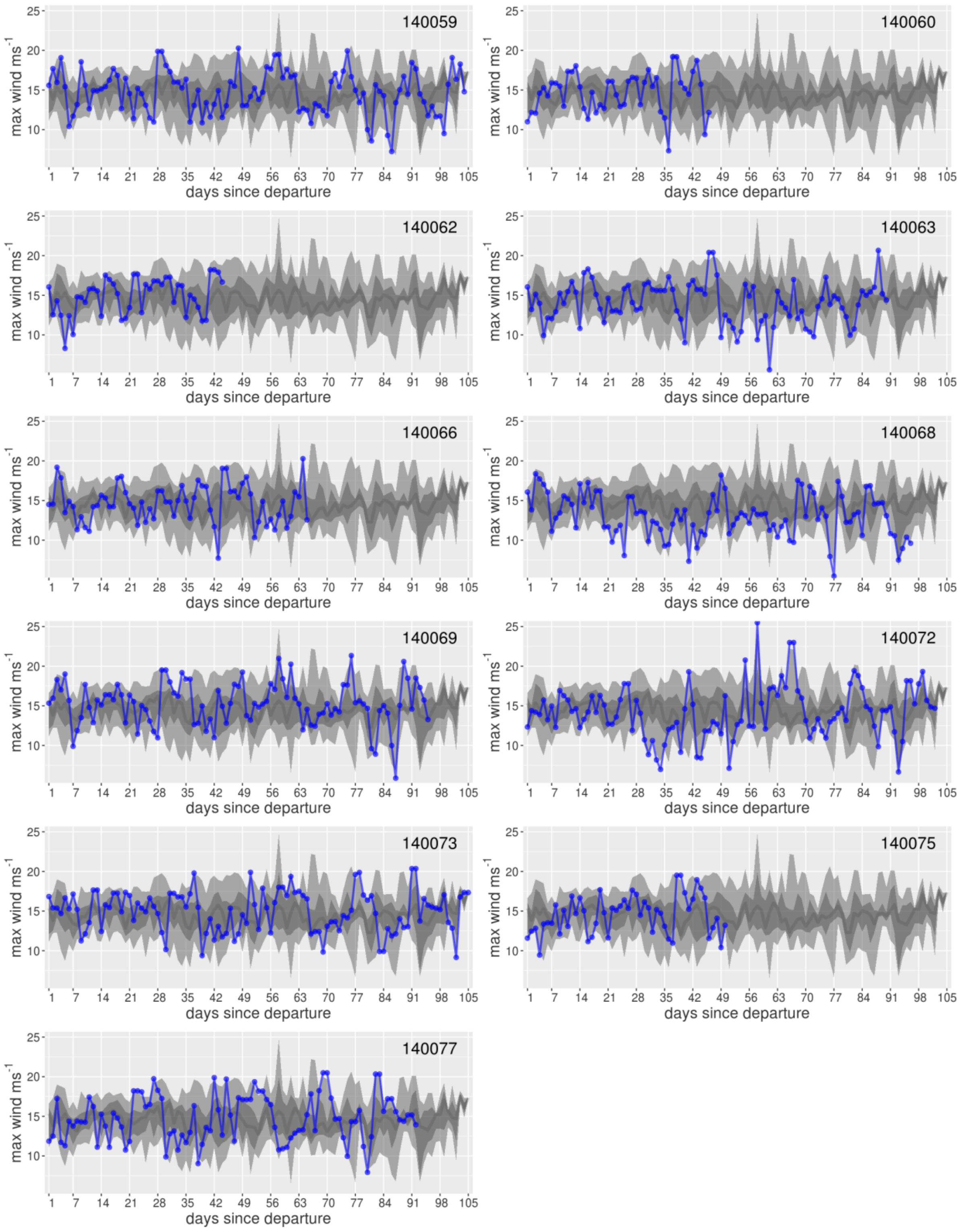

Figure S36. Time series plots of maximum daily wind conditions experienced by each individual that survived (for plots of individuals that died see Figure S35). Large light grey bands represent the 2.5% to 97.5% quantiles of the survival datasets. Nested within this in darker grey are the 25 to 75% quantiles. The daily median of the survival data is represented by a dark grey line. The blue line indicates the daily values of the individual identified in the top right-hand corner.

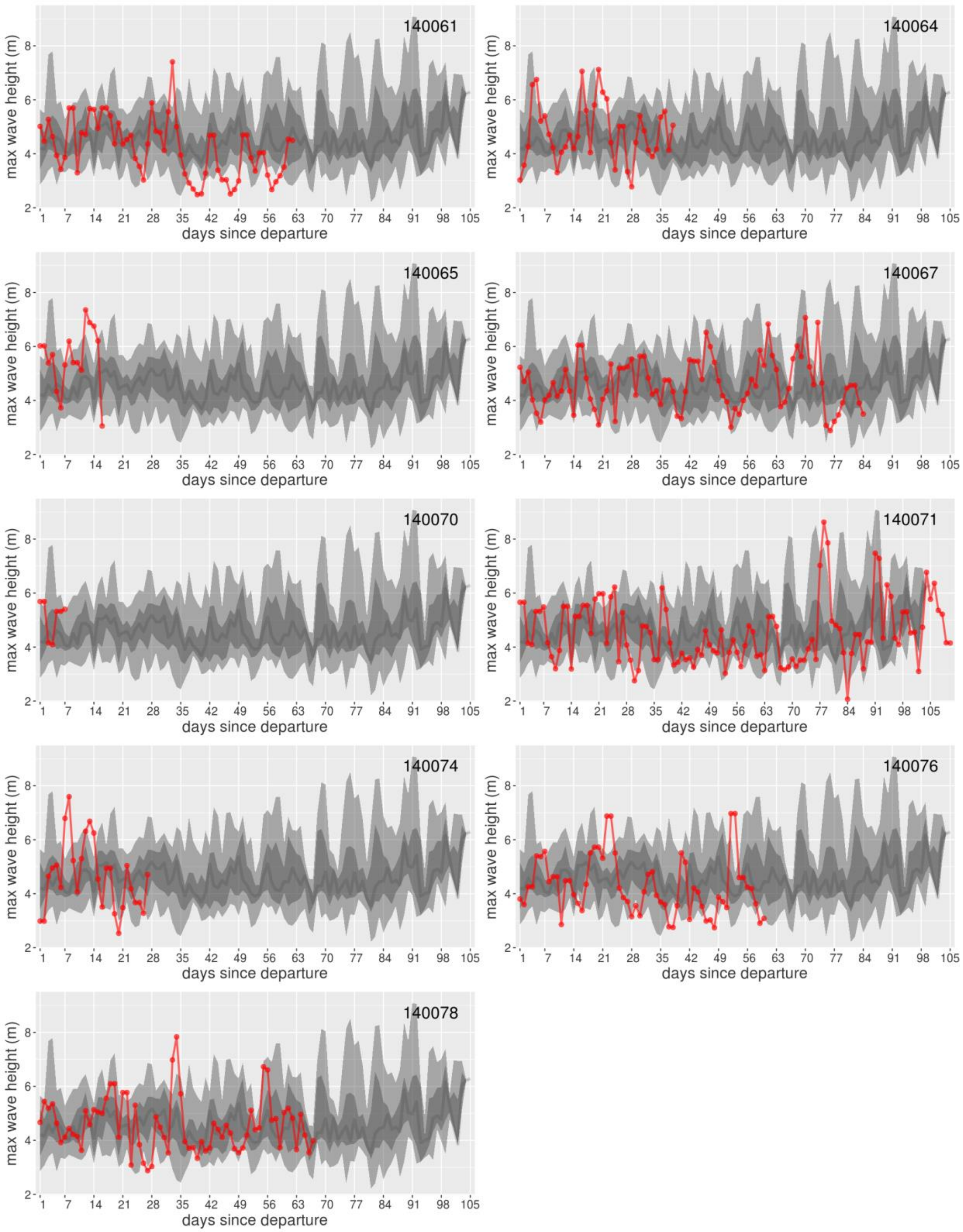

Figure S37. Time series plots of maximum daily wave heights experienced by each individual that died (for plots of individuals that survived see Figure S38). Large light grey bands represent the 2.5% to 97.5% quantiles of the survival datasets. Nested within this in darker grey are the 25 to 75% quantiles. The daily median of the survival data is represented by a dark grey line. The red line indicates the daily values of the individual identified in the top right-hand corner.

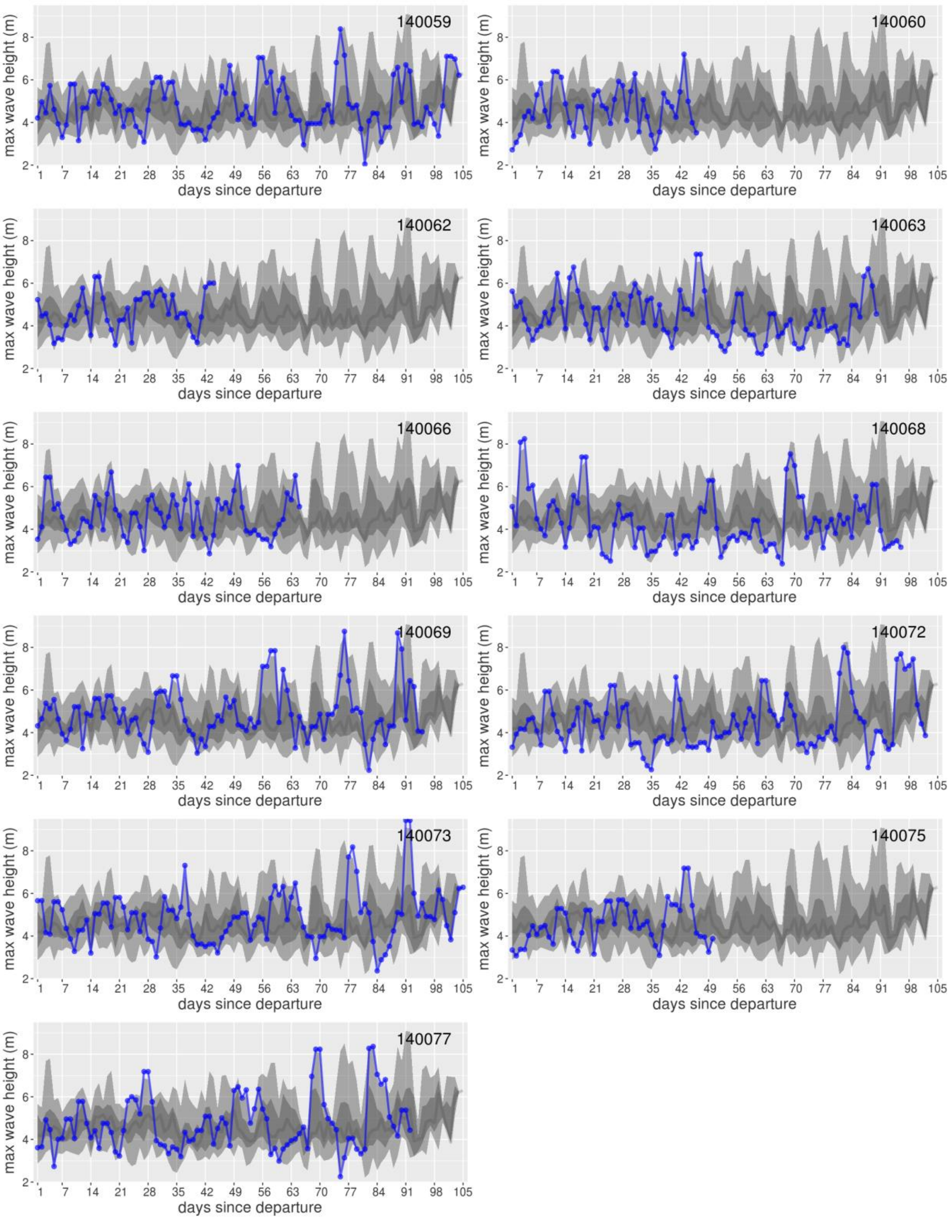

Figure S38. Time series plots of maximum daily wave heights experienced by each individual that survived (for plots of individuals that died see Figure S37). Large light grey bands represent the 2.5% to 97.5% quantiles of the survival datasets. Nested within this in darker grey are the 25 to 75% quantiles. The daily median of the survival data is represented by a dark grey line. The blue line indicates the daily values of the individual identified in the top right-hand corner.

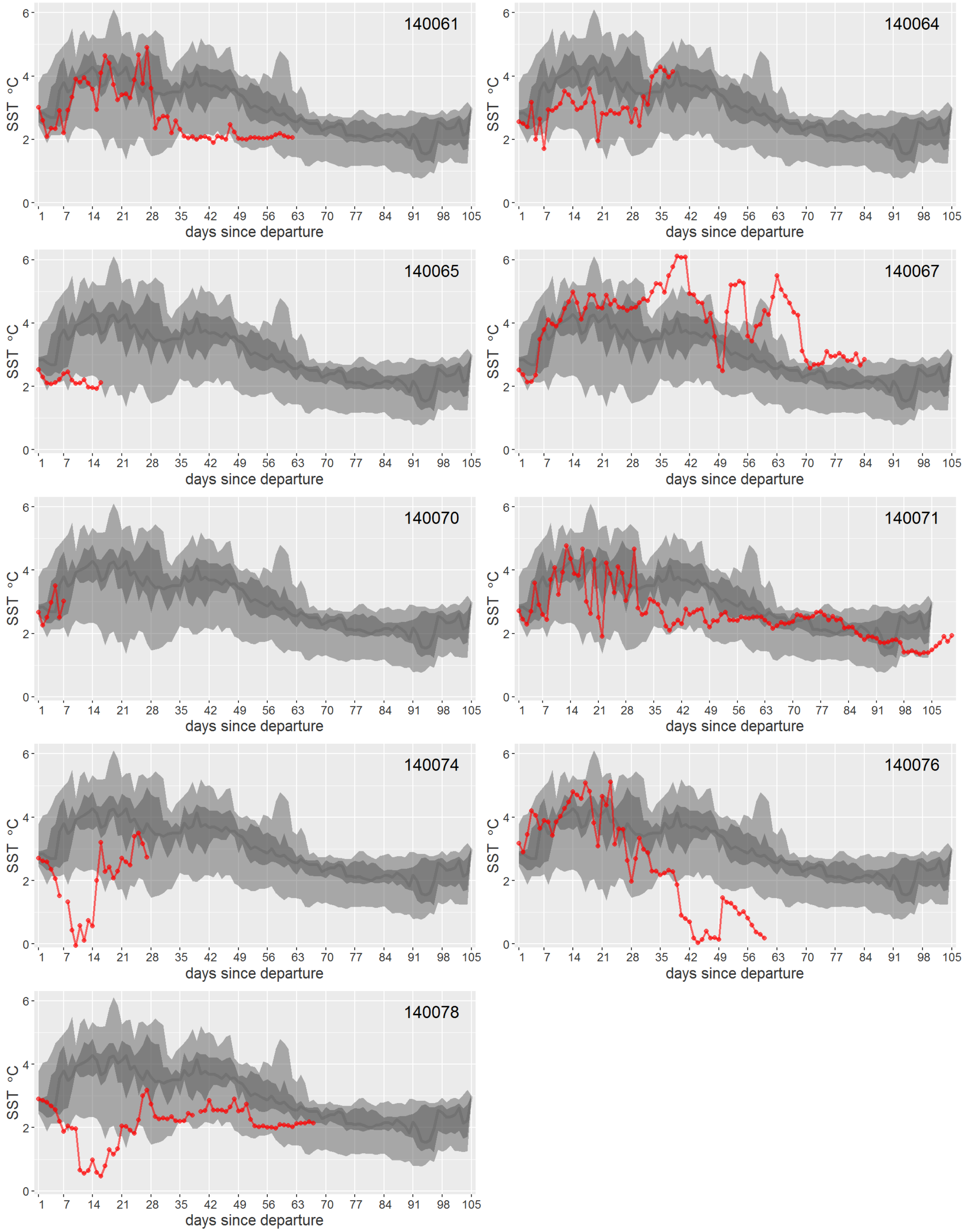

Figure S39. Time series plots of daily mean sea surface temperature (SST) experienced by each individual that died (for plots of individuals that survived see Figure S40). Large light grey bands represent the 2.5% to 97.5% quantiles of the survival datasets. Nested within this in darker grey are the 25 to 75% quantiles. The daily median of the survival data is represented by a dark grey line. The red line indicates the daily values of the individual identified in the top right-hand corner.

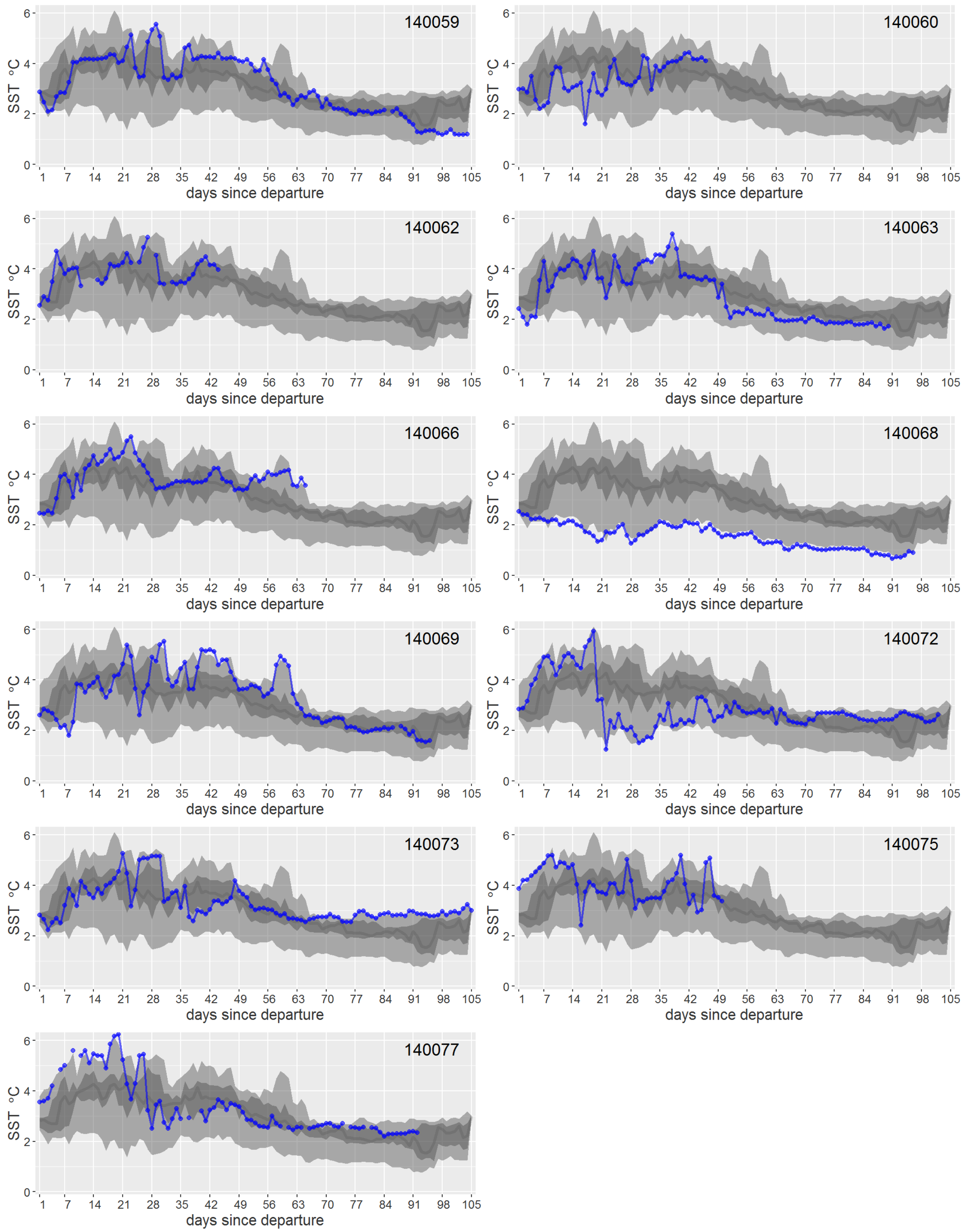

Figure S40. Time series plots of daily mean sea surface temperature (SST) experienced by each individual that survived (for plots of individuals that died see Figure S39). Large light grey bands represent the 2.5% to 97.5% quantiles of the survival datasets. Nested within this in darker grey are the 25 to 75% quantiles. The daily median of the survival data is represented by a dark grey line. The blue line indicates the daily values of the individual identified in the top right-hand corner.

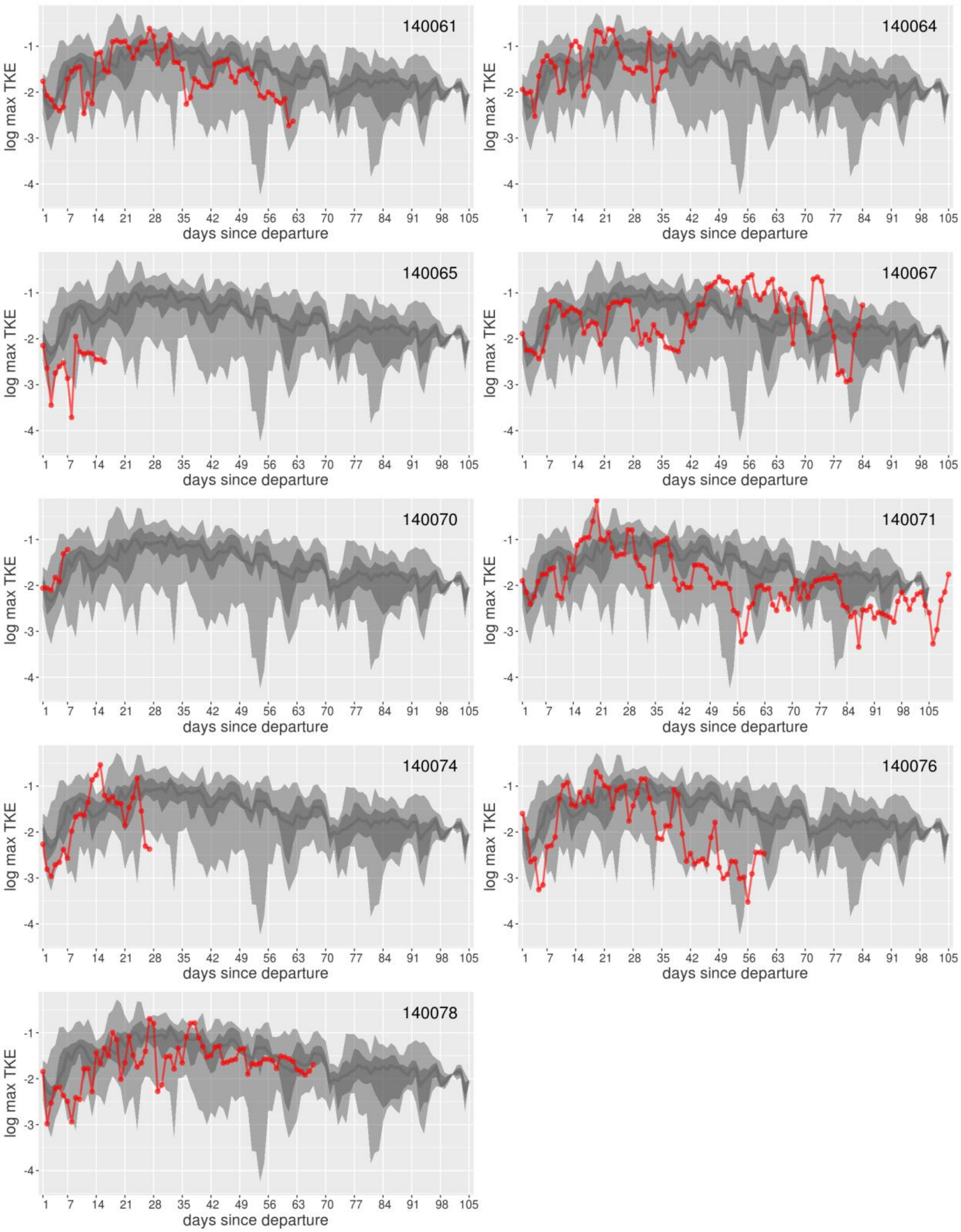

Figure S41. Time series plots of daily maximum total (eddy) kinetic energy (TKE; logged) experienced by each individual that died (for plots of individuals that survived see Figure S42). Large light grey bands represent the 2.5% to 97.5% quantiles of the survival datasets. Nested within this in darker grey are the 25 to 75% quantiles. The daily median of the survival data is represented by a dark grey line. The red line indicates the daily values of the individual identified in the top right-hand corner.

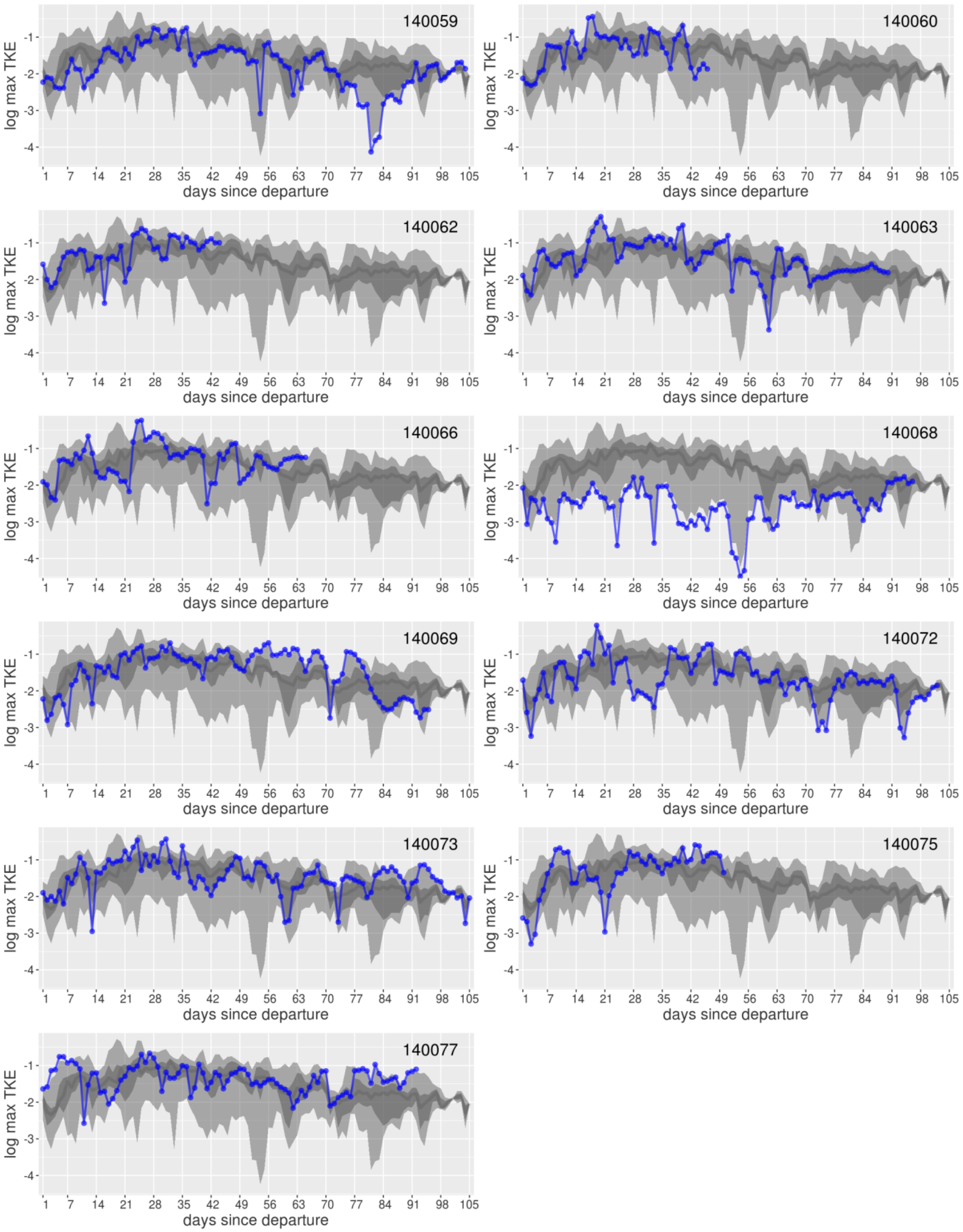

Figure S42. Time series plots of daily maximum total (eddy) kinetic energy (TKE; logged) experienced by each individual that survived (for plots of individuals that died see Figure S41). Large light grey bands represent the 2.5% to 97.5% quantiles of the survival datasets. Nested within this in darker grey are the 25 to 75% quantiles. The daily median of the survival data is represented by a dark grey line. The blue line indicates the daily values of the individual identified in the top right-hand corner.

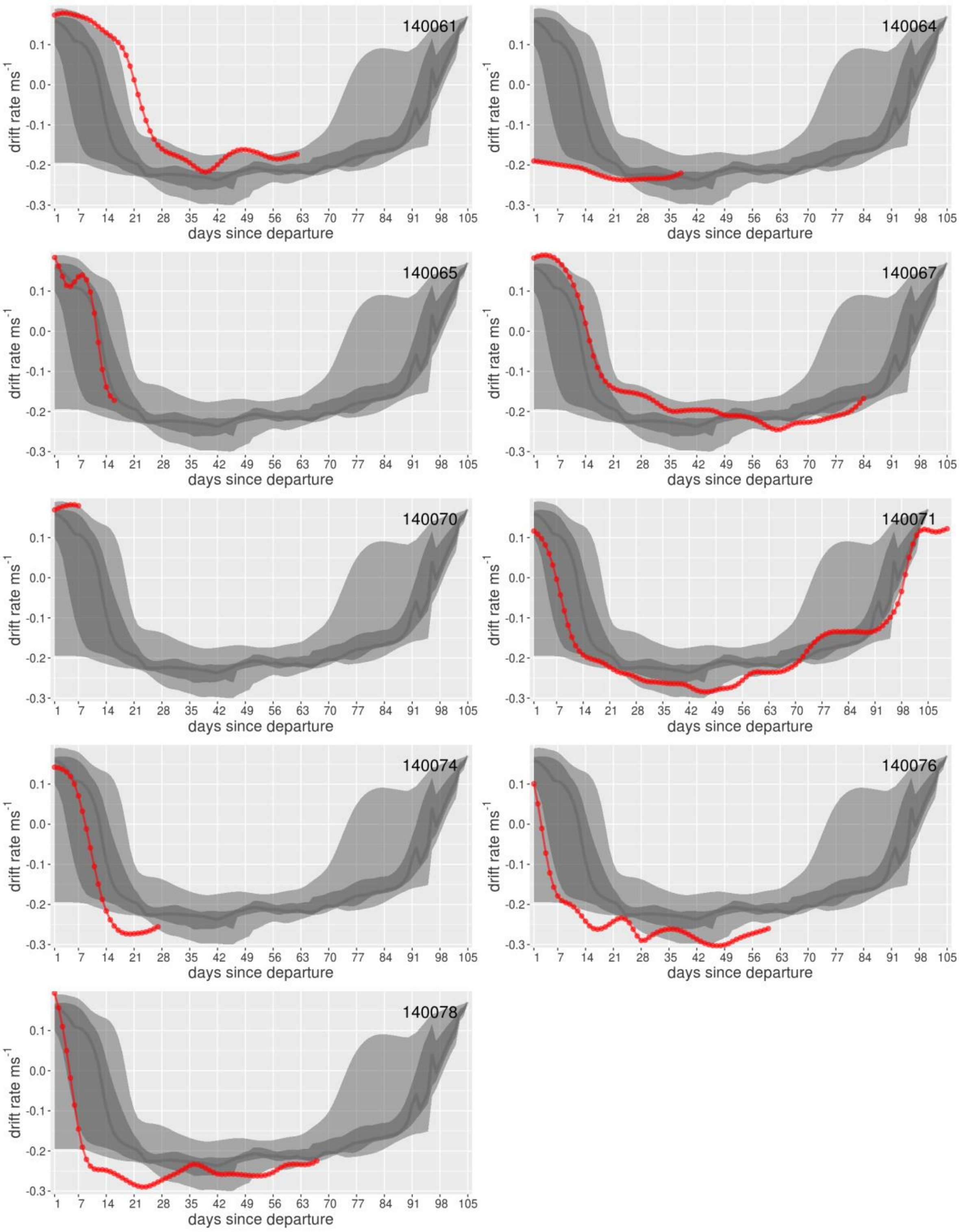

Figure S43. Time series plots of daily drift rates for each individual that died (for plots of individuals that survived see Figure S44). Large light grey bands represent the 2.5% to 97.5% quantiles of the survival datasets. Nested within this in darker grey are the 25 to 75% quantiles. The daily median of the survival data is represented by a dark grey line. The red line indicates the daily values of the individual identified in the top right-hand corner.

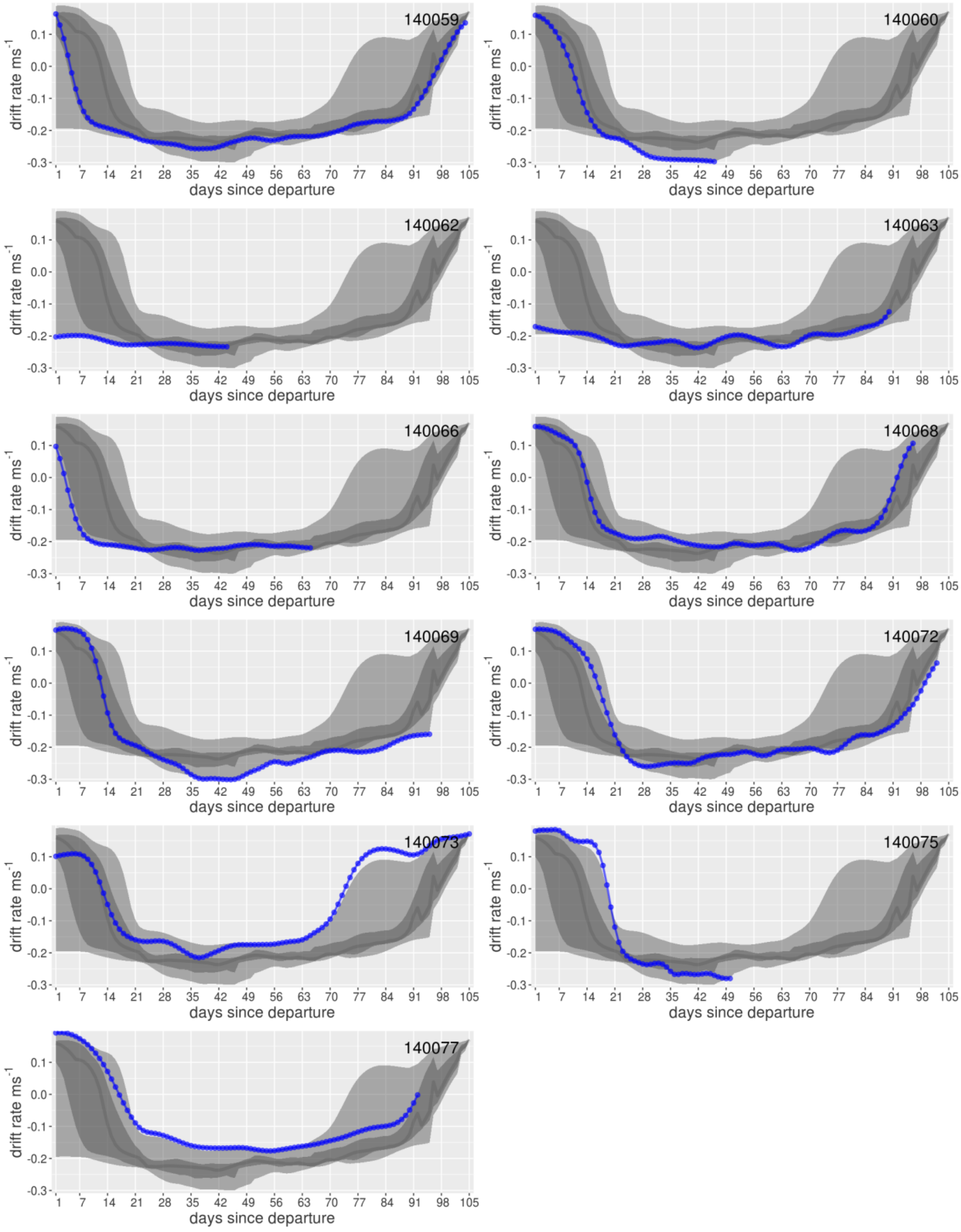

Figure S44. Time series plots of daily drift rates for each individual that survived (for plots of individuals that died see Figure S43). Large light grey bands represent the 2.5% to 97.5% quantiles of the survival datasets. Nested within this in darker grey are the 25 to 75% quantiles. The daily median of the survival data is represented by a dark grey line. The blue line indicates the daily values of the individual identified in the top right-hand corner.

S.9 Comparisons between survivors and non-survivors of maximum total (eddy) kinetic energy (TKE) values of waters visited

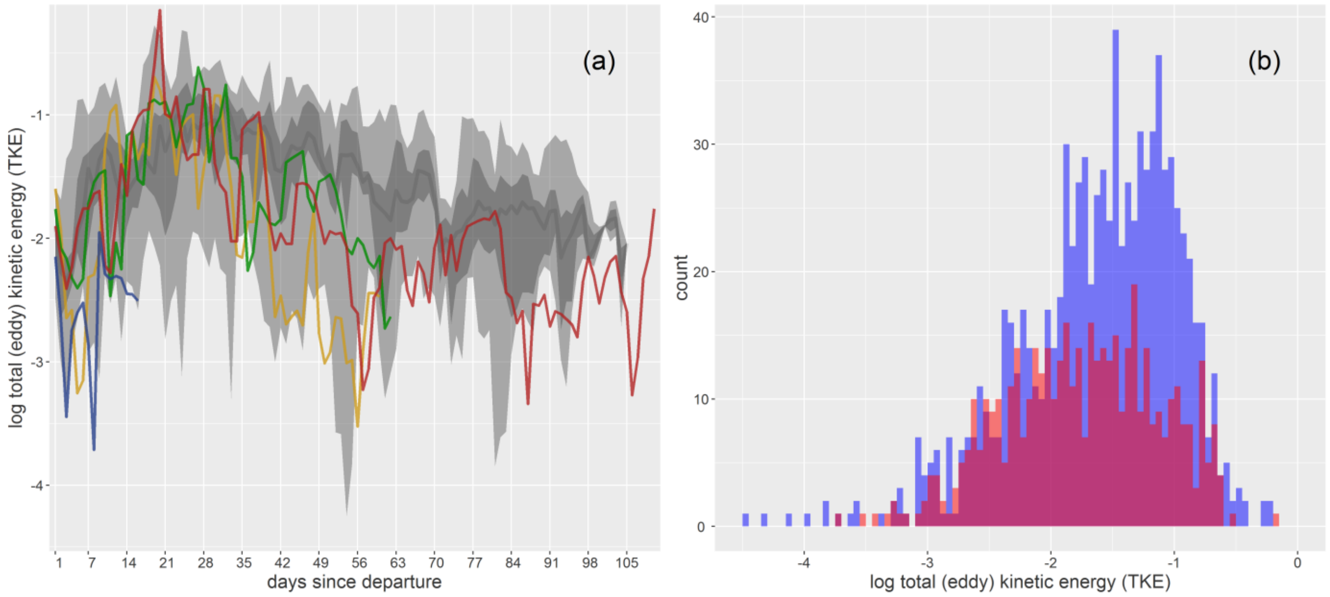

Figure S45. Logged maximum total (eddy) kinetic energy (TKE) values of the waters visited by the group of individuals that survived versus those that died. On the left (a), is a time series plot for four individuals (140061, 140065, 140071 and 140076; coloured lines) that appeared to visit waters of reduced maximum logged TKE values. Here, the large light grey bands represent the 2.5% to 97.5% quantiles of survival datasets, whilst the darker grey band nested within this is the 25 to 75% quantiles. The daily median of the survival data is represented by a dark grey line. Individual plots for all tracked pups are in supplementary information S.8. On the right (b), is a histogram of the maximum logged TKE values measured by surviving pups (blue) and dying pups (red), during the first portion (up until distal point or death) of a trip.

S.10 Variable selection within the horseshoe survival analysis

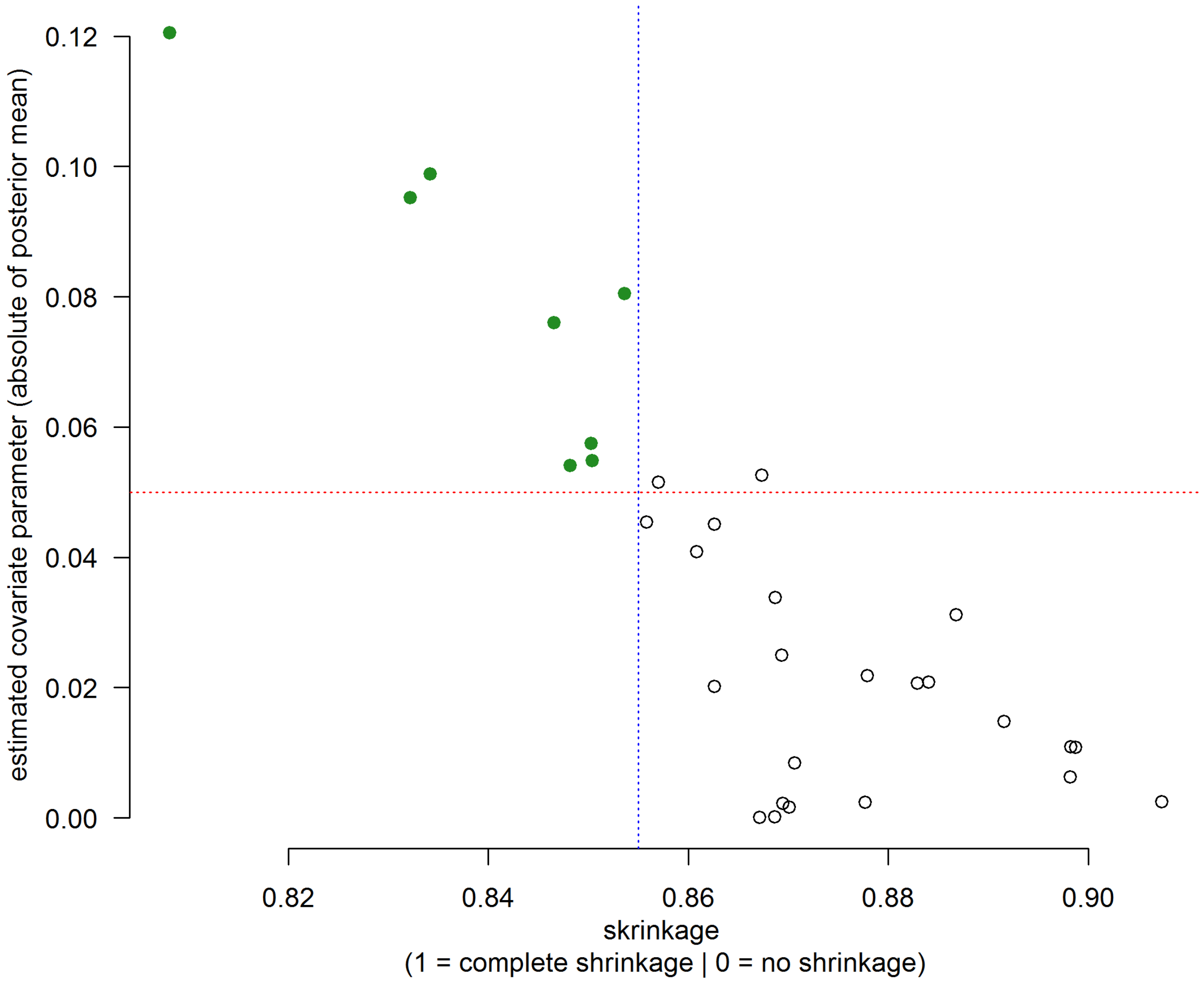

Figure S46. Absolute estimated covariate parameters plotted against shrinkage values from the horseshoe analysis. Covariates with an absolute parameter estimate of at least 0.05 (dashed horizontal red line) and a shrinkage value less than 0.855 (dashed vertical blue line) were considered to impact juvenile survival, whilst those with parameter estimates below 0.05 or shrinkage values above 0.855 were not.

S.11 Departure weights and conditions

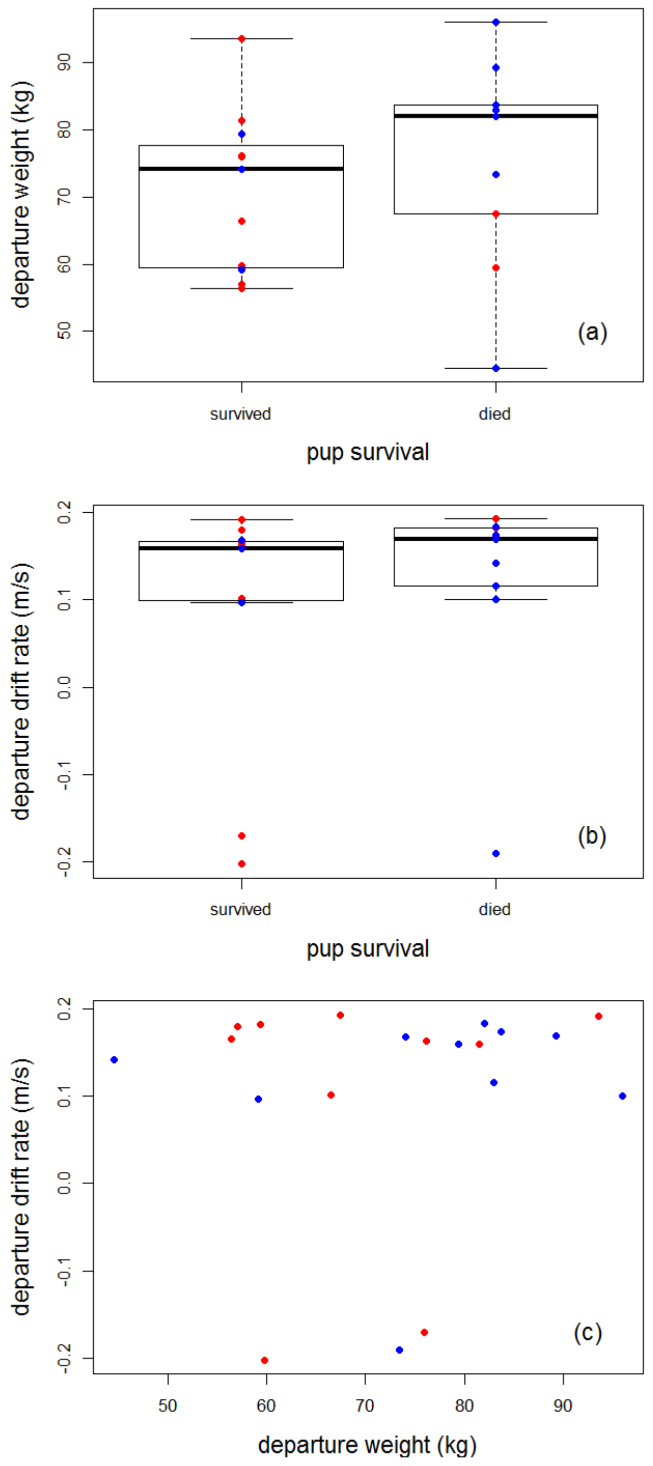

Figure S47. From top to bottom: (a) boxplot of weights at departure for survivors (left) and non-survivors (right), (b) boxplot of drift rates at departure for survivors (left) and non-survivors (right) and (c) departure drift rates in relation to departure weight. In all plots, red markers indicate females and blue, males.

S.12 Prey catch attempt (PrCA) rates and maximum total (eddy) kinetic energy (TKE)

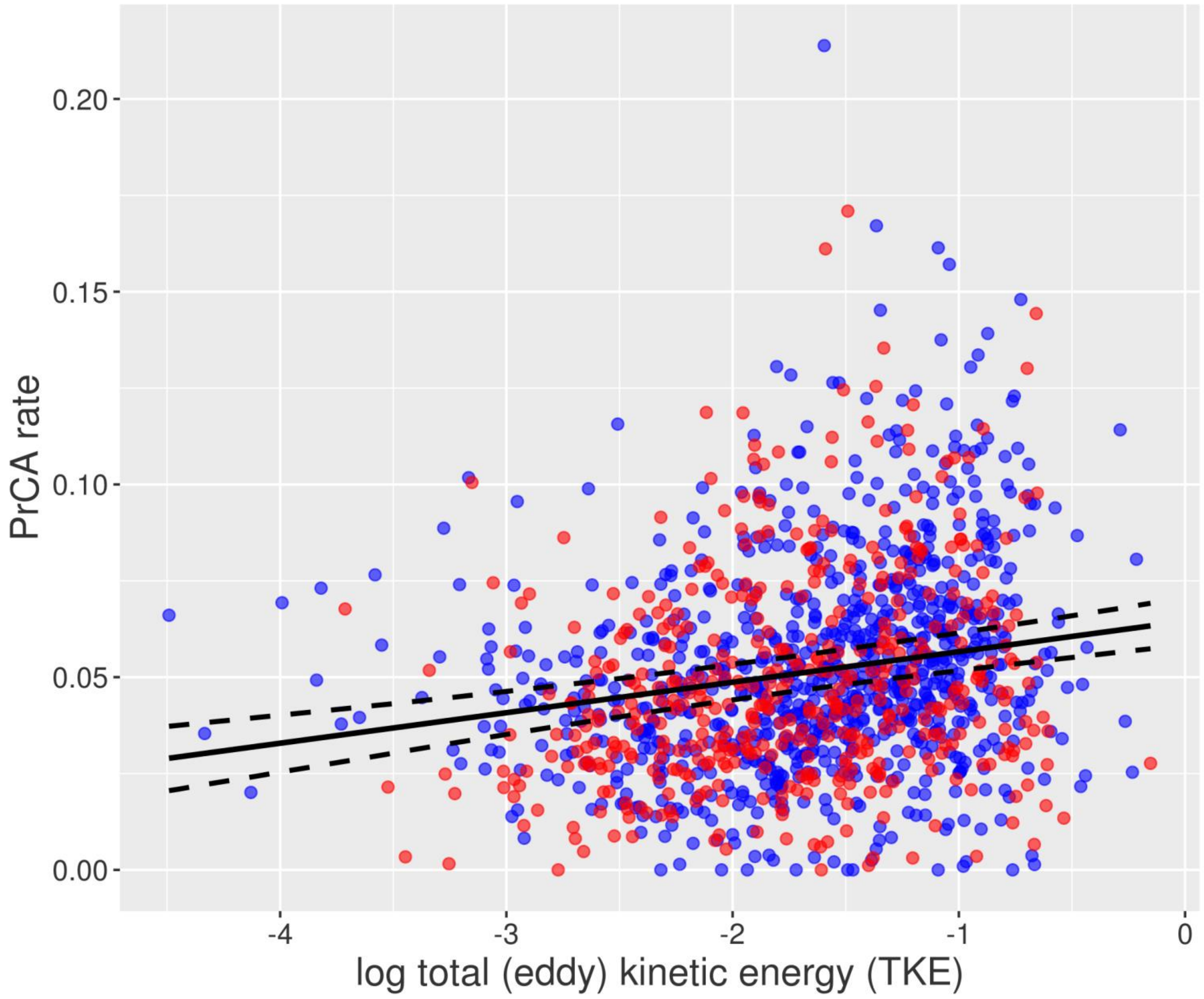

Figure S48. Mean daily PrCA rates in relation to logged daily maximum total (eddy) kinetic energy (TKE). Observations from surviving and non-surviving individuals are indicated in blue and red respectively. In black is the line of best fit from linear mixed effects models (with a random intercept of individual ID; nlme package in R - Pinheiro & Bates 2014). 95% confidence intervals are indicated by the dashed lines.

S.13 Spatial distributions of juvenile southern elephant seals in relation to sea surface temperatures (SST) and surface currents for four days during the austral summer

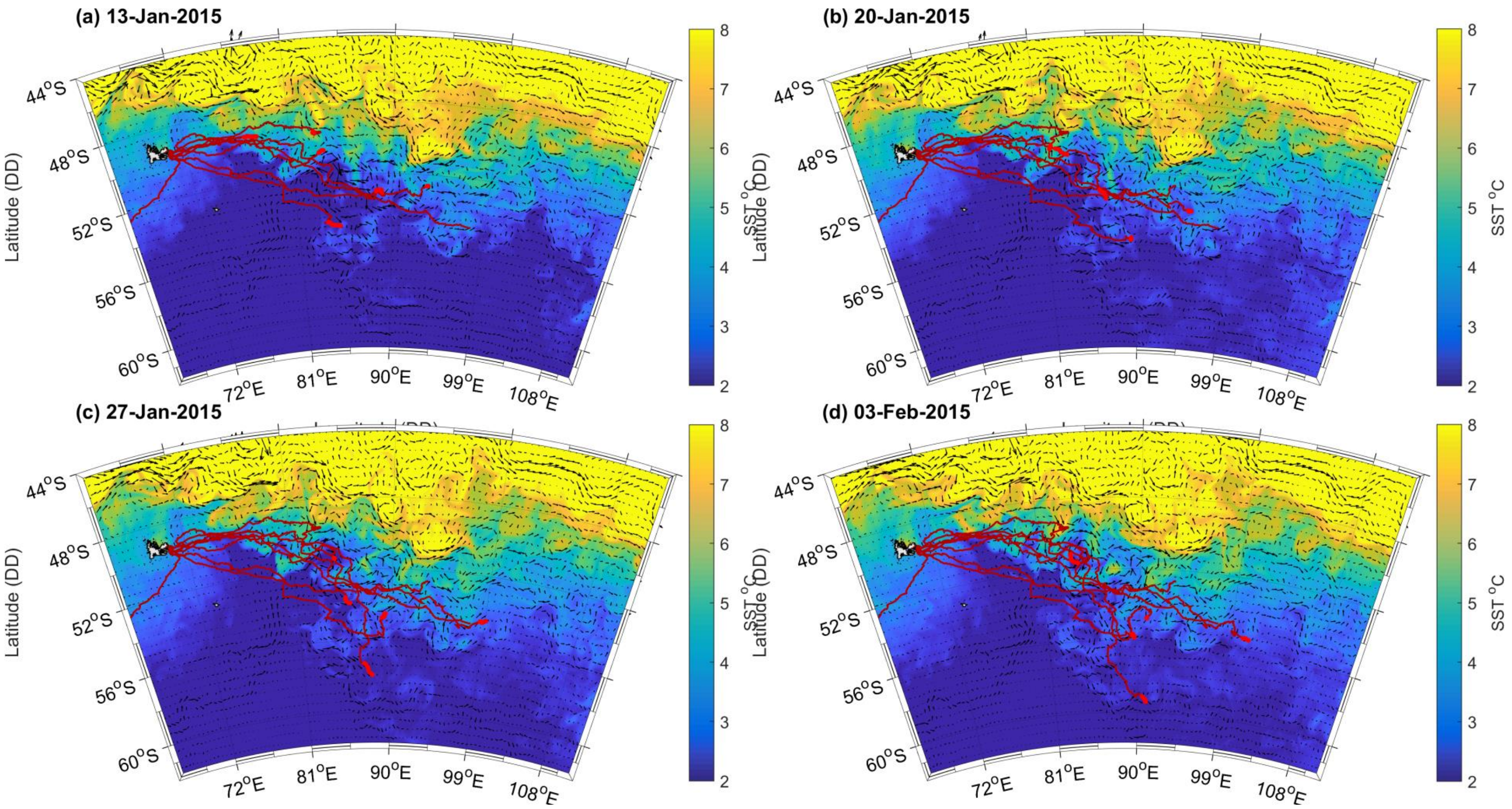

Figure S49. Trajectories of non-surviving pups on four days of the austral summer of 2015: (a) 13th January, (b) 20th January, (c) 27th January and (d) 3rd February. Bright red markers are locations concurrent to the day, and dark red lines, previous trajectories since leaving Kerguelen Islands. Black arrows are surface current directions with strength indicated by the size of the arrow (bolder, larger arrows are stronger currents). These were taken from the delayed time all-sat-merged Global Ocean Gridded Absolute Geostrophic Velocities Anomalies L4 product, obtained via the Archiving Validation and Interpretation of Satellite and Oceanographic data portal on a daily basis at a spatial resolution of 0.25° (AVISO; www.aviso.altimetry.fr). Background colours are sea surface temperatures (SST). These were extracted on a daily basis at a spatial resolution of 1/12° from the Global Ocean Physics Analysis and Forecast of the EU Copernicus Marine Service Information (PHY_001_024; http://marine.copernicus.eu/services-portfolio/access-to-products).

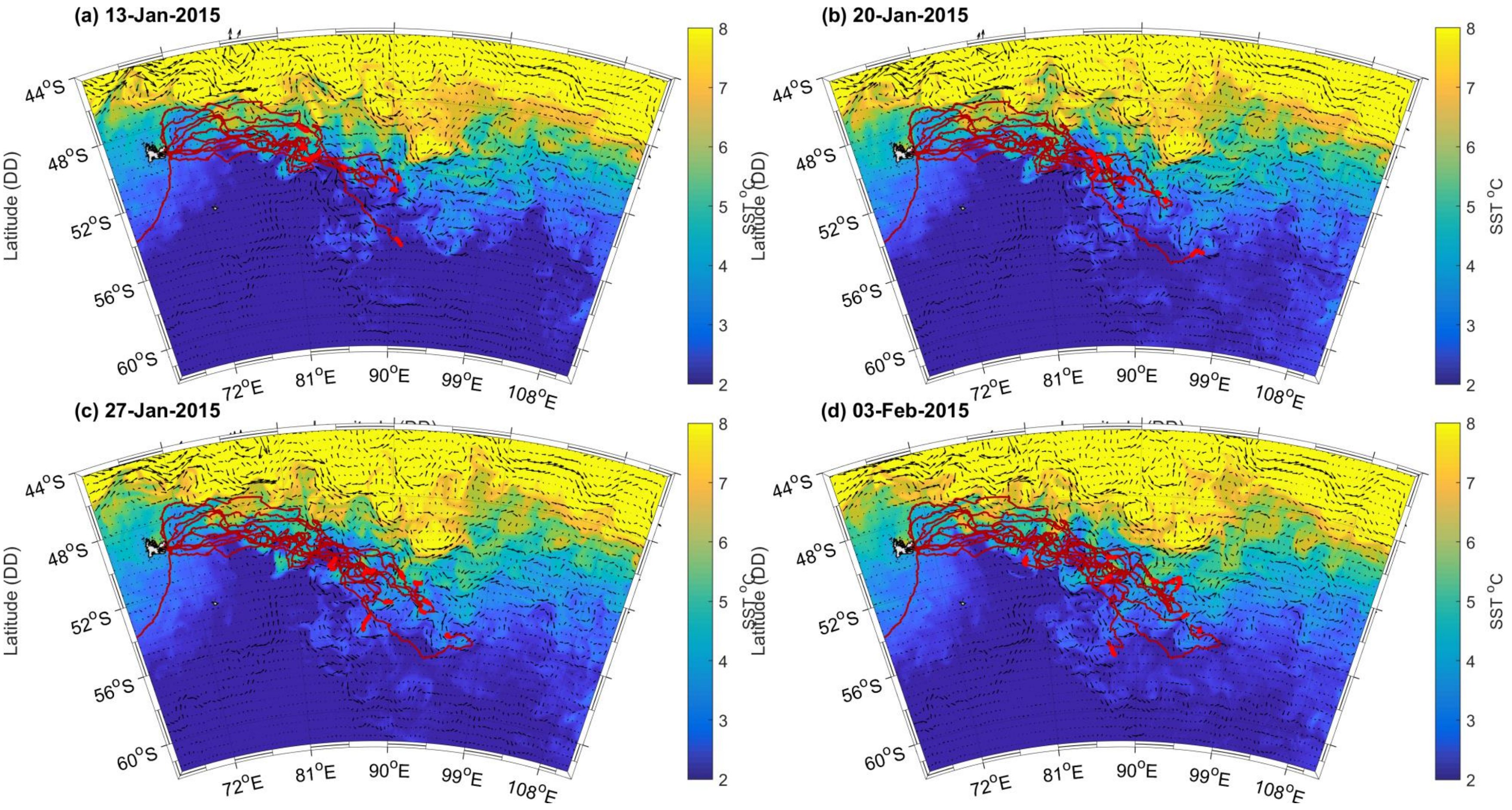

Figure S50. Trajectories of surviving pups on four days of the austral summer of 2015: (a) 13th January, (b) 20th January, (c) 27th January and (d) 3rd February. Bright red markers are locations concurrent to the day, and dark red lines, previous trajectories since leaving Kerguelen Islands. Black arrows are surface current directions with strength indicated by the size of the arrow (bolder, larger arrows are stronger currents). These were taken from the delayed time all-sat-merged Global Ocean Gridded Absolute Geostrophic Velocities Anomalies L4 product, obtained via the Archiving Validation and Interpretation of Satellite and Oceanographic data portal on a daily basis at a spatial resolution of 0.25° (AVISO; www.aviso.altimetry.fr). Background colours are sea surface temperatures (SST). These were extracted on a daily basis at a spatial resolution of 1/12° from the Global Ocean Physics Analysis and Forecast of the EU Copernicus Marine Service Information (PHY_001_024; http://marine.copernicus.eu/services-portfolio/access-to-products).

S.14 References

Barton, K. 2015. MuMIn. R.

Bhattacharyya, A. 1943. On a measure of divergence between two statistical populations defined by their probability distributions. Bulletin of the Calcutta Mathematical Society, 35: 99–109.

Biuw, M., McConnell, B., Bradshaw, C. J., and Fedak, M. 2003. Blubber and buoyancy: monitoring the body condition of free-ranging seals using simple dive characteristics. Journal of Experimental Biology, 206: 3405–3423.

Breed, G. A., Bowen, W. D., McMillan, J. I., and Leonard, M. L. 2006. Sexual segregation of seasonal foraging habitats in a non-migratory marine mammal. Proceedings of the Royal Society B, 273: 2319–2326.

Brooks, S. P., and Gelman, A. 1998. General methods for monitoring convergence of iterative simulations. Journal of Computational and Graphical Statistics, 7: 434–455.

Calenge, C. 2014. adehabitatHR. R.

Carpenter, B., Gelman, A., Hoffman, M. D., Lee, D., Goodrich, B., Betancourt, M., Brubaker, M., *et al.* 2017. Stan: A probabilistic programming language. Journal of Statistical Software, 76.

Carvalho, C. M., Polson, N. G., and Scott, J. G. 2010. The horseshoe estimator for sparse signals. Biometrika, 97: 465–480.

Cotté, C., Park, Y. H., Guinet, C., and Bost, C. A. 2007. Movements of foraging king penguins through marine mesoscale eddies. Proceedings of the Royal Society of Biological Sciences, 274: 2385–2391.

Cox, S. L., Orgeret, F., Gesta, M., Rodde, C., Heizer, I., Weimerskirch, H., and Guinet, C. 2018. Processing of acceleration and dive data on-board satellite relay tags to investigate diving and foraging performance in free-ranging marine predators. Methods in Ecology and Evolution, 9: 64–77.

Crainiceanu, C., Ruppert, D., and Wand, M. 2005. Bayesian analysis for penalized spline regression using WinBUGS. Journal of Statistical Software, 14: 1–24.

Fedak, M. A., Lovell, P., and Grant, S. M. 2001. Two approaches to compressing and interpreting time-depth information as collected by time-depth information and satellite-linked data recorders. Marine Mammal Science, 17: 94–110.

Fieberg, J., and Kochanny, C. O. 2005. Quantifying home-range overlap: the importance of the utilization distribution. The Journal of Wildlife Management, 69: 1346–1359.

Gaspar, P., Georges, J.-Y., Fossette, S., Lenoble, A., Ferraroli, S., and Le Maho, Y. 2006. Marine animal behaviour: neglecting ocean currents can lead us up the wrong track. Proceedings of the Royal Society B, 273: 2697–2702.

Gordine, S. A., Fedak, M., and Boehme, L. 2015. Fishing for drifts: detecting buoyancy changes of a top marine predator using a step-wise filtering method. Journal of Experimental Biology, 218: 3816–3824.

Jonsen, I. 2016. Joint estimation over multiple individuals improves behavioural state inference from animal movement data. Scientific Reports, 6: 20625.

Jonsen, I., Bestley, S., Wotherspoon, S., Sumner, M., and Flemming, J. M. 2017. Bayesian State-Space Models for Animal Movement (bsam). R.

Jonsen, I. D., Flemming, J. M., and Myers, R. A. 2005. Robust state-space modelling of animal movement data. Ecology, 86: 2874–2880.

Kooyman, G. L., and Ponganis, P. J. 1998. The physiological basis of diving to depth: birds and mammals. Annual Review of Physiology, 60: 19–32.

Mitani, Y., Andrews, R. D., Sato, K., Kato, A., Naito, Y., and Costa, D. P. 2010. Three-dimensional resting behaviour of northern elephant seals: drifting like a falling leaf. Biology Letters, 6: 163–166.

Nakagawa, S., and Schielzeth, H. 2013. A general and simple method for obtaining R2 from generalized linear mixed-effects models. Methods in Ecology and Evolution, 4: 133–142.

Orgeret, F., Cox, S. L., Weimerskirch, H., and Guinet, C. 2019. Body condition influences ontogeny of foraging behaviour in juvenile southern elephant seas. Ecology and Evolution, 9: 223–236.

Photopoulou, T., Lovell, P., Fedak, M. A., Thomas, L., and Matthiopoulos, J. 2015. Efficient abstracting of dive profiles using a broken-stick model. Methods in Ecology and Evolution, 6: 278–288.

Piironen, J., and Vehtari, A. 2017. Sparsity information and regularization in the horseshoe and other shrinkage priors. Electronic Journal of Statistics, 11: 5018–5051.

Pinheiro, J., and Bates, D. M. 2014. nlme: Linear and nonlinear mixed effects models. R.

Polson, N. G., and Scott, J. G. 2010. Shrink globally, act locally: sparse Bayesian regularization and prediction. *In* Bayesian Statistics 9, pp. 501–525. Ed. by J. M. Bernardo, M. J. Bayarri, J. O. Berger, A. P. Dawid, D. Heckerman, A. F. M. Smith, and M. West. Oxford University Press.

Yang, J., Zhu, H., Choi, T., and Cox, D. D. 2016. Smoothing and mean-covariance estimation of functional data with a Bayesian hierarchical model. Bayesian Analysis, 11: 649–670.

Zuur, A. F., Ieno, E. N., Walker, N. J., Saveliev, A. A., and Smith, G. M. 2009. Mixed effects models and extensions in ecology with R. Springer.
